# Separability of histopathology foundation model embeddings of stimulated Raman histology images for lung carcinoma subtyping

**DOI:** 10.7150/jca.135384

**Published:** 2026-09-12

**Authors:** Karl-Moritz Schröder, Uyen-Thao Le, Birte Ohm, Till Nicke, Martin Peter Pichotka, Martin Werner, Jürgen Beck, Jakob Straehle, Rainer Schmelzeisen, Leonard Brandenburg, Peter Bronsert, Andreas Weber-Steinhilber

**Affiliations:** 1 Institute for Surgical Pathology, Medical Center, University of Freiburg, Faculty of Medicine, Freiburg, Germany.; 2 Department of Thoracic Surgery, Medical Center, University of Freiburg, Freiburg, Germany.; 3 Fraunhofer Institute for Digital Medicine MEVIS, Bremen/Lübeck/Hamburg, Germany.; 4 Division of Medical Physics, Department of Diagnostic and Interventional Radiology, Medical Center, University of Freiburg, Freiburg, Germany.; 5 Center for Advanced Surgical Tissue Analysis (CAST), University of Freiburg, Freiburg, Germany.; 6 Core Facility for Histopathology and Digital Pathology, Medical Center, University of Freiburg, Freiburg, Germany.; 7 Tumorbank Comprehensive, Cancer Center Freiburg, Medical Center, University of Freiburg, Freiburg, Germany.; 8 Department of Neurosurgery, Medical Center, University of Freiburg, Freiburg, Germany.; 9 Department of Oral and Maxillofacial Surgery, Medical Center, University of Freiburg, Freiburg, Germany.

**Keywords:** deep learning, histopathology, stimulated Raman histology, foundation models, linear probing, lung carcinoma

## Abstract

Histopathology foundation models trained on hematoxylin and eosin (H&E) whole-slide images have recently emerged as versatile encoders for downstream diagnostic tasks, but their transferability to out-of-domain imaging modalities such as stimulated Raman histology (SRH) is unknown. Here, we retrospectively evaluated whether embeddings from histopathology foundation models enable intraoperative SRH-based classification of lung cancer patients into (i) non-small cell lung cancer (NSCLC) versus pulmonary metastasis (MET) and (ii) adenocarcinoma (AC) versus squamous cell carcinoma (SCC). For NSCLC versus MET, embeddings from histopathology foundation models showed separability in a range comparable to ResNet50. In contrast, for AC versus SCC, histopathology foundation models showed a tendency to outperform ResNet50, although without statistical significance. Overall performance remained too low to support clinical deployment. Nonetheless, our results indicate that histopathology foundation models could transfer discriminative representations to SRH images for specific intraoperative subtyping tasks.

## Introduction

Globally, lung cancer is responsible for the highest number of cancer-related deaths 1. Non-small lung cancer (NSCLC) is subclassified into adenocarcinoma (AC), squamous cell carcinoma (SCC) and large cell carcinoma (LC) 2. In addition, intrapulmonary metastases represent an important tumor entity with a wide range of primary tumor sites. For adenocarcinomas, primary tumors of the gastrointestinal tract, mammary gland, or thyroid must be considered, whereas squamous cell carcinomas frequently originate from the head and neck region or the esophagus. The differentiation between benign lesions (e.g., sarcoidosis, intrapulmonary lymph node or interstitial pneumonia), primary lung cancer and metastases have immediate therapeutic implications leading to different surgical treatment procedures 3. Especially for intraoperative decision-making the differentiation of primary lung cancer and metastasis is crucial. In patients undergoing curative resection of lung cancer a radical removal of the affected lung lobe and a systematic lymph node resection is usually required to enable accurate staging and potentially improve prognosis 4. In contrast, resection of metastases, particularly in peripheral regions of the lung, can be performed using wedge resection or laser enucleation with largely preserved healthy lung parenchyma. Furthermore, a patient with histologically complete (R0) resection of a pulmonary metastasis is usually subjected to further local treatment in case of recurrence while in primary lung cancer stage IIA and above, resection is followed by adjuvant therapy 3.

In the current management of intrapulmonary nodules, intraoperative frozen section analysis is used as a valuable tool for guiding intraoperative surgical decision-making. Intraoperative frozen section requires a comprehensive approach integrating clinical information, gross examination and intraoperative histological evaluation. Although frozen sections are highly useful, not all patients can be definitively addressed in this setting. Challenging lesions often require an additional workup comprising of paraffin-embedding, sectioning, staining and further examinations such as immunohistochemistry or molecular analyses 5. Intraoperative Stimulated Raman histology (SRH) represents an alternative to conventionally frozen sectioning. SRH rapidly delivers high-resolution images without prior time-consuming tissue processing and staining. SRH leverages stimulated Raman scattering (SRS) of photons which undergo an energy shift at specific wavenumbers when interacting with chemical bonds present in molecules such as DNA, proteins or lipids to visualize the cellular architecture of a tissue sample. Using the NIO Laser Imaging System (Invenio Imaging Inc., Santa Clara, CA, USA), the acquired SRS images are processed with a special look-up table into images resembling hematoxylin and eosin (H&E)-stained tissue sections. These SRH images can subsequently be analyzed and evaluated by pathologists 6, 7.

In recent years, Deep Learning (DL) was widely applied on SRH/SRS images with applications ranging from tissue classification for laryngeal 8 and oral squamous cell carcinoma 9, distinguishing benign and malignant cases of breast core-needle biopsies 10 or brain tumor detection and classification 11, 12. In the field of histopathology, foundation models have been trained on millions of whole-slide images, emerging as a promising way to capture histopathological knowledge from large-scale datasets. Clinical benchmarking has shown that these models can perform specific disease detection and biomarker prediction tasks. However, the composition of the pre-training dataset remains a critical factor in determining performance 13. Although the growing number of foundation models challenges the idea of a single and general solution, foundation models are expected to be applicable to a variety of downstream tasks, such as tumor subtyping and survival prediction. Applications of different foundation models range from ovarian cancer subtype classification 14 to lung cancer biomarker detection 15 or tasks like whole slide image retrieval 16. Although histopathology foundation models can be regarded in many ways as a success, the applicability of these models to out-of-domain data types like SRH images remains unknown.

The presented study investigates the applicability of histopathology foundation models on SRH images for classifying patients into two binary classes: Primary non-small cell lung cancer (NSCLC) and metastasis (MET). NSCLC was further subdivided into adenocarcinoma (AC) and squamous cell carcinoma (SCC). We perform linear and non-linear probing on SRH image patch embeddings from different foundation models to test the hypothesis that foundation models, used as frozen encoders, are able to encode a discriminative signal into embeddings from SRH patches for separating the aforementioned classes.

## Materials and Methods

### Study protocol and sample acquisition

Ethical approval was obtained from the Ethics Committee of the Medical Center, University of Freiburg (22-1322_2-S1). Patient consent was obtained before inclusion. Participants were Individuals of legal age (>18 years) with confirmed primary lung cancer or pulmonary metastases, who had not received prior neoadjuvant therapy and were eligible for surgical resection. All surgical procedures were performed at the Department of Thoracic Surgery, Medical Center, University of Freiburg. Subsequent histopathological confirmation was performed at the Department for Surgical Pathology, Medical Center, University of Freiburg.

### Cohort characteristics

The cohort consisted of 93 patients in total, with 68 patients diagnosed with NSCLC, of which 37 were SCC and 31 AC. 25 patients were diagnosed with metastatic disease, from which 12 AC were derived from a colorectal primary, two AC from the mammary gland, one AC from the pancreas and two AC from contralateral lung cancers, as well as two SCC from the oropharynx and one SCC from the contralateral lung and five from other primary sites. The classification of tumor subtypes SCC, AC and metastases were validated using immunohistochemistry and in case of metastases of SCC, correlation with clinical data was performed.

### SRH image acquisition

SRH images were acquired using the methods published by 17. In short, pathologists identified tumor-suspected and non-neoplastic areas. Tissue specimens were obtained from these areas. These fresh tissue samples were scanned using a NIO Laser Imaging System (Invenio Imaging Inc., Santa Clara, CA, USA) to generate SRH images, resembling H&E sections, as previously described 6. The fiber laser acquires line scans spanning 1000 pixels in width with a pixel size of 467 nm and obtained values for Raman shifts at 2845 cm^-1^ and 2930 cm^-1^ per pixel. It measures with a pixel dwell time of approximately 1.6 µs/pixel. Spectral resolution is up to 0.1 nm or 1 cm^-1^. A vendor-specific look-up table converts raw pixel values into SRH images. No further preprocessing was applied to the SRH images.

### Histopathological evaluation of SRH images

To evaluate SRH-images, all images were transferred to QuPath, version 0.4.3 18. Each image was annotated with differing tumors (previously confirmed malignancy NSCLC or metastasis; see Figure 1) and normal tissue. The normal tissue was subdivided into fat, stroma, necrosis and normal lung parenchyma. All annotations were transcribed into the GeoJSON format and transferred to foundation models for training.

### Feature extraction

Patches of size 250x250 pixels were extracted if they spatially overlapped with tumor annotations by at least 99%. A 99% threshold ensured that patches consisted almost entirely of labeled pixels accounting for potential labeling or overlap computing errors. This strategy minimized unlabeled or conflicting information that could complicate the embedding creation of the model. These patches were then used as input for histopathology foundation models to produce patch embeddings. We compared CTransPath 19, CONCH 20 H-Optimus-0 21, H-Optimus-1 22, Hibou-L 23, Kaiko 24, Phikon 25, ProvGigaPath 26, TissueConcepts 27, UNI2-h 28 and Virchow2 29. All foundation models were compared to a ResNet50 pretrained on ImageNet as a baseline. Patch preprocessing followed the individual requirements of the respective foundation model. All foundation models were frozen and used as feature extractor for the preprocessed patches. Linear probing was performed on all labelled patch embeddings using a logistic regression from scikit-learn 30. To additionally assess the non-linear separability of the embeddings, a 2-layer multi-layer perceptron (MLP) was trained. Hyperparameter details of each training architecture can be found in Table 1. The data were split at the patient level into class-stratified 70% training set and 30% test set. Figure 2 shows the class distribution at patch, slide and patient levels. The distribution of number of patches per patient are shown as histograms in supplementary Figure S9.

### Training procedure

For linear probing, 10-fold cross-validation with three seeds per fold was used. The model that performed best on the validation set out of the three seeds was used for testing. Ten trained models were tested on the hold-out test set for each foundation model architecture (one model for each fold). Performance variation was estimated by standard deviation from the mean across 10 folds. For MLP, one model was trained on the training set and the model with the highest balanced accurracy during training was evaluated on the test set. Class imbalance was addressed using inverse-frequency class weights in the cross-entropy loss. Performance uncertainty was estimated by percentile bootstrapping over test samples with 1,000 iterations. Performance metrics are primarily reported at patient-level. Additional performance metrics at patch- and slide-level can be found in the supplements. To aggregate the patch-level predictions to the slide- and patient-levels, probability averaging of the individual patch predictions was performed. The per-class metrics are precision (PRE), recall (REC), and F1-score (F1), while class-aggregated metrics are balanced accuracy (BA), Matthews correlation coefficient (MCC), and area under the receiver operating characteristic curve (AUROC). Recall is equivalent to sensitivity and in case of a binary classifier the sensitivity for a given class is equivalent to the specificity of the opposite class.

### Hypothesis testing

For statistical comparison of the metrics BA and MCC, predictions were averaged across the ten probes for logistic regression to obtain one architecture-level prediction per test sample. Paired bootstrap testing was performed on the test-set predictions for each foundation model against the ResNet50 baseline. For AUROC, average values for each architecture were calculated using average predictions across the 10 folds for logistic regression. Each foundation model was then compared to the ResNet50 baseline with a paired DeLong test. To account for multiple comparisons, p-values were adjusted using the Holm-Bonferroni method. Correction was applied separately for each classification strategy (i.e., linear probing and MLP), architecture (i.e., ResNet50 versus all foundation models), and performance metric (i.e., BA, AUROC and MCC). To reject the null hypothesis a significance threshold of α=0.05 was selected. A model was considered to perform statistically significant different compared to ResNet50 when the adjusted p-value was below the predefined significance threshold α.

## Results

### Linear probing MET vs. NSCLC

For linear classification of MET and NSCLC, embeddings from ResNet50 yielded a mean BA of 0.64 ± 0.04, mean AUROC of 0.82 ± 0.03 and mean MCC of 0.35 ± 0.12. The highest mean BA and MCC was achieved by embeddings from Virchow2 (mean BA=0.73 ± 0.04; mean MCC=0.61 ± 0.07) and the highest mean AUROC was achieved by embeddings from hibou-L (mean AUROC=0.87 ± 0.06). In all cases, performance of the logistic regression on foundation model embeddings showed no statistically significant difference compared to performance metrics on ResNet50 embeddings. Figure 3 shows violin plots for all performance metrics on patient-level. Additional performance metrics on patch- and slide-level are in supplements Figures S1 and S2. Detailed performance metric values for all levels are in supplements Tables S1 - S36 and p-values are in supplements Table S75.

### Linear probing AC vs. SCC

For the linear classification of AC and SCC, embeddings from ResNet50 yielded a mean BA of 0.52 ± 0.08, mean AUROC of 0.55 ± 0.06 and mean MCC of 0.04 ± 0.17. The highest mean BA and MCC was achieved by embeddings from H-optimus-0 (mean BA=0.69 ± 0.07; mean MCC=0.37 ± 0.14) and the highest mean AUROC was achieved by embeddings from H-optimus-1 (mean AUROC=0.75 ± 0.04). In all cases, performance of the logistic regression on foundation model embeddings showed no statistically significant difference compared to performance metrics on ResNet50 embeddings. Figure 4 shows violin plots for all performance metrics on patient-level. Additional performance metrics on patch- and slide-level are in supplements Figures S5 and S6. Detailed performance metric values for all levels are in supplements Tables S38 - S73 and p-values are in supplements Table S76.

### Non-linear probing NSCLC vs. MET

For the non-linear classification of NSCLC and MET, embeddings from ResNet50 yielded a BA of 0.72 (95% CI: [0.50-0.91]), AUROC of 0.82 (95% CI: [0.63-0.96]) and MCC of 0.42 (95% CI: [-0.01-0.75]). The highest BA and MCC was achieved by embeddings from Virchow2 (BA=0.75 (95% CI: [0.57-0.94]) and MCC=0.65 (95% CI: [0.33-0.92])) and the highest AUROC was achieved by embeddings from TissueConcepts (AUROC=0.93 (95% CI: [0.80-1.00])). In all cases, performance of the MLP on foundation model embeddings showed no statistically significant difference compared to performance metrics on ResNet50 embeddings. Figure 5 shows plots for all performance metrics on patient-level. Additional performance metrics on patch- and slide-level are in supplements Figures S3 and S4. Detailed performance metric values for all levels are in supplements Table S37 and p-values are in supplements Table S75.

### Non-linear probing AC vs. SCC

For the non-linear classification of NSCLC and MET, embeddings from ResNet50 yielded a BA of 0.57 (95% CI: [0.32-0.80]), AUROC of 0.60 (95% CI: [0.32-0.86]) and MCC of 0.14 (95% CI: [-0.33-0.59]). The highest BA and MCC was achieved by embeddings from hibou-L (BA=0.74 (95% CI: [0.51-0.92]) and MCC=0.47 (95% CI: [0.02-0.81])) and the highest AUROC was achieved by embeddings from H-optimus-1 (AUROC=0.78 (95% CI: [0.54-0.98])). In all cases, performance of the MLP on foundation model embeddings showed no statistically significant difference compared to performance metrics on ResNet50 embeddings. Figure 6 shows plots for all performance metrics on patient-level. Additional performance metrics on patch- and slide-level are in supplements Figures S7 and S8. Detailed performance metric values for all levels are in supplements Table S74 and p-values are in supplements Table S76.

## Discussion

In this study, we applied histopathology foundation models to SRH images and investigated whether patch embeddings contained discriminative signals by classifying lung cancer patients as either NSCLC/MET or SCC/AC using linear probing with logistic regression and non-linear probing with a 2-layer MLP. We compared the separability of patch embeddings from various histopathology foundation models with embeddings from a ResNet50 model pretrained on ImageNet as a baseline to evaluate the transferability of the foundation model´s encodings to SRH imaging data.

Embeddings obtained from histopathology foundation models were found to be linearly separable in a similar range when classifying patients into NSCLC and MET compared to ResNet50 embeddings. For the non-linear classification with a MLP, performances on foundation model embeddings are as well in the same range compared to ResNet50 embeddings. Performance metrics showed no statistically significant differences between the foundation model embeddings and the ResNet50 baseline embeddings. Given the relatively small data set, this is a first indication towards the claim that histopathology foundation models are unable to encode SRH patches into embeddings containing discriminatory signals with respect to NSCLC and MET classes, and do not offer an advantage over a standard ResNet50 encoder pretrained on natural images. A differentiation of NSCLC versus MET is crucial for operative procedures. While especially peripherally located metastases are usually treated surgically by wedge resection or laser enucleation saving healthy lung parenchyma, radical surgery with lobectomy and lymphadenectomy is indicated in NSCLC depending on tumor location, size and patient conditions 3, 4. Studies attempting to subclassify NSCLC into primary tumor and metastasis based on imaging data mostly rely on computed tomography (CT) or positron emission tomography (PET). For example, Araujo-Filho et al. conclude that CT images do actually contain discriminatory characteristics 31. For H&E images, a classification between primary and metastatic lung tumors using deep learning models was achieved with a F1-Score of 0.83 for discrimination of primary versus metastatic 32. Also, for Cancer of unknown Primary (CUP) deep learning was used to predict primary sites of metastases on H&E-stained whole slide images, immunohistochemical staining and clinical correlation with an accuracy up to 95.5% 33. However, to the best of our knowledge, this is the first study attempting a subclassification of NSCLC into primary tumor and metastasis with histopathological foundation models leveraging SRH images.

When classifying patients into AC and SCC, embeddings obtained from histopathology foundation models show a tendency towards a better linear separability compared to ResNet50 embeddings. However, differences in performance metrics are not statistically significant. For the non-linear classification with a MLP, performances on foundation model embeddings are in the same range compared to ResNet50 embeddings. This suggests that histopathology foundation models could encode SRH patches into embeddings containing discriminatory information with respect to the classes AC and SCC, offering an advantage over a standard ResNet50 encoder. However, given the small number of patients and the statistical insignificance, this study is only a first indication towards that claim. Several studies have demonstrated the ability of deep learning algorithms to distinguish between AC and SCC using histological whole slide images 34, 35, 36, 37. However, this is the first study investigating a classification on SRH images for the intraoperative distinction between AC and SCC. Improving the differentiation between SCC and AC using histopathological foundation models could have a significant clinical impact, as different treatment options would need to be considered. Especially regarding harboring different genetic alterations of these two subtypes, different target therapeutic options can be evaluated 38. With this knowledge the intraoperative differentiation of subtypes is crucial to initiate molecular analyses immediately and save time and resources and enable prompt initiation of therapy. Furthermore, these two cancers should be analyzed separately to enable more precise prediction of the outcome 39.

For a realistically change of intraoperative decision-making, the performance threshold of the AI-assisted SRH-model should be at least equivalent or preferably higher that of frozen section, which currently represents the standard method for intraoperative tissue evaluation. According to the literature, the diagnostic accuracy of frozen section ranges between 83.3% and 99.4% 40, 41, 42. Furthermore, turnaround time is a critical factor in intraoperative tissue evaluation. For frozen section analyses, the time interval between the surgeon´s sample excision of the specimen and the communication of the diagnosis by the pathologist should not extend beyond 20 minutes 43. The scanning time for the specimens for SRH images was up to 22.8 minutes, depending on number of images acquired for a given sample.

This study has several limitations. Firstly, the data set contains only a small number of patients: 68 were patients classified as NSCLC, 25 patients classified as MET, 37 patients classified as SCC, and 31 patients classified as AC. Therefore, variations due to training set and test set sampling is high and the small observed changes in performance metrics between the architectures could not be backed up with statistical significance. Especially for distinguishing primary NSCLC from metastasis, the classification task can be regarded as complicated given the heterogenous underlying biology of metastasis. The lung is a common site for metastases for a wide range of primary tumours originating from different lineages and exhibiting divers' biological characteristics, reflected by distinct histomorphological, immunhistochemical and molecular profiles 2. Accordingly, our cohort included a limited number of metastatic cases from different primary sites (colorectal, breast, pancreatic, contralateral lung and oropharyngeal carcinomas). This heterogeneity may have introduced confounding effects and contributed to limited statistical power of the analysis.

Due to the complexity of the task, building a highly performant (AUC > 0.90) classifier was not feasible. Additionally, as this study is monocentric, with the training and test sets originating from the same institution, the results may be subject to overestimation of generalization performance. However, this does not affect the patch embedding quality and separability with linear and non-linear probing. Additionally, histopathology foundation models are mostly trained on H&E images rather than SRH although SRH aims to mimic the H&E stain.

To comprehensively assess the transferability of applying histopathology foundation models to SRH images, larger data sets and more diverse evaluation tasks are needed. Taking into account the differences between SRH and H&E images, establishing an SRH-dedicated foundation model trained exclusively on SRH images can be considered. However, this study represents a first step towards the application of histopathology foundation models to out-of-training domain imaging data.

## Conclusion

This study compared the linear and non-linear separability of histopathology foundation models embeddings from of SRH image patches for classifying patients as either NSCLC/MET or AC/SCC. While histopathology foundation models are unable to encode discriminatory signals for the classification of NSCLC and MET, embeddings for AC and SCC showed a tendency of higher linear separability compared to the ResNet50 ImageNet baseline. While performance differences showed no statistical significance for all tasks and overall performance remains far from being clinically applicable, this study marks an initial effort to extend histopathology foundation models to imaging data beyond their training domain.

## Supplementary Material

Supplementary figures and tables.

## Figures and Tables

**Figure 1:**
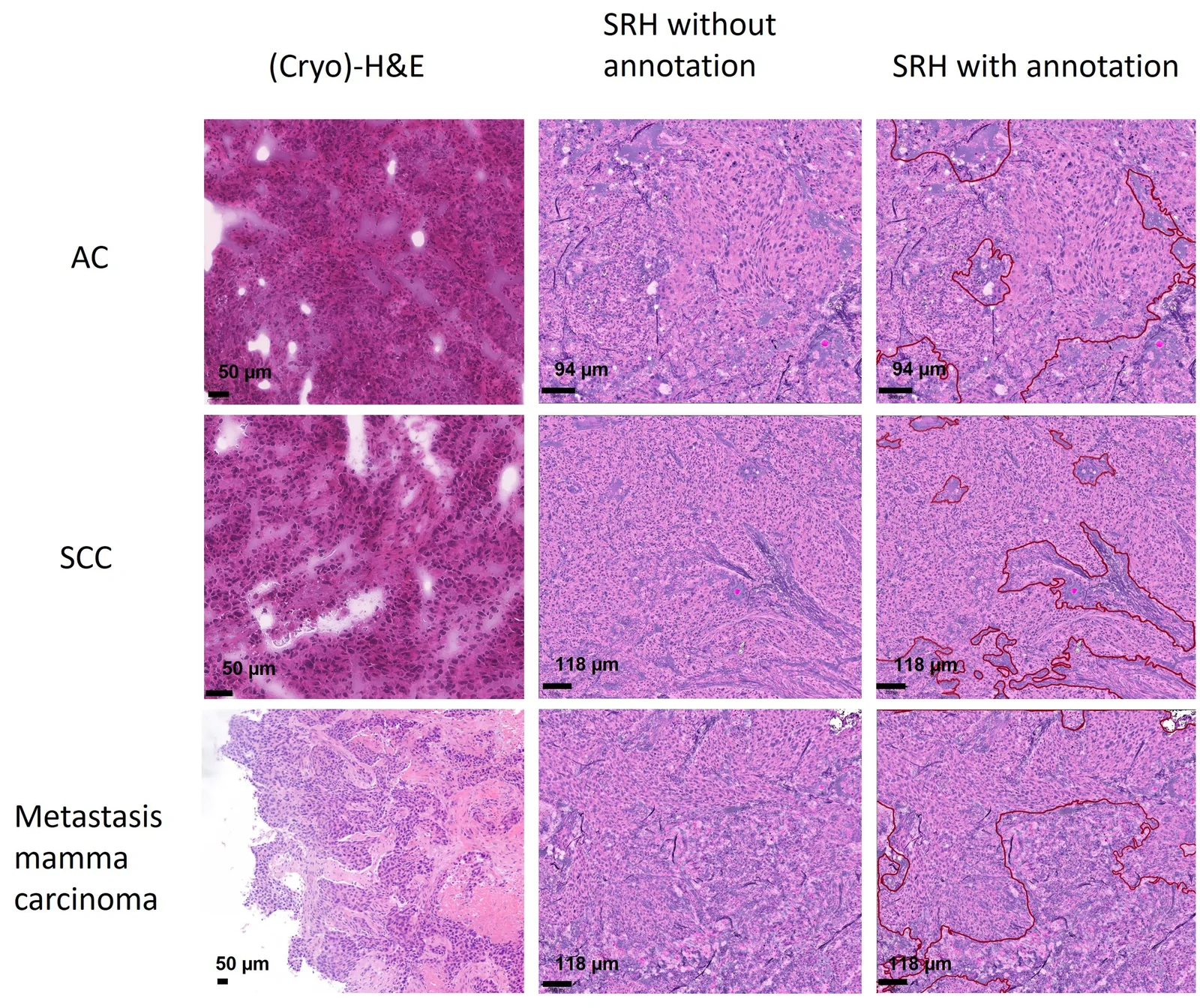
Comparison between different tumor-types (primary adenocarcinoma (AC), squamous cell carcinoma (SCC) and metastasis of mamma carcinoma). Classical (Cryo-) H&E first column, SRH images without annotation (second column), SRH-images with tumor-annotations (red lined, third column).

**Figure 2:**
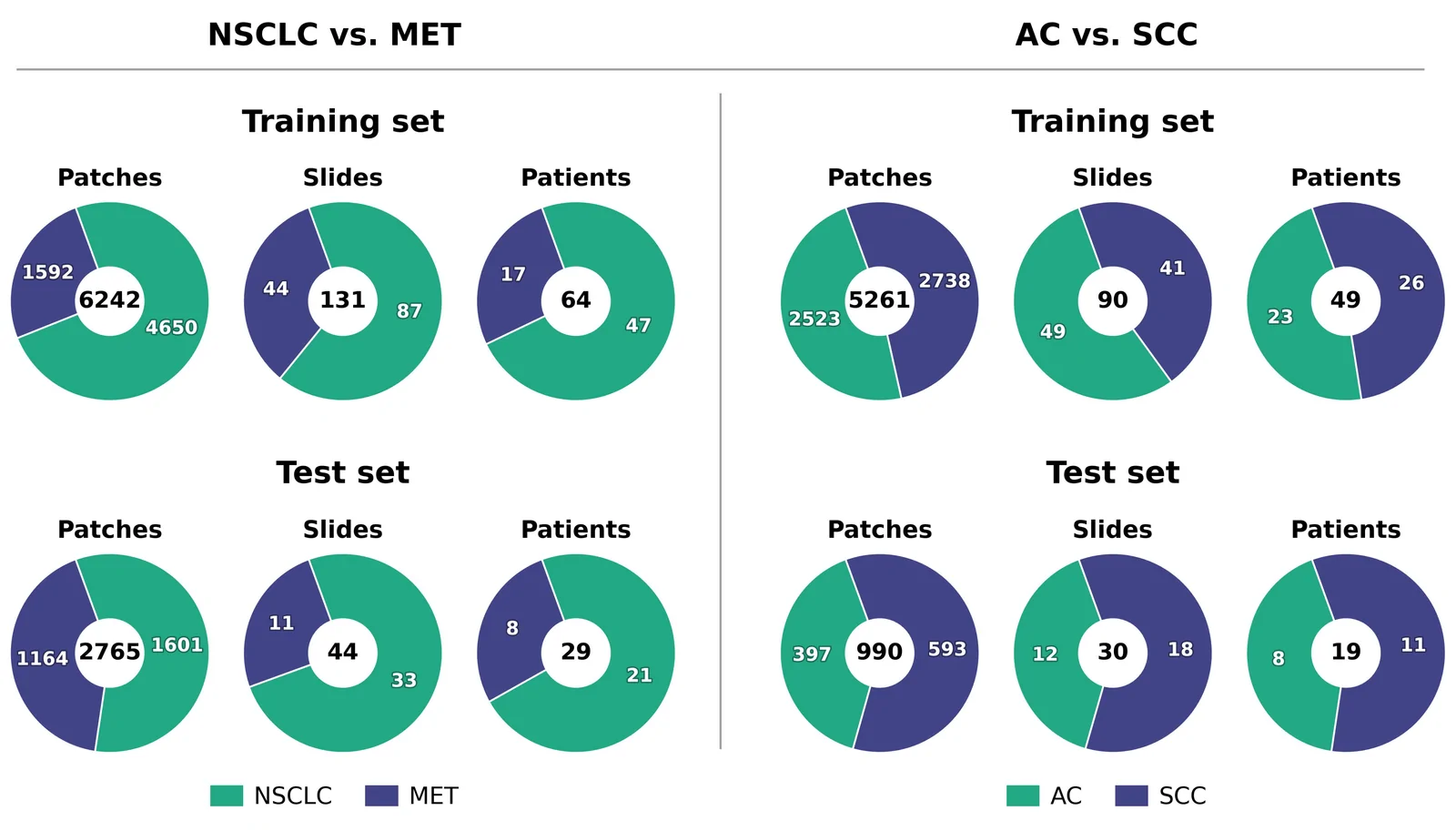
Class distributions for training and test set on patch-, slide- and patient-level of NSCLC vs. MET and AC vs. SCC.

**Figure 3:**
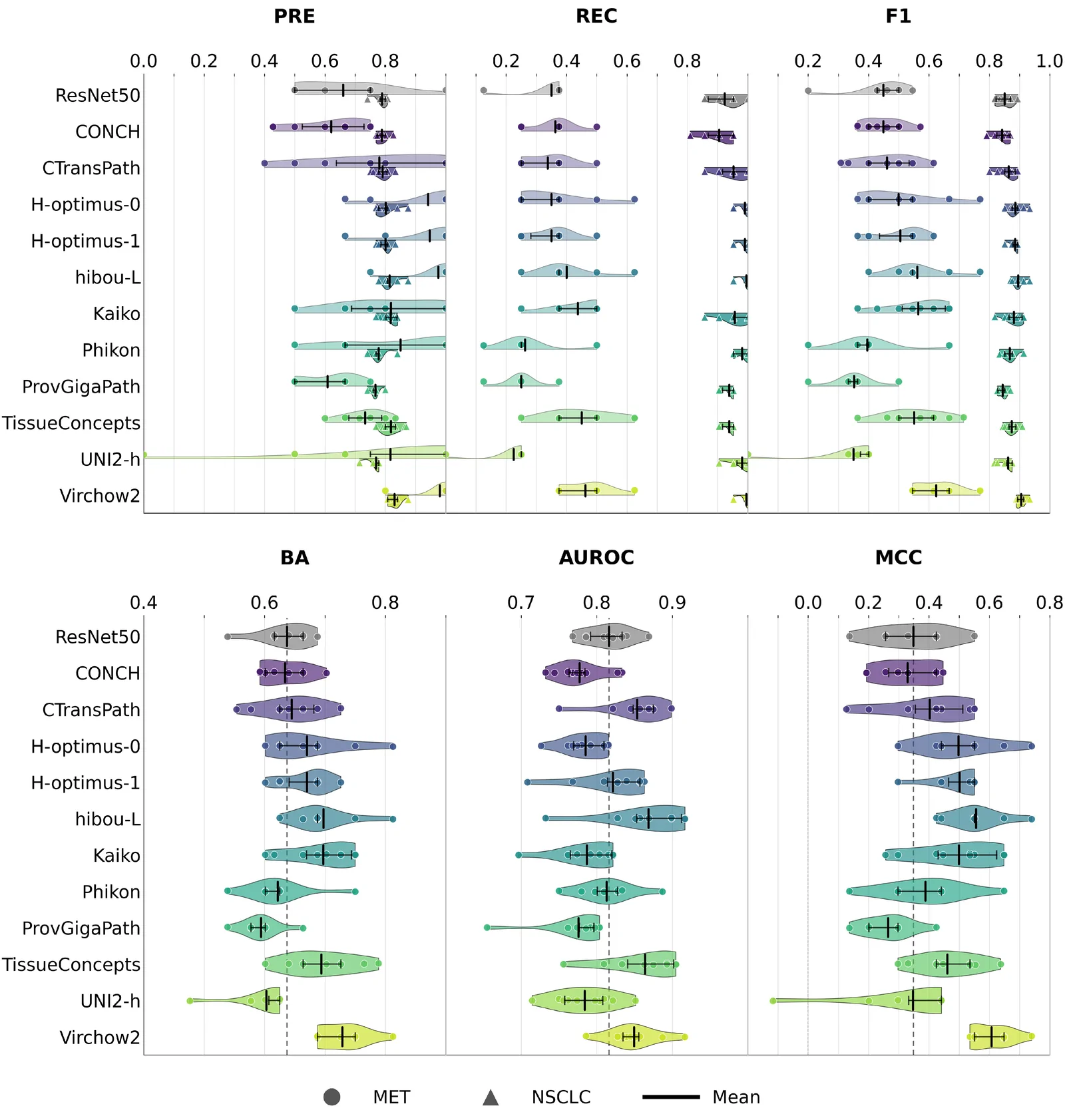
Performance metrics precision (PRE), recall (REC), F1-score (F1), balanced accuracy (BA), area under the ROC curve (AUROC) and Matthew's correlation coefficient (MCC) on patient level for distinguishing primary tumor (NSCLC) from metastasis (MET). For PRE, REC, and F1-score metrics are reported for each class separately. Horizontal error bars within each violin indicate the interquartile range, vertical black lines indicate mean values. The performance of the ResNet50 baseline is shown as a vertical dashed line.

**Figure 4:**
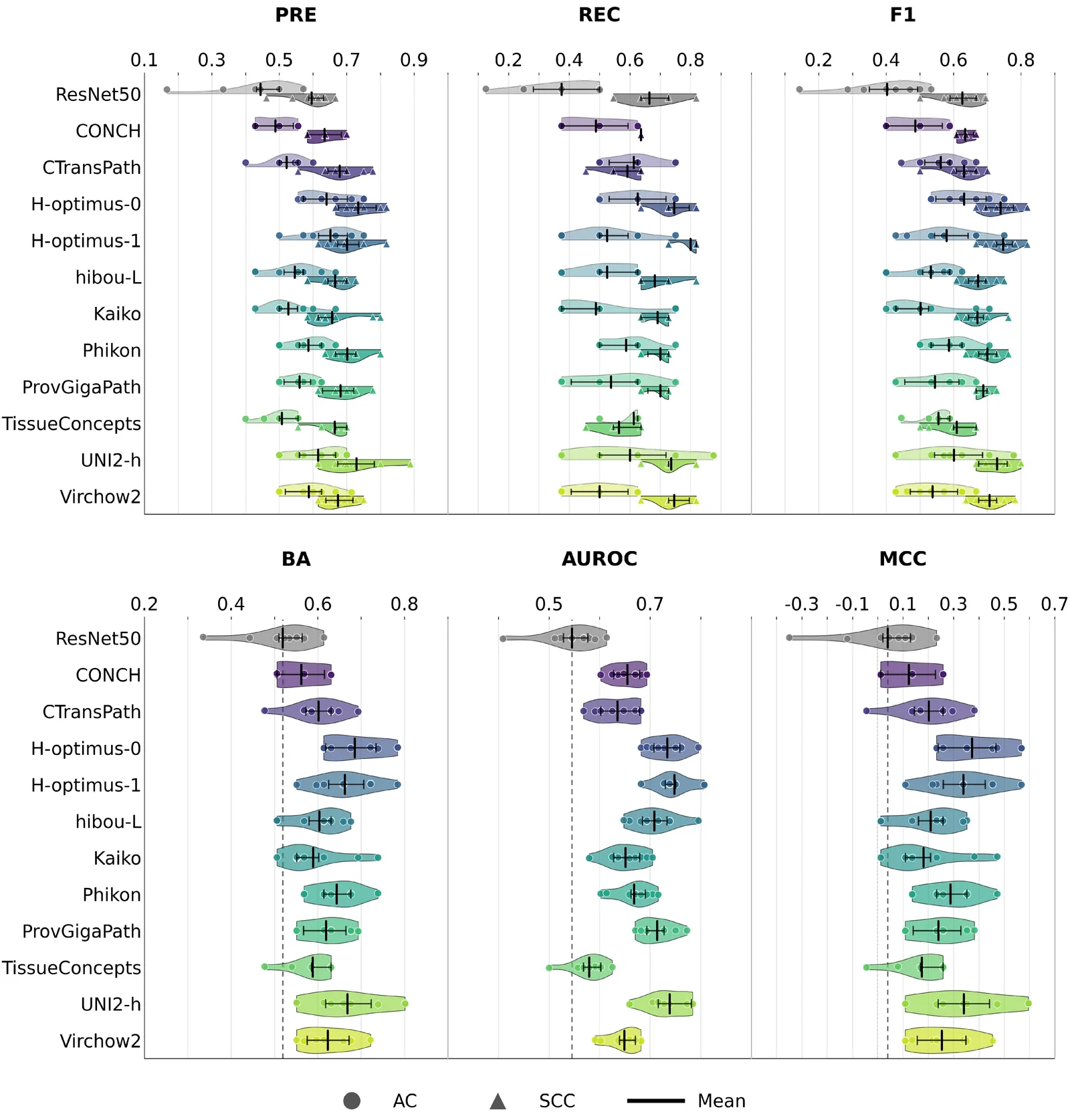
Performance metrics precision (PRE), recall (REC), F1-score (F1), balanced accuracy (BA), area under the ROC curve (AUROC) and Matthew's correlation coefficient (MCC) on patient level distinguishing adenocarcinoma (AC) from squamous cell carcinoma (SCC). For PRE, REC, and F1-score metrics are reported for each class separately. Horizontal error bars within each violin indicate the interquartile range, vertical black lines indicate mean values. The performance of the ResNet50 baseline is shown as a vertical dashed line.

**Figure 5:**
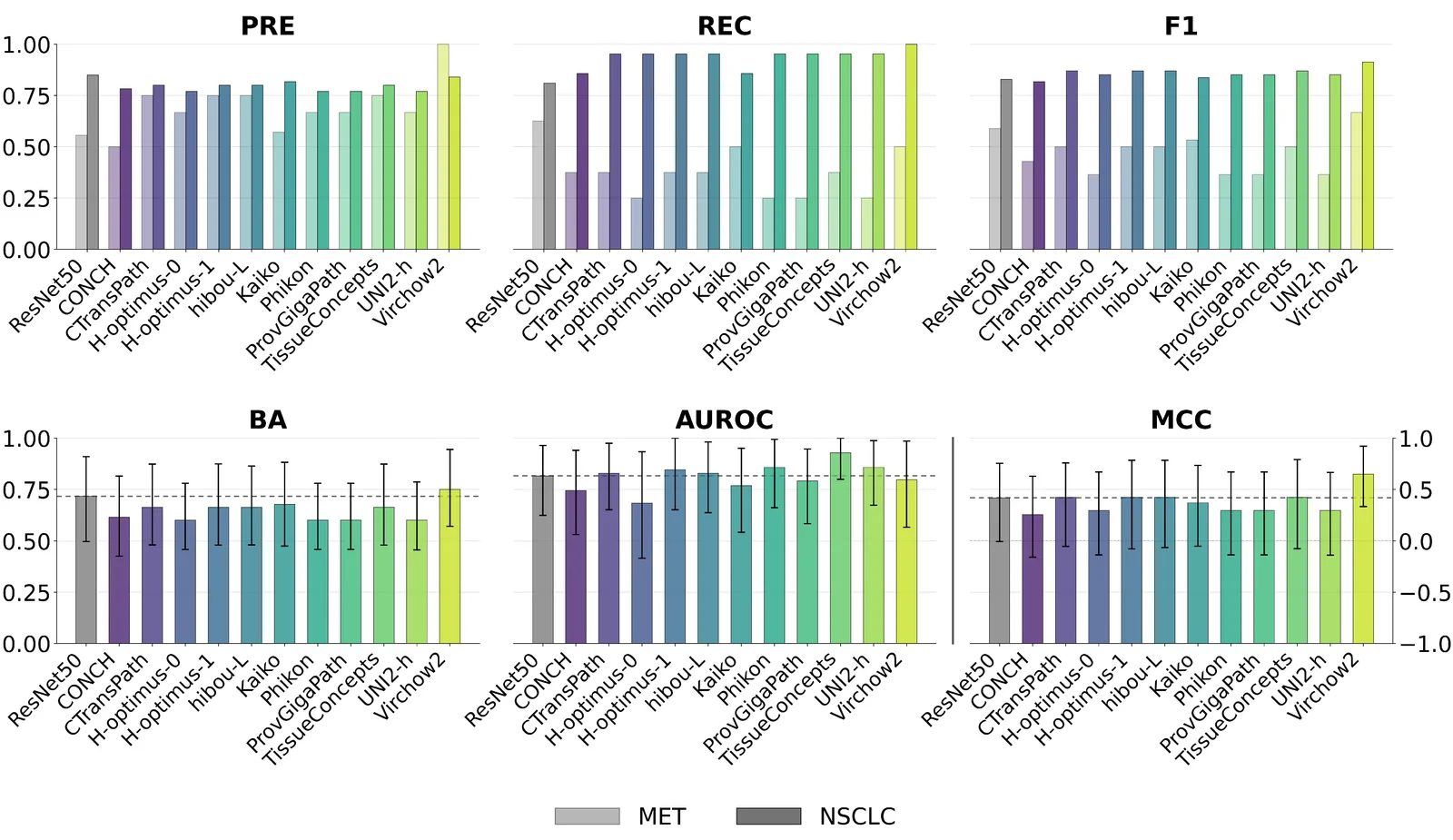
Performance metrics precision (PRE), recall (REC), F1-score (F1), balanced accuracy (BA), area under the ROC curve (AUROC) and Matthew's correlation coefficient (MCC) on patient level distinguishing metastasis (MET) from non-small cell lung cancer (NSCLC). For PRE, REC, and F1-score metrics are reported for each class separately. For BA, AUROC and MCC, error bars are bootstrapped 95% CI. The performance of the ResNet50 baseline is shown as a horizontal dashed line.

**Figure 6:**
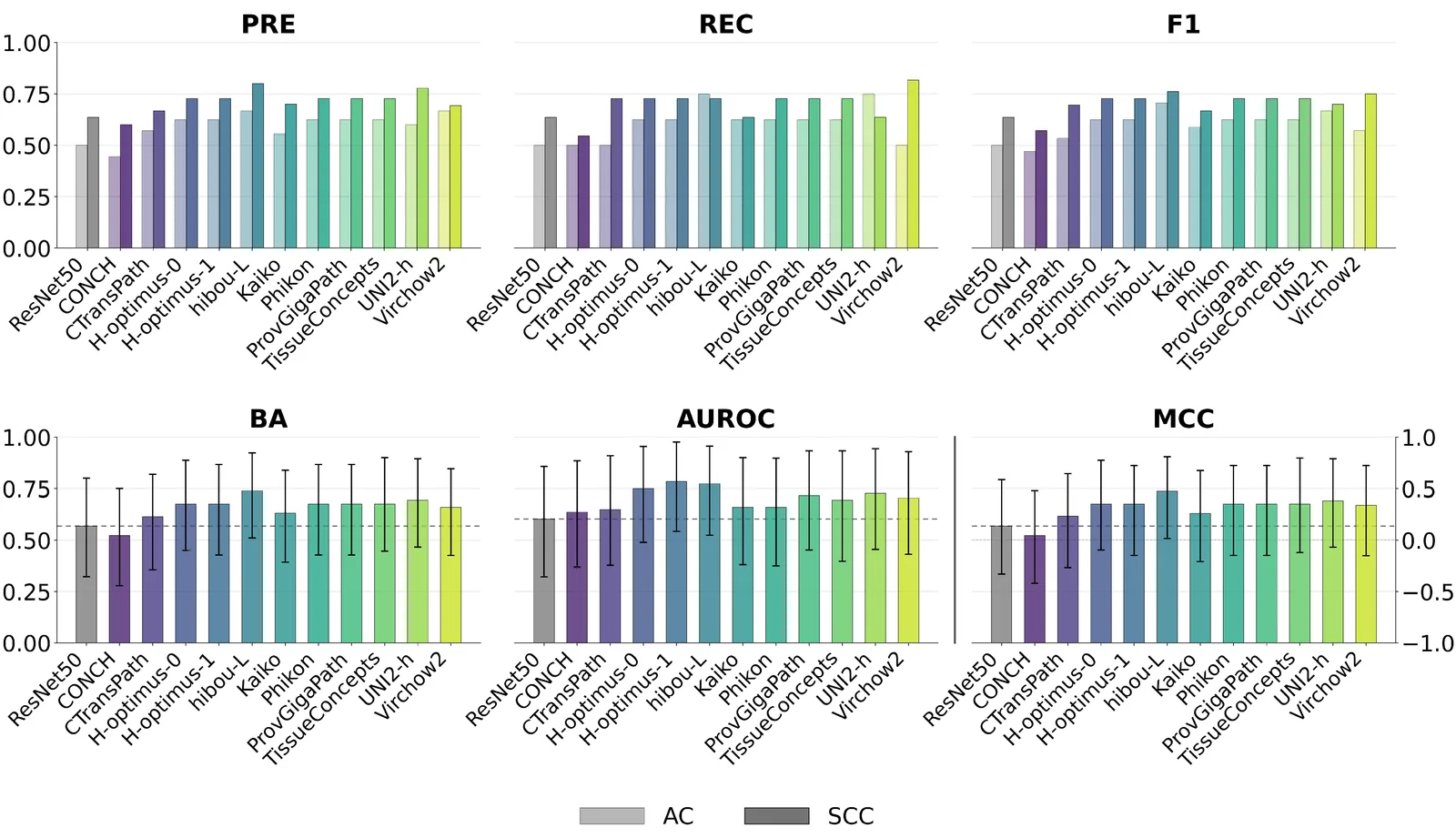
Performance metrics precision (PRE), recall (REC), F1-score (F1), balanced accuracy (BA), area under the ROC curve (AUROC) and Matthew's correlation coefficient (MCC) on patient level distinguishing adenocarcinomoa (AC) from squamous cell carcinoma (SCC). For PRE, REC, and F1-score metrics are reported for each class separately. For BA, AUROC and MCC, error bars are bootstrapped 95% CI. The performance of the ResNet50 baseline is shown as a horizontal dashed line.

**Table 1:** Hyperparameters of the logistic regression and MLP models.

Logistic regression	
Hyperparameter	Setting
Max_iter	1000
Class_weights	inverse frequency
Penalty	L2
C	0.1
Solver	liblinear
MLP	
Hyperparameter	Setting
Number of layers	2
Hidden dimension	256
Dropout	0.5
Epochs	50
Optimizer	AdamW
Learning rate	0.001
Betas	(0.9, 0.999)
Weight decay	0.01
Early stopping	10 epochs
Activation function	ReLU
Class weights	Inverse frequency
Loss	Cross-entropy
Batch size	128

## Data Availability

The datasets analyzed during the current study are not publicly available due to data protection regulations and the sensitive nature of medical imaging data. Access may be granted upon reasonable request to the corresponding author, subject to institutional approval, ethical review, and applicable data-sharing agreements.
