# Supplementary material for: Separability of histopathology foundation model embeddings of stimulated Raman histology images for lung carcinoma subtyping
Source: J Cancer. 2026 Sep 12;17(10):1698–708. doi: 10.7150/jca.135384 (PMC13617412; doi:10.7150/jca.135384)
Supplement: Supplementary file 1 [file jcav17p1698s1.pdf]

## *Supplements*

### 1. Performance metrics plots NSCLC vs. MET

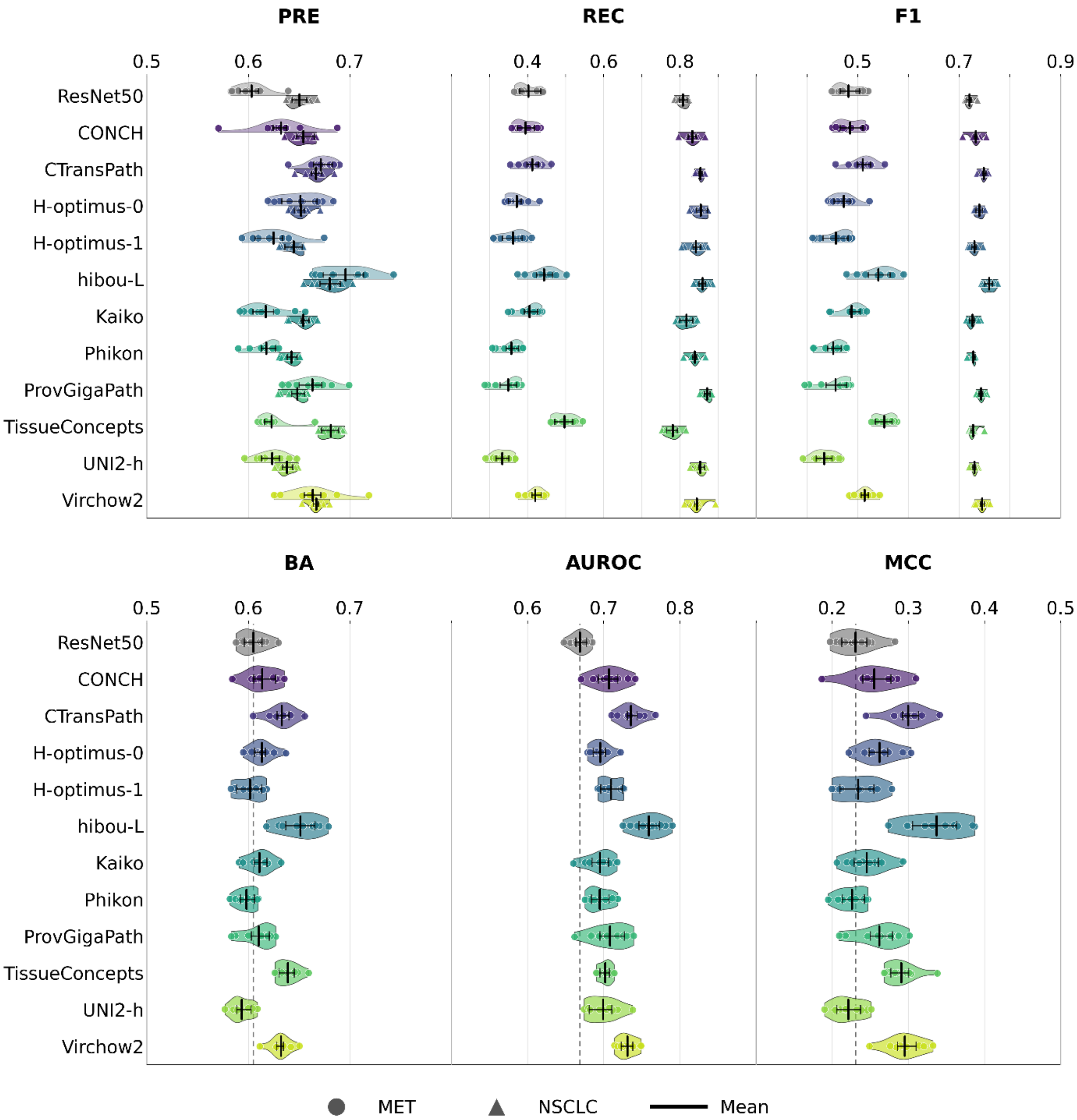


*Figure S1: Logistic regression: Patch-level performance metrics NSCLC vs. MET.*

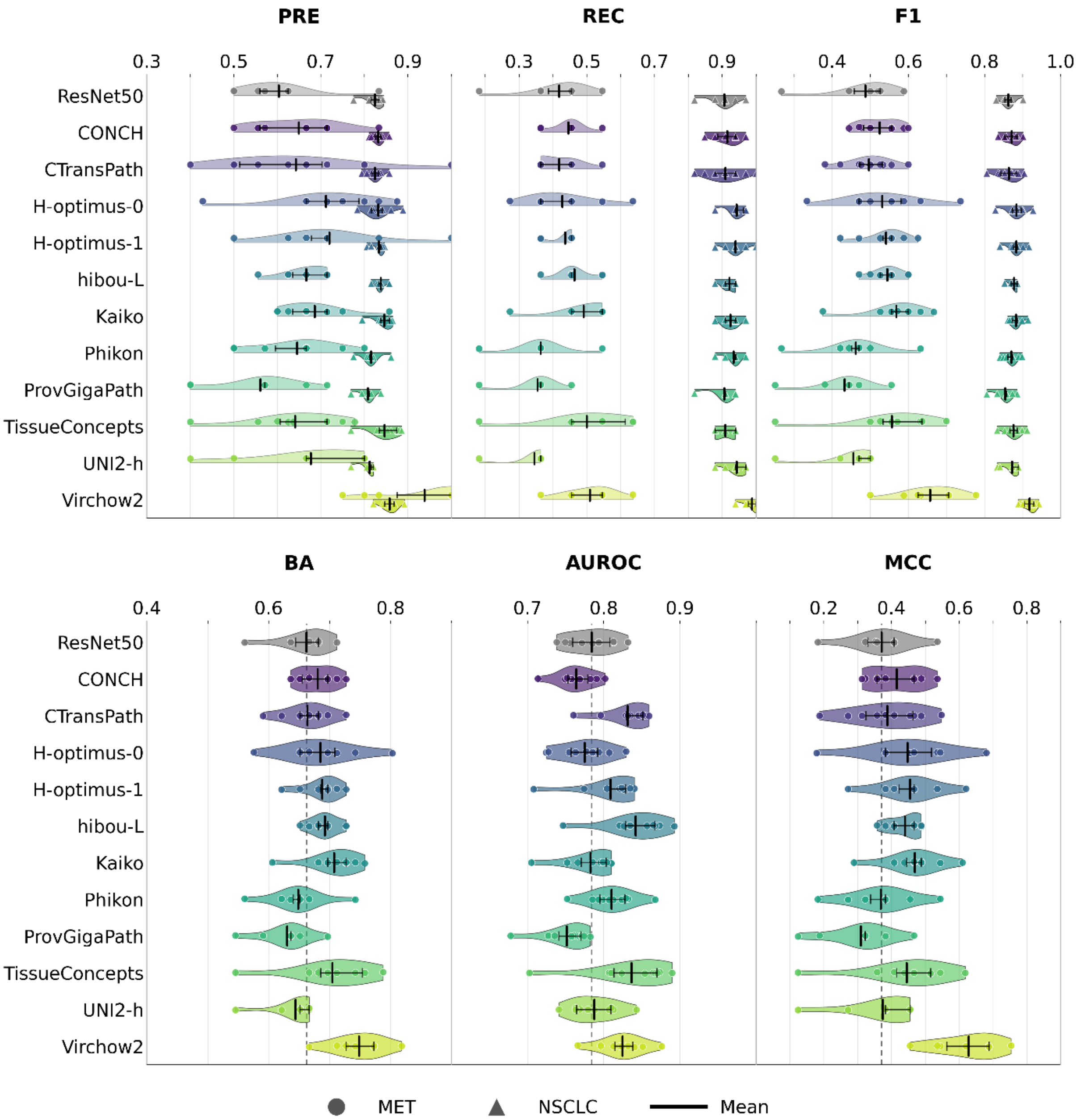


*Figure S2: Logistic regression: Slide-level performance metrics NSCLC vs. MET.*

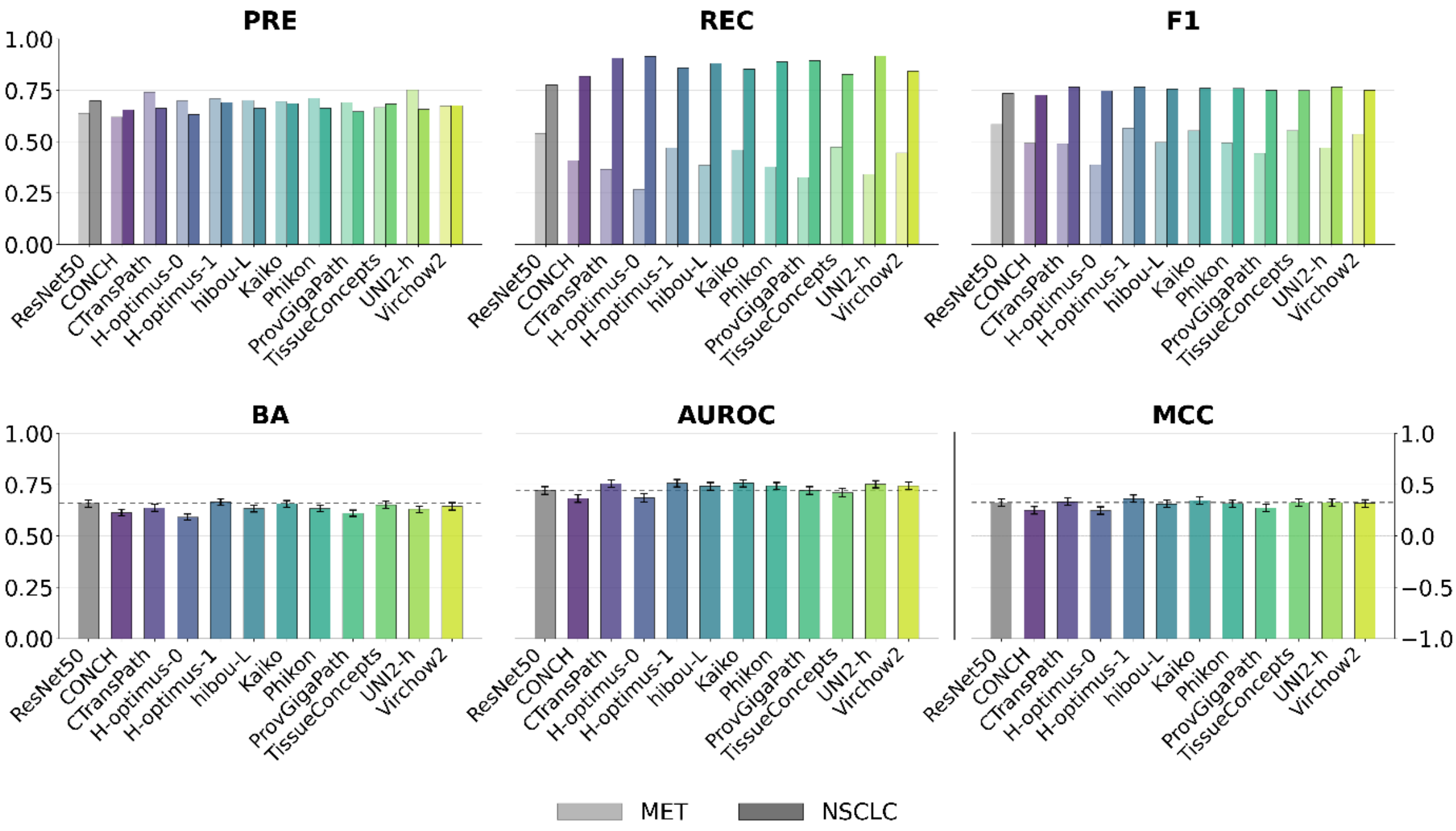


*Figure S3: MLP: Patch-level performance metrics NSCLC vs. MET.*

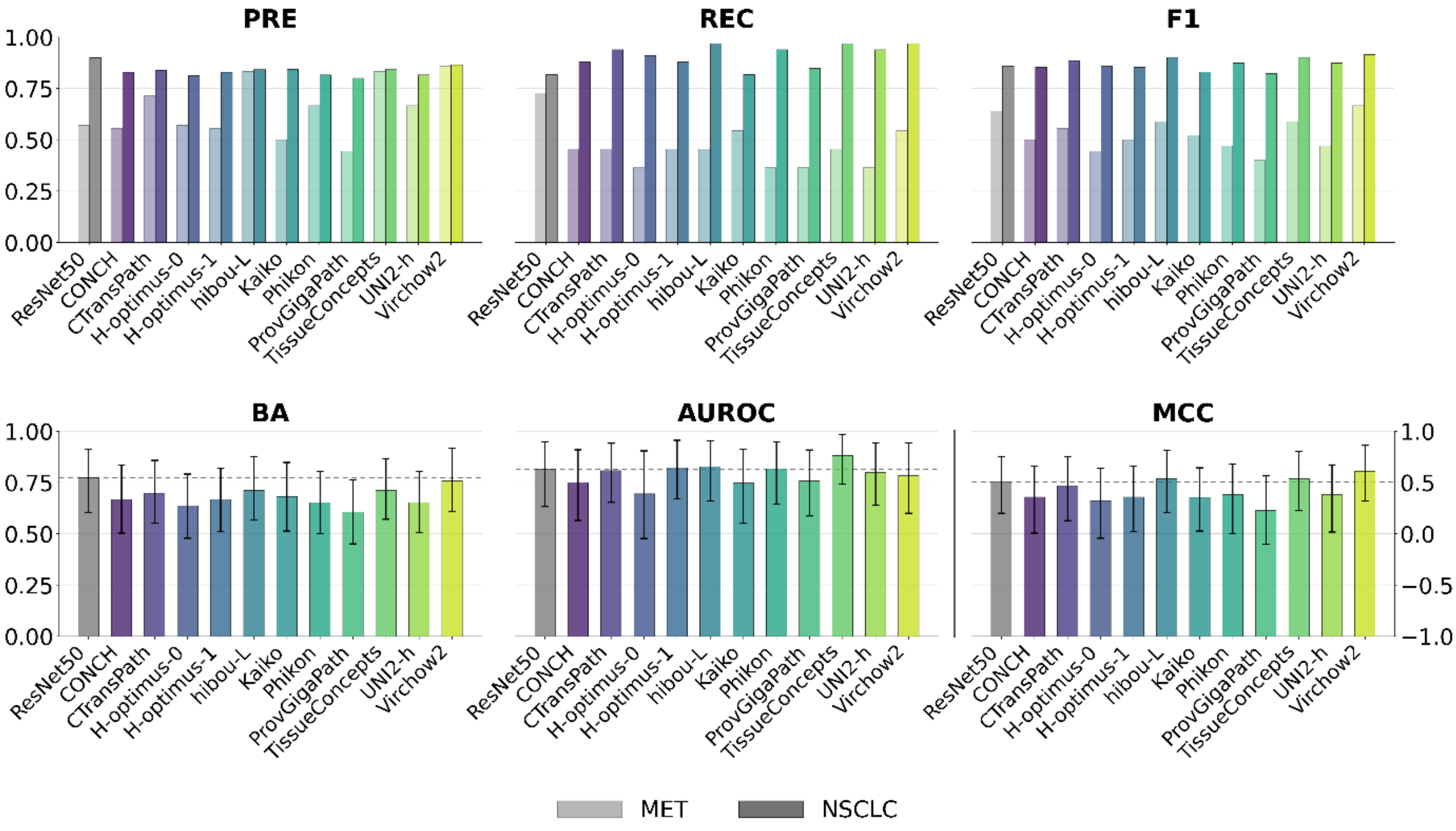


*Figure S4: MLP: Slide-level performance metrics NSCLC vs. MET.*

## 2. Performance metrics plots AC vs. SCC

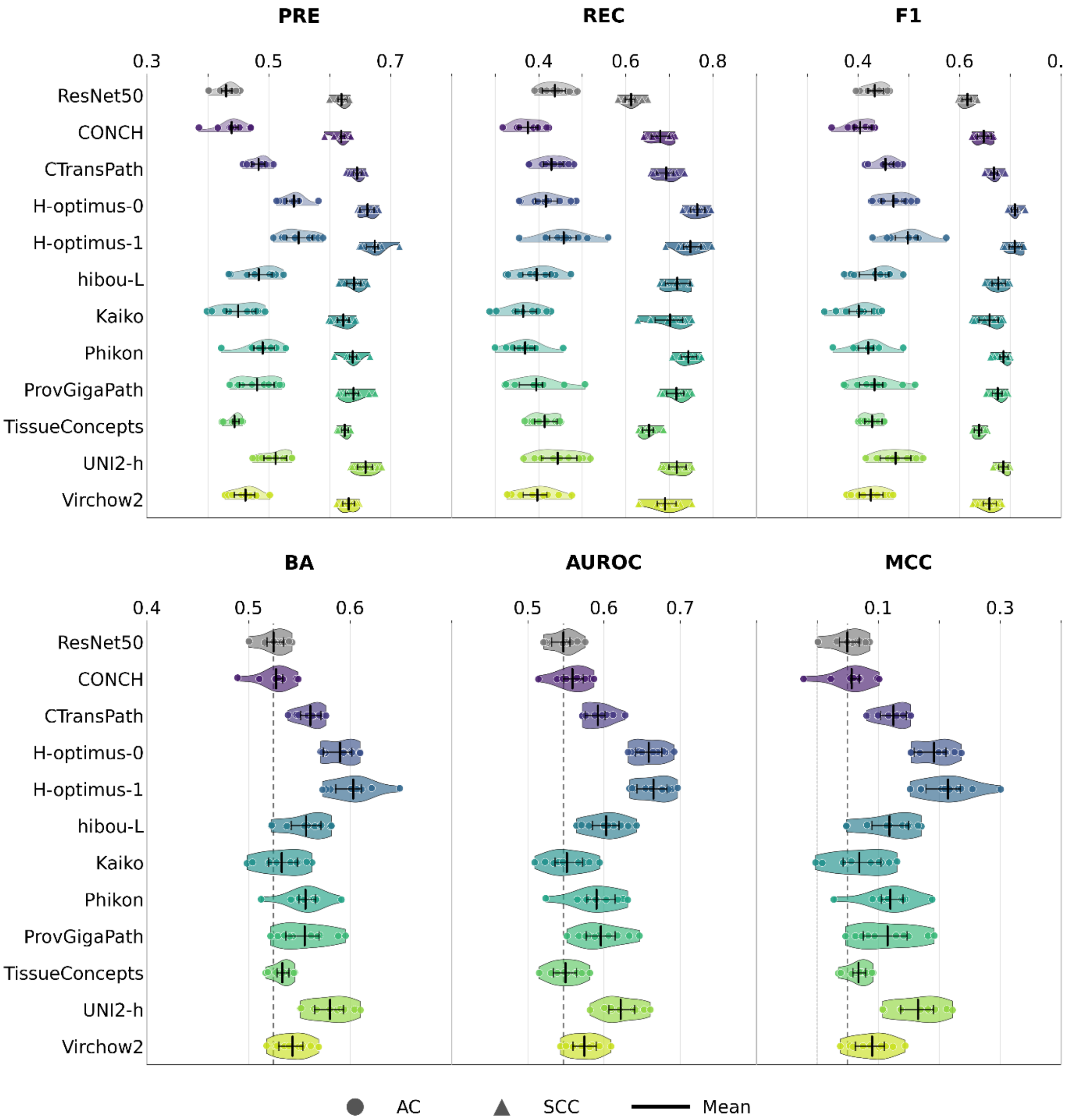


*Figure S5: Logistic regression: Patch-level performance metrics AC vs. SCC.*

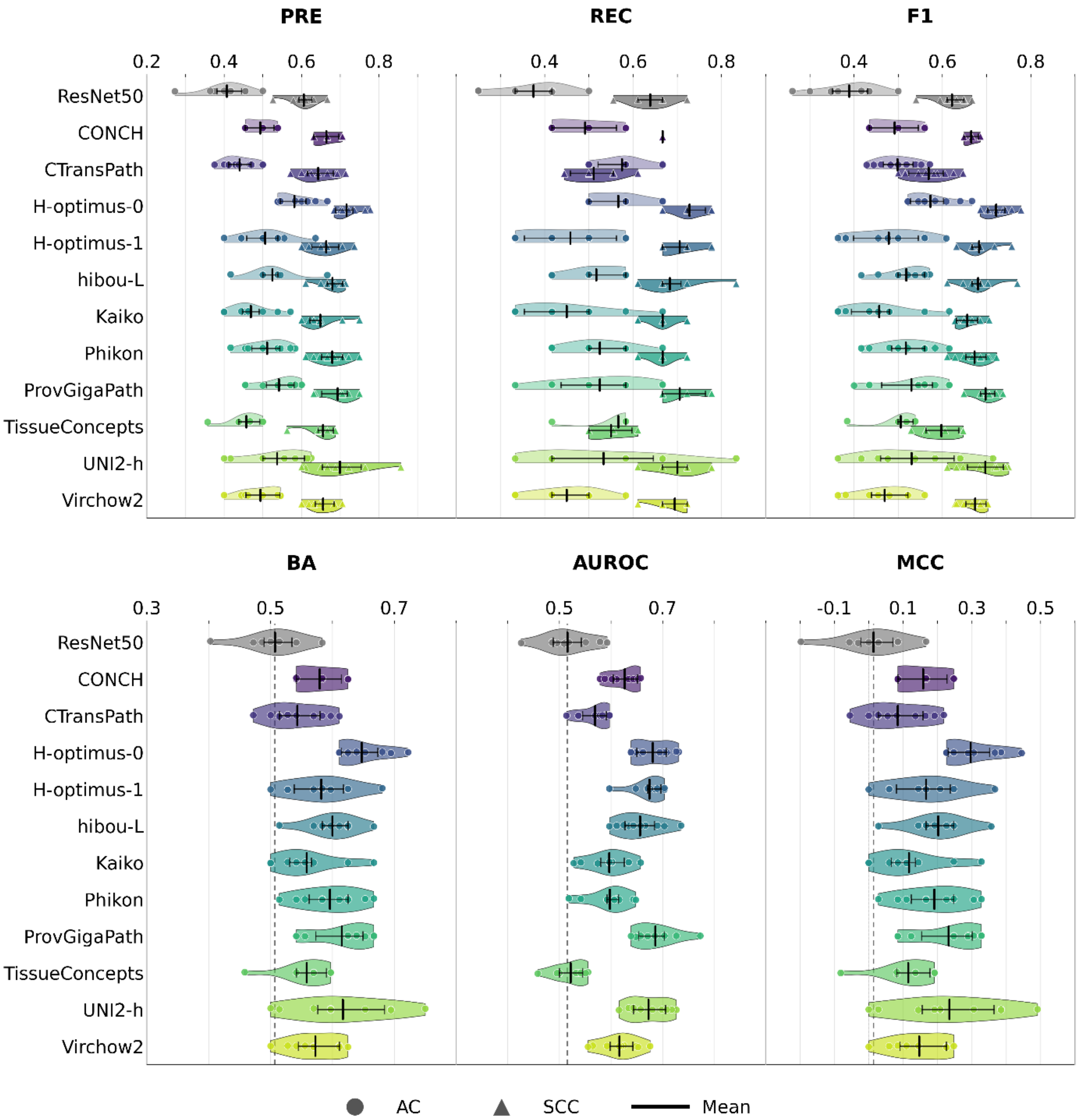


*Figure S6: Logistic regression: Slide-level performance metrics AC vs. SCC.*

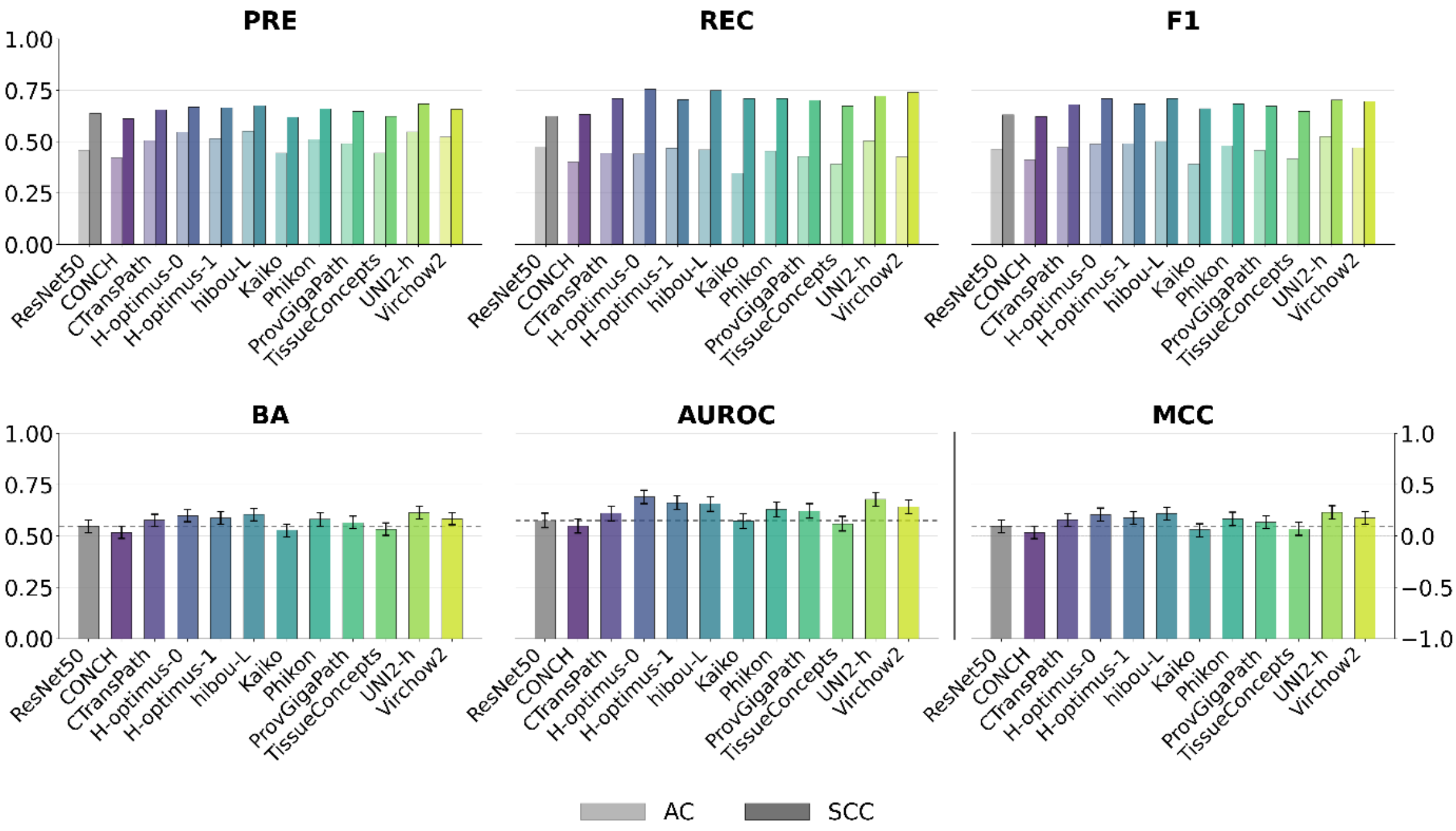

*Figure S7: MLP: Patch-level performance metrics AC vs. SCC.*

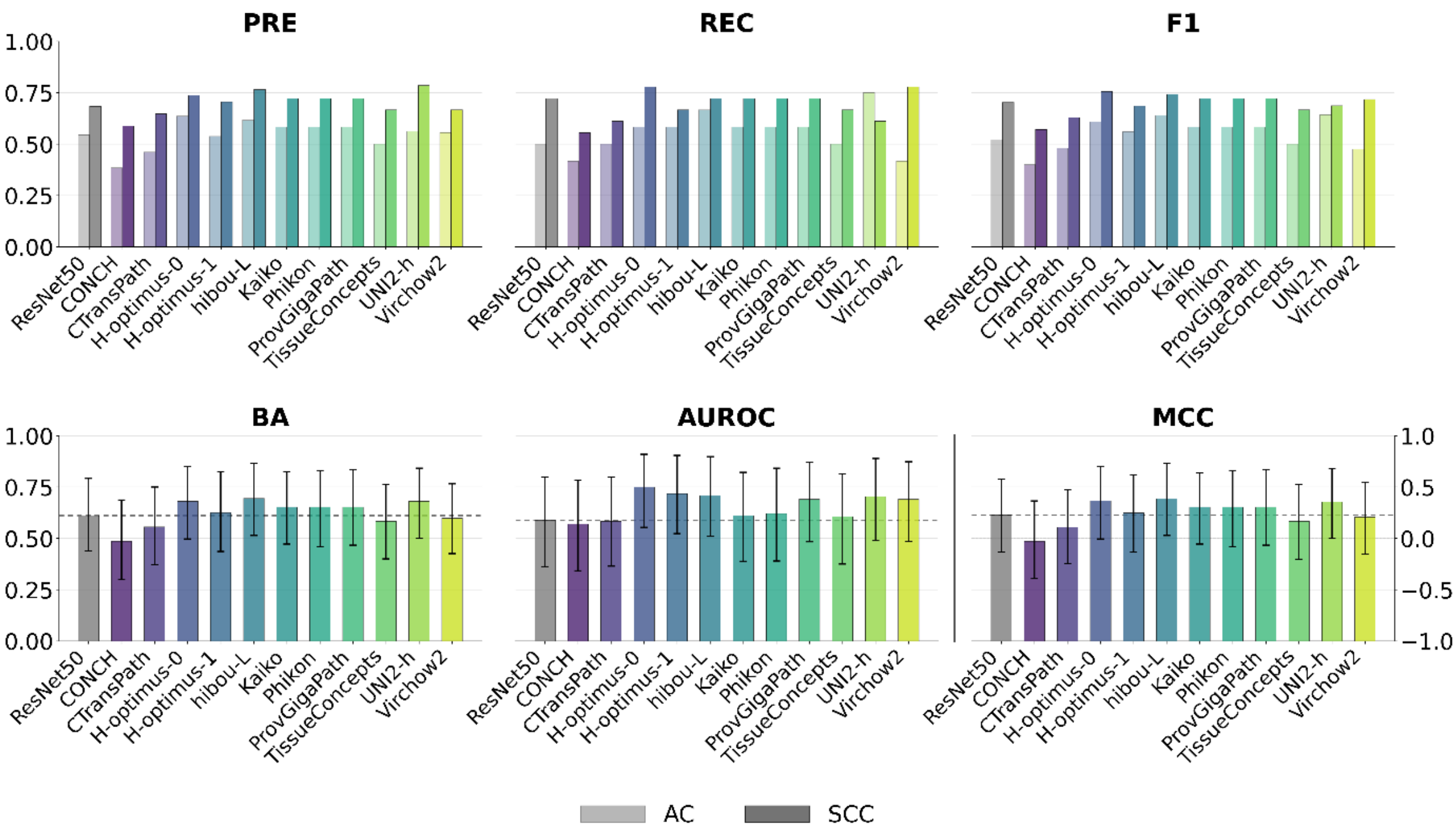

*Figure S8: MLP: Slide-level performance metrics AC vs. SCC.*

## 3. Performance metrics tables NSCLC vs. MET

*Table S1: Logistic regression on CONCH embeddings: Patch-level performance metrics NSCLC vs. MET.*

| Fold | Class | PRE | REC | F1 | BA | AUROC | MCC |
|---|---|---|---|---|---|---|---|
| Fold 1 | MET | 0.6289 | 0.3668 | 0.4634 | 0.6047 | 0.6902 | 0.2402 |
| | NSCLC | 0.6467 | 0.8426 | 0.7318 | | | |
| Fold 2 | MET | 0.6508 | 0.4227 | 0.5125 | 0.6289 | 0.7317 | 0.2855 |
| | NSCLC | 0.6655 | 0.8351 | 0.7407 | | | |
| Fold 3 | MET | 0.5705 | 0.3720 | 0.4503 | 0.5842 | 0.6702 | 0.1863 |
| | NSCLC | 0.6356 | 0.7964 | 0.7070 | | | |
| Fold 4 | MET | 0.6376 | 0.4338 | 0.5164 | 0.6273 | 0.7188 | 0.2780 |
| | NSCLC | 0.6660 | 0.8207 | 0.7353 | | | |
| Fold 5 | MET | 0.6318 | 0.3582 | 0.4572 | 0.6032 | 0.7002 | 0.2391 |
| | NSCLC | 0.6451 | 0.8482 | 0.7329 | | | |
| Fold 6 | MET | 0.6876 | 0.4046 | 0.5095 | 0.6355 | 0.7413 | 0.3099 |
| | NSCLC | 0.6668 | 0.8663 | 0.7536 | | | |
| Fold 7 | MET | 0.6358 | 0.3780 | 0.4741 | 0.6103 | 0.7105 | 0.2514 |
| | NSCLC | 0.6507 | 0.8426 | 0.7343 | | | |
| Fold 8 | MET | 0.6229 | 0.3789 | 0.4712 | 0.6060 | 0.6860 | 0.2399 |
| | NSCLC | 0.6485 | 0.8332 | 0.7294 | | | |
| Fold 9 | MET | 0.6190 | 0.4021 | 0.4875 | 0.6111 | 0.7038 | 0.2461 |
| | NSCLC | 0.6536 | 0.8201 | 0.7274 | | | |
| Fold 10 | MET | 0.6367 | 0.4261 | 0.5106 | 0.6247 | 0.7160 | 0.2737 |
| | NSCLC | 0.6636 | 0.8232 | 0.7349 | | | |
| **Mean ± std** | **MET** | 0.6322 ±0.0290 | 0.3943 ±0.0271 | 0.4853 ±0.0253 | 0.6136 ±0.0155 | 0.7069 ±0.0215 | 0.2550 ±0.0339 |
| | **NSCLC** | 0.6542 ±0.0108 | 0.8329 ±0.0191 | 0.7327 ±0.0116 | | | |

*Table S2: Logistic regression on CONCH embeddings: Slide-level performance metrics NSCLC vs. MET.*

| Fold | Class | PRE | REC | F1 | BA | AUROC | MCC |
|---|---|---|---|---|---|---|---|
| Fold 1 | MET | 0.5714 | 0.3636 | 0.4444 | 0.6364 | 0.7135 | 0.3229 |
| | NSCLC | 0.8108 | 0.9091 | 0.8571 | | | |
| Fold 2 | MET | 0.8333 | 0.4545 | 0.5882 | 0.7121 | 0.8017 | 0.5353 |
| | NSCLC | 0.8421 | 0.9697 | 0.9014 | | | |
| Fold 3 | MET | 0.7143 | 0.4545 | 0.5556 | 0.6970 | 0.7493 | 0.4664 |
| | NSCLC | 0.8378 | 0.9394 | 0.8857 | | | |
| Fold 4 | MET | 0.5000 | 0.4545 | 0.4762 | 0.6515 | 0.7686 | 0.3131 |
| | NSCLC | 0.8235 | 0.8485 | 0.8358 | | | |
| Fold 5 | MET | 0.6667 | 0.3636 | 0.4706 | 0.6515 | 0.7548 | 0.3824 |
| | NSCLC | 0.8158 | 0.9394 | 0.8732 | | | |
| Fold 6 | MET | 0.7143 | 0.4545 | 0.5556 | 0.6970 | 0.7906 | 0.4664 |
| | NSCLC | 0.8378 | 0.9394 | 0.8857 | | | |
| Fold 7 | MET | 0.7143 | 0.4545 | 0.5556 | 0.6970 | 0.7576 | 0.4664 |
| | NSCLC | 0.8378 | 0.9394 | 0.8857 | | | |
| Fold 8 | MET | 0.6667 | 0.5455 | 0.6000 | 0.7273 | 0.7824 | 0.4880 |
| | NSCLC | 0.8571 | 0.9091 | 0.8824 | | | |
| Fold 9 | MET | 0.5556 | 0.4545 | 0.5000 | 0.6667 | 0.7521 | 0.3578 |
| | NSCLC | 0.8286 | 0.8788 | 0.8529 | | | |
| Fold 10 | MET | 0.5556 | 0.4545 | 0.5000 | 0.6667 | 0.7686 | 0.3578 |
| | NSCLC | 0.8286 | 0.8788 | 0.8529 | | | |
| **Mean ± std** | **MET** | 0.6492 ±0.1017 | 0.4455 ±0.0516 | 0.5246 ±0.0532 | 0.6803 ±0.0298 | 0.7639 ±0.0248 | 0.4156 ±0.0776 |
| | **NSCLC** | 0.8320 ±0.0135 | 0.9152 ±0.0373 | 0.8713 ±0.0205 | | | |

*Table S3: Logistic regression on CONCH embeddings: Patient-level performance metrics NSCLC vs. MET.*

| Fold | Class | PRE | REC | F1 | BA | AUROC | MCC |
|---|---|---|---|---|---|---|---|
| Fold 1 | MET | 0.6667 | 0.2500 | 0.3636 | 0.6012 | 0.7321 | 0.2970 |
| | NSCLC | 0.7692 | 0.9524 | 0.8511 | | | |
| Fold 2 | MET | 0.7500 | 0.3750 | 0.5000 | 0.6637 | 0.8333 | 0.4243 |
| | NSCLC | 0.8000 | 0.9524 | 0.8696 | | | |
| Fold 3 | MET | 0.7500 | 0.3750 | 0.5000 | 0.6637 | 0.7679 | 0.4243 |
| | NSCLC | 0.8000 | 0.9524 | 0.8696 | | | |
| Fold 4 | MET | 0.4286 | 0.3750 | 0.4000 | 0.5923 | 0.7679 | 0.1927 |
| | NSCLC | 0.7727 | 0.8095 | 0.7907 | | | |
| Fold 5 | MET | 0.6667 | 0.2500 | 0.3636 | 0.6012 | 0.7440 | 0.2970 |
| | NSCLC | 0.7692 | 0.9524 | 0.8511 | | | |
| Fold 6 | MET | 0.7500 | 0.3750 | 0.5000 | 0.6637 | 0.8274 | 0.4243 |
| | NSCLC | 0.8000 | 0.9524 | 0.8696 | | | |
| Fold 7 | MET | 0.6000 | 0.3750 | 0.4615 | 0.6399 | 0.7738 | 0.3310 |
| | NSCLC | 0.7917 | 0.9048 | 0.8444 | | | |
| Fold 8 | MET | 0.6667 | 0.5000 | 0.5714 | 0.7024 | 0.7798 | 0.4466 |
| | NSCLC | 0.8261 | 0.9048 | 0.8636 | | | |
| Fold 9 | MET | 0.5000 | 0.3750 | 0.4286 | 0.6161 | 0.7619 | 0.2561 |
| | NSCLC | 0.7826 | 0.8571 | 0.8182 | | | |
| Fold 10 | MET | 0.4286 | 0.3750 | 0.4000 | 0.5923 | 0.7857 | 0.1927 |
| | NSCLC | 0.7727 | 0.8095 | 0.7907 | | | |
| **Mean ± std** | **MET** | 0.6207 ±0.1271 | 0.3625 ±0.0710 | 0.4489 ±0.0689 | 0.6336 ±0.0385 | 0.7774 ±0.0321 | 0.3286 ±0.0975 |
| | **NSCLC** | 0.7884 ±0.0185 | 0.9048 ±0.0594 | 0.8418 ±0.0312 | | | |

*Table S4: Logistic regression on CTransPath embeddings: Patch-level performance metrics NSCLC vs. MET.*

| Fold | Class | PRE | REC | F1 | BA | AUROC | MCC |
|---|---|---|---|---|---|---|---|
| Fold 1 | MET | 0.6393 | 0.3548 | 0.4564 | 0.6046 | 0.7102 | 0.2442 |
| | NSCLC | 0.6456 | 0.8545 | 0.7355 | | | |
| Fold 2 | MET | 0.6897 | 0.4622 | 0.5535 | 0.6555 | 0.7680 | 0.3412 |
| | NSCLC | 0.6846 | 0.8488 | 0.7579 | | | |
| Fold 3 | MET | 0.6628 | 0.4373 | 0.5269 | 0.6378 | 0.7322 | 0.3037 |
| | NSCLC | 0.6720 | 0.8382 | 0.7460 | | | |
| Fold 4 | MET | 0.6803 | 0.4278 | 0.5253 | 0.6408 | 0.7530 | 0.3152 |
| | NSCLC | 0.6724 | 0.8538 | 0.7523 | | | |
| Fold 5 | MET | 0.6845 | 0.4064 | 0.5100 | 0.6351 | 0.7344 | 0.3081 |
| | NSCLC | 0.6668 | 0.8638 | 0.7527 | | | |
| Fold 6 | MET | 0.6686 | 0.4055 | 0.5048 | 0.6297 | 0.7307 | 0.2936 |
| | NSCLC | 0.6639 | 0.8538 | 0.7470 | | | |
| Fold 7 | MET | 0.6720 | 0.3960 | 0.4984 | 0.6278 | 0.7323 | 0.2921 |
| | NSCLC | 0.6619 | 0.8595 | 0.7478 | | | |
| Fold 8 | MET | 0.6707 | 0.3763 | 0.4821 | 0.6210 | 0.7180 | 0.2813 |
| | NSCLC | 0.6562 | 0.8657 | 0.7466 | | | |
| Fold 9 | MET | 0.6618 | 0.4287 | 0.5203 | 0.6347 | 0.7318 | 0.2987 |
| | NSCLC | 0.6693 | 0.8407 | 0.7453 | | | |
| Fold 10 | MET | 0.6841 | 0.4261 | 0.5251 | 0.6415 | 0.7469 | 0.3178 |
| | NSCLC | 0.6725 | 0.8570 | 0.7536 | | | |
| **Mean ± std** | **MET** | 0.6714 ±0.0147 | 0.4121 ±0.0312 | 0.5103 ±0.0270 | 0.6329 ±0.0136 | 0.7357 ±0.0167 | 0.2996 ±0.0256 |
| | **NSCLC** | 0.6665 ±0.0106 | 0.8536 ±0.0090 | 0.7485 ±0.0061 | | | |

*Table S5: Logistic regression on CTransPath embeddings: Slide-level performance metrics NSCLC vs. MET.*

| Fold | Class | PRE | REC | F1 | BA | AUROC | MCC |
|---|---|---|---|---|---|---|---|
| Fold 1 | MET | 0.4000 | 0.3636 | 0.3810 | 0.5909 | 0.7603 | 0.1879 |
| | NSCLC | 0.7941 | 0.8182 | 0.8060 | | | |
| Fold 2 | MET | 0.6667 | 0.3636 | 0.4706 | 0.6515 | 0.8512 | 0.3824 |
| | NSCLC | 0.8158 | 0.9394 | 0.8732 | | | |
| Fold 3 | MET | 0.7143 | 0.4545 | 0.5556 | 0.6970 | 0.8457 | 0.4664 |
| | NSCLC | 0.8378 | 0.9394 | 0.8857 | | | |
| Fold 4 | MET | 0.6667 | 0.5455 | 0.6000 | 0.7273 | 0.8540 | 0.4880 |
| | NSCLC | 0.8571 | 0.9091 | 0.8824 | | | |
| Fold 5 | MET | 0.5000 | 0.4545 | 0.4762 | 0.6515 | 0.7961 | 0.3131 |
| | NSCLC | 0.8235 | 0.8485 | 0.8358 | | | |
| Fold 6 | MET | 0.5000 | 0.3636 | 0.4211 | 0.6212 | 0.8595 | 0.2722 |
| | NSCLC | 0.8056 | 0.8788 | 0.8406 | | | |
| Fold 7 | MET | 1.0000 | 0.3636 | 0.5333 | 0.6818 | 0.8512 | 0.5477 |
| | NSCLC | 0.8250 | 1.0000 | 0.9041 | | | |
| Fold 8 | MET | 0.8000 | 0.3636 | 0.5000 | 0.6667 | 0.8347 | 0.4548 |
| | NSCLC | 0.8205 | 0.9697 | 0.8889 | | | |
| Fold 9 | MET | 0.6250 | 0.4545 | 0.5263 | 0.6818 | 0.8292 | 0.4082 |
| | NSCLC | 0.8333 | 0.9091 | 0.8696 | | | |
| Fold 10 | MET | 0.5556 | 0.4545 | 0.5000 | 0.6667 | 0.8320 | 0.3578 |
| | NSCLC | 0.8286 | 0.8788 | 0.8529 | | | |
| **Mean ± std** | **MET** | 0.6428 ±0.1720 | 0.4182 ±0.0636 | 0.4964 ±0.0638 | 0.6636 ±0.0383 | 0.8314 ±0.0309 | 0.3878 ±0.1088 |
| | **NSCLC** | 0.8241 ±0.0174 | 0.9091 ±0.0553 | 0.8639 ±0.0298 | | | |

*Table S6: Logistic regression on CTransPath embeddings: Patient-level performance metrics NSCLC vs. MET.*

| Fold | Class | PRE | REC | F1 | BA | AUROC | MCC |
|---|---|---|---|---|---|---|---|
| Fold 1 | MET | 0.4000 | 0.2500 | 0.3077 | 0.5536 | 0.7500 | 0.1268 |
| | NSCLC | 0.7500 | 0.8571 | 0.8000 | | | |
| Fold 2 | MET | 0.7500 | 0.3750 | 0.5000 | 0.6637 | 0.8988 | 0.4243 |
| | NSCLC | 0.8000 | 0.9524 | 0.8696 | | | |
| Fold 3 | MET | 1.0000 | 0.3750 | 0.5455 | 0.6875 | 0.8750 | 0.5503 |
| | NSCLC | 0.8077 | 1.0000 | 0.8936 | | | |
| Fold 4 | MET | 0.8000 | 0.5000 | 0.6154 | 0.7262 | 0.8750 | 0.5353 |
| | NSCLC | 0.8333 | 0.9524 | 0.8889 | | | |
| Fold 5 | MET | 0.6000 | 0.3750 | 0.4615 | 0.6399 | 0.8214 | 0.3310 |
| | NSCLC | 0.7917 | 0.9048 | 0.8444 | | | |
| Fold 6 | MET | 0.5000 | 0.2500 | 0.3333 | 0.5774 | 0.8750 | 0.2006 |
| | NSCLC | 0.7600 | 0.9048 | 0.8261 | | | |
| Fold 7 | MET | 1.0000 | 0.2500 | 0.4000 | 0.6250 | 0.8690 | 0.4410 |
| | NSCLC | 0.7778 | 1.0000 | 0.8750 | | | |
| Fold 8 | MET | 1.0000 | 0.2500 | 0.4000 | 0.6250 | 0.8452 | 0.4410 |
| | NSCLC | 0.7778 | 1.0000 | 0.8750 | | | |
| Fold 9 | MET | 1.0000 | 0.3750 | 0.5455 | 0.6875 | 0.8571 | 0.5503 |
| | NSCLC | 0.8077 | 1.0000 | 0.8936 | | | |
| Fold 10 | MET | 0.7500 | 0.3750 | 0.5000 | 0.6637 | 0.8690 | 0.4243 |
| | NSCLC | 0.8000 | 0.9524 | 0.8696 | | | |
| **Mean ± std** | **MET** | 0.7800 ±0.2239 | 0.3375 ±0.0844 | 0.4609 ±0.0991 | 0.6449 ±0.0523 | 0.8536 ±0.0418 | 0.4025 ±0.1442 |
| | **NSCLC** | 0.7906 ±0.0247 | 0.9524 ±0.0502 | 0.8636 ±0.0309 | | | |

*Table S7: Logistic regression on H-optimus-0 embeddings: Patch-level performance metrics NSCLC vs. MET.*

| Fold | Class | PRE | REC | F1 | BA | AUROC | MCC |
|---|---|---|---|---|---|---|---|
| Fold 1 | MET | 0.6192 | 0.3436 | 0.4420 | 0.5950 | 0.6787 | 0.2217 |
| | NSCLC | 0.6395 | 0.8463 | 0.7285 | | | |
| Fold 2 | MET | 0.6303 | 0.3823 | 0.4759 | 0.6096 | 0.7036 | 0.2483 |
| | NSCLC | 0.6508 | 0.8370 | 0.7322 | | | |
| Fold 3 | MET | 0.6535 | 0.3402 | 0.4475 | 0.6045 | 0.6919 | 0.2495 |
| | NSCLC | 0.6443 | 0.8688 | 0.7399 | | | |
| Fold 4 | MET | 0.6658 | 0.4313 | 0.5235 | 0.6369 | 0.7219 | 0.3036 |
| | NSCLC | 0.6708 | 0.8426 | 0.7470 | | | |
| Fold 5 | MET | 0.6731 | 0.3574 | 0.4669 | 0.6156 | 0.6978 | 0.2740 |
| | NSCLC | 0.6516 | 0.8738 | 0.7465 | | | |
| Fold 6 | MET | 0.6683 | 0.3514 | 0.4606 | 0.6123 | 0.6910 | 0.2671 |
| | NSCLC | 0.6493 | 0.8732 | 0.7448 | | | |
| Fold 7 | MET | 0.6404 | 0.3488 | 0.4516 | 0.6032 | 0.6845 | 0.2424 |
| | NSCLC | 0.6443 | 0.8576 | 0.7358 | | | |
| Fold 8 | MET | 0.6838 | 0.3771 | 0.4862 | 0.6252 | 0.6964 | 0.2927 |
| | NSCLC | 0.6585 | 0.8732 | 0.7508 | | | |
| Fold 9 | MET | 0.6257 | 0.4021 | 0.4895 | 0.6136 | 0.6820 | 0.2525 |
| | NSCLC | 0.6549 | 0.8251 | 0.7302 | | | |
| Fold 10 | MET | 0.6520 | 0.3832 | 0.4827 | 0.6173 | 0.7052 | 0.2683 |
| | NSCLC | 0.6550 | 0.8513 | 0.7404 | | | |
| **Mean ± std** | **MET** | 0.6512 ±0.0218 | 0.3717 ±0.0292 | 0.4726 ±0.0244 | 0.6133 ±0.0118 | 0.6953 ±0.0128 | 0.2620 ±0.0243 |
| | **NSCLC** | 0.6519 ±0.0088 | 0.8549 ±0.0172 | 0.7396 ±0.0077 | | | |

*Table S8: Logistic regression on H-optimus-0 embeddings: Slide-level performance metrics NSCLC vs. MET.*

| Fold | Class | PRE | REC | F1 | BA | AUROC | MCC |
|---|---|---|---|---|---|---|---|
| Fold 1 | MET | 0.6667 | 0.3636 | 0.4706 | 0.6515 | 0.7245 | 0.3824 |
| | NSCLC | 0.8158 | 0.9394 | 0.8732 | | | |
| Fold 2 | MET | 0.4286 | 0.2727 | 0.3333 | 0.5758 | 0.7273 | 0.1794 |
| | NSCLC | 0.7838 | 0.8788 | 0.8286 | | | |
| Fold 3 | MET | 0.8000 | 0.3636 | 0.5000 | 0.6667 | 0.8072 | 0.4548 |
| | NSCLC | 0.8205 | 0.9697 | 0.8889 | | | |
| Fold 4 | MET | 0.8750 | 0.6364 | 0.7368 | 0.8030 | 0.7879 | 0.6804 |
| | NSCLC | 0.8889 | 0.9697 | 0.9275 | | | |
| Fold 5 | MET | 0.8333 | 0.4545 | 0.5882 | 0.7121 | 0.7769 | 0.5353 |
| | NSCLC | 0.8421 | 0.9697 | 0.9014 | | | |
| Fold 6 | MET | 0.7143 | 0.4545 | 0.5556 | 0.6970 | 0.8292 | 0.4664 |
| | NSCLC | 0.8378 | 0.9394 | 0.8857 | | | |
| Fold 7 | MET | 0.6667 | 0.3636 | 0.4706 | 0.6515 | 0.7851 | 0.3824 |
| | NSCLC | 0.8158 | 0.9394 | 0.8732 | | | |
| Fold 8 | MET | 0.7500 | 0.5455 | 0.6316 | 0.7424 | 0.7631 | 0.5443 |
| | NSCLC | 0.8611 | 0.9394 | 0.8986 | | | |
| Fold 9 | MET | 0.7143 | 0.4545 | 0.5556 | 0.6970 | 0.7548 | 0.4664 |
| | NSCLC | 0.8378 | 0.9394 | 0.8857 | | | |
| Fold 10 | MET | 0.6667 | 0.3636 | 0.4706 | 0.6515 | 0.7934 | 0.3824 |
| | NSCLC | 0.8158 | 0.9394 | 0.8732 | | | |
| **Mean ± std** | **MET** | 0.7115 ±0.1234 | 0.4273 ±0.1054 | 0.5313 ±0.1095 | 0.6848 ±0.0614 | 0.7749 ±0.0333 | 0.4474 ±0.1320 |
| | **NSCLC** | 0.8319 ±0.0289 | 0.9424 ±0.0265 | 0.8836 ±0.0255 | | | |

*Table S9: Logistic regression on H-optimus-0 embeddings: Patient-level performance metrics NSCLC vs. MET.*

| Fold | Class | PRE | REC | F1 | BA | AUROC | MCC |
|---|---|---|---|---|---|---|---|
| Fold 1 | MET | 0.6667 | 0.2500 | 0.3636 | 0.6012 | 0.7262 | 0.2970 |
| | NSCLC | 0.7692 | 0.9524 | 0.8511 | | | |
| Fold 2 | MET | 1.0000 | 0.2500 | 0.4000 | 0.6250 | 0.7917 | 0.4410 |
| | NSCLC | 0.7778 | 1.0000 | 0.8750 | | | |
| Fold 3 | MET | 1.0000 | 0.2500 | 0.4000 | 0.6250 | 0.8155 | 0.4410 |
| | NSCLC | 0.7778 | 1.0000 | 0.8750 | | | |
| Fold 4 | MET | 1.0000 | 0.6250 | 0.7692 | 0.8125 | 0.8095 | 0.7395 |
| | NSCLC | 0.8750 | 1.0000 | 0.9333 | | | |
| Fold 5 | MET | 1.0000 | 0.3750 | 0.5455 | 0.6875 | 0.7798 | 0.5503 |
| | NSCLC | 0.8077 | 1.0000 | 0.8936 | | | |
| Fold 6 | MET | 0.7500 | 0.3750 | 0.5000 | 0.6637 | 0.8155 | 0.4243 |
| | NSCLC | 0.8000 | 0.9524 | 0.8696 | | | |
| Fold 7 | MET | 1.0000 | 0.2500 | 0.4000 | 0.6250 | 0.7619 | 0.4410 |
| | NSCLC | 0.7778 | 1.0000 | 0.8750 | | | |
| Fold 8 | MET | 1.0000 | 0.5000 | 0.6667 | 0.7500 | 0.7738 | 0.6481 |
| | NSCLC | 0.8400 | 1.0000 | 0.9130 | | | |
| Fold 9 | MET | 1.0000 | 0.3750 | 0.5455 | 0.6875 | 0.7679 | 0.5503 |
| | NSCLC | 0.8077 | 1.0000 | 0.8936 | | | |
| Fold 10 | MET | 1.0000 | 0.2500 | 0.4000 | 0.6250 | 0.8095 | 0.4410 |
| | NSCLC | 0.7778 | 1.0000 | 0.8750 | | | |
| **Mean ± std** | **MET** | 0.9417 ±0.1245 | 0.3500 ±0.1291 | 0.4990 ±0.1346 | 0.6702 ±0.0668 | 0.7851 ±0.0290 | 0.4973 ±0.1270 |
| | **NSCLC** | 0.8011 ±0.0338 | 0.9905 ±0.0201 | 0.8854 ±0.0237 | | | |

*Table S10: Logistic regression on H-optimus-1 embeddings: Patch-level performance metrics NSCLC vs. MET.*

| Fold | Class | PRE | REC | F1 | BA | AUROC | MCC |
|---|---|---|---|---|---|---|---|
| Fold 1 | MET | 0.6054 | 0.3282 | 0.4256 | 0.5863 | 0.6945 | 0.2031 |
| | NSCLC | 0.6336 | 0.8445 | 0.7240 | | | |
| Fold 2 | MET | 0.6334 | 0.3814 | 0.4761 | 0.6105 | 0.7223 | 0.2507 |
| | NSCLC | 0.6512 | 0.8395 | 0.7334 | | | |
| Fold 3 | MET | 0.5938 | 0.3780 | 0.4619 | 0.5950 | 0.6922 | 0.2118 |
| | NSCLC | 0.6423 | 0.8120 | 0.7172 | | | |
| Fold 4 | MET | 0.6327 | 0.3952 | 0.4865 | 0.6142 | 0.7250 | 0.2562 |
| | NSCLC | 0.6546 | 0.8332 | 0.7332 | | | |
| Fold 5 | MET | 0.6746 | 0.3634 | 0.4724 | 0.6180 | 0.7266 | 0.2782 |
| | NSCLC | 0.6534 | 0.8726 | 0.7473 | | | |
| Fold 6 | MET | 0.6205 | 0.3119 | 0.4151 | 0.5866 | 0.6944 | 0.2094 |
| | NSCLC | 0.6326 | 0.8613 | 0.7294 | | | |
| Fold 7 | MET | 0.6335 | 0.3505 | 0.4513 | 0.6016 | 0.7057 | 0.2372 |
| | NSCLC | 0.6436 | 0.8526 | 0.7335 | | | |
| Fold 8 | MET | 0.6098 | 0.3101 | 0.4112 | 0.5829 | 0.7011 | 0.1996 |
| | NSCLC | 0.6305 | 0.8557 | 0.7260 | | | |
| Fold 9 | MET | 0.6043 | 0.4107 | 0.4890 | 0.6076 | 0.7073 | 0.2350 |
| | NSCLC | 0.6525 | 0.8045 | 0.7206 | | | |
| Fold 10 | MET | 0.6397 | 0.3875 | 0.4826 | 0.6144 | 0.7254 | 0.2592 |
| | NSCLC | 0.6539 | 0.8413 | 0.7359 | | | |
| **Mean ± std** | **MET** | 0.6248 ±0.0234 | 0.3617 ±0.0353 | 0.4572 ±0.0299 | 0.6017 ±0.0132 | 0.7094 ±0.0141 | 0.2340 ±0.0271 |
| | **NSCLC** | 0.6448 ±0.0097 | 0.8417 ±0.0211 | 0.7300 ±0.0086 | | | |

*Table S11: Logistic regression on H-optimus-1 embeddings: Slide-level performance metrics NSCLC vs. MET.*

| Fold | Class | PRE | REC | F1 | BA | AUROC | MCC |
|---|---|---|---|---|---|---|---|
| Fold 1 | MET | 0.5000 | 0.3636 | 0.4211 | 0.6212 | 0.7080 | 0.2722 |
| | NSCLC | 0.8056 | 0.8788 | 0.8406 | | | |
| Fold 2 | MET | 0.6250 | 0.4545 | 0.5263 | 0.6818 | 0.8292 | 0.4082 |
| | NSCLC | 0.8333 | 0.9091 | 0.8696 | | | |
| Fold 3 | MET | 1.0000 | 0.4545 | 0.6250 | 0.7273 | 0.8044 | 0.6202 |
| | NSCLC | 0.8462 | 1.0000 | 0.9167 | | | |
| Fold 4 | MET | 0.7143 | 0.4545 | 0.5556 | 0.6970 | 0.8209 | 0.4664 |
| | NSCLC | 0.8378 | 0.9394 | 0.8857 | | | |
| Fold 5 | MET | 0.6667 | 0.3636 | 0.4706 | 0.6515 | 0.8402 | 0.3824 |
| | NSCLC | 0.8158 | 0.9394 | 0.8732 | | | |
| Fold 6 | MET | 0.7143 | 0.4545 | 0.5556 | 0.6970 | 0.8264 | 0.4664 |
| | NSCLC | 0.8378 | 0.9394 | 0.8857 | | | |
| Fold 7 | MET | 0.7143 | 0.4545 | 0.5556 | 0.6970 | 0.8264 | 0.4664 |
| | NSCLC | 0.8378 | 0.9394 | 0.8857 | | | |
| Fold 8 | MET | 0.8333 | 0.4545 | 0.5882 | 0.7121 | 0.7741 | 0.5353 |
| | NSCLC | 0.8421 | 0.9697 | 0.9014 | | | |
| Fold 9 | MET | 0.7143 | 0.4545 | 0.5556 | 0.6970 | 0.8237 | 0.4664 |
| | NSCLC | 0.8378 | 0.9394 | 0.8857 | | | |
| Fold 10 | MET | 0.7143 | 0.4545 | 0.5556 | 0.6970 | 0.8347 | 0.4664 |
| | NSCLC | 0.8378 | 0.9394 | 0.8857 | | | |
| **Mean ± std** | **MET** | 0.7196 ±0.1298 | 0.4364 ±0.0383 | 0.5409 ±0.0576 | 0.6879 ±0.0305 | 0.8088 ±0.0401 | 0.4550 ±0.0915 |
| | **NSCLC** | 0.8332 ±0.0126 | 0.9394 ±0.0319 | 0.8830 ±0.0200 | | | |

*Table S12: Logistic regression on H-optimus-1 embeddings: Patient-level performance metrics NSCLC vs. MET.*

| Fold | Class | PRE | REC | F1 | BA | AUROC | MCC |
|---|---|---|---|---|---|---|---|
| Fold 1 | MET | 0.6667 | 0.2500 | 0.3636 | 0.6012 | 0.7083 | 0.2970 |
| | NSCLC | 0.7692 | 0.9524 | 0.8511 | | | |
| Fold 2 | MET | 0.8000 | 0.5000 | 0.6154 | 0.7262 | 0.8571 | 0.5353 |
| | NSCLC | 0.8333 | 0.9524 | 0.8889 | | | |
| Fold 3 | MET | 1.0000 | 0.3750 | 0.5455 | 0.6875 | 0.8095 | 0.5503 |
| | NSCLC | 0.8077 | 1.0000 | 0.8936 | | | |
| Fold 4 | MET | 1.0000 | 0.3750 | 0.5455 | 0.6875 | 0.8571 | 0.5503 |
| | NSCLC | 0.8077 | 1.0000 | 0.8936 | | | |
| Fold 5 | MET | 1.0000 | 0.2500 | 0.4000 | 0.6250 | 0.8631 | 0.4410 |
| | NSCLC | 0.7778 | 1.0000 | 0.8750 | | | |
| Fold 6 | MET | 1.0000 | 0.2500 | 0.4000 | 0.6250 | 0.8274 | 0.4410 |
| | NSCLC | 0.7778 | 1.0000 | 0.8750 | | | |
| Fold 7 | MET | 1.0000 | 0.3750 | 0.5455 | 0.6875 | 0.8274 | 0.5503 |
| | NSCLC | 0.8077 | 1.0000 | 0.8936 | | | |
| Fold 8 | MET | 1.0000 | 0.3750 | 0.5455 | 0.6875 | 0.7679 | 0.5503 |
| | NSCLC | 0.8077 | 1.0000 | 0.8936 | | | |
| Fold 9 | MET | 1.0000 | 0.3750 | 0.5455 | 0.6875 | 0.8393 | 0.5503 |
| | NSCLC | 0.8077 | 1.0000 | 0.8936 | | | |
| Fold 10 | MET | 1.0000 | 0.3750 | 0.5455 | 0.6875 | 0.8571 | 0.5503 |
| | NSCLC | 0.8077 | 1.0000 | 0.8936 | | | |
| **Mean ± std** | **MET** | 0.9467 ±0.1167 | 0.3500 ±0.0791 | 0.5052 ±0.0844 | 0.6702 ±0.0391 | 0.8214 ±0.0492 | 0.5016 ±0.0847 |
| | **NSCLC** | 0.8004 ±0.0194 | 0.9905 ±0.0201 | 0.8852 ±0.0142 | | | |

*Table S13: Logistic regression on hibou-L embeddings: Patch-level performance metrics NSCLC vs. MET*

| Fold | Class | PRE | REC | F1 | BA | AUROC | MCC |
|---|---|---|---|---|---|---|---|
| Fold 1 | MET | 0.6631 | 0.3737 | 0.4780 | 0.6178 | 0.7252 | 0.2735 |
| | NSCLC | 0.6543 | 0.8620 | 0.7439 | | | |
| Fold 2 | MET | 0.7160 | 0.5026 | 0.5906 | 0.6788 | 0.7900 | 0.3870 |
| | NSCLC | 0.7028 | 0.8551 | 0.7715 | | | |
| Fold 3 | MET | 0.6844 | 0.4527 | 0.5450 | 0.6505 | 0.7442 | 0.3315 |
| | NSCLC | 0.6807 | 0.8482 | 0.7553 | | | |
| Fold 4 | MET | 0.7069 | 0.4682 | 0.5633 | 0.6635 | 0.7694 | 0.3601 |
| | NSCLC | 0.6896 | 0.8588 | 0.7650 | | | |
| Fold 5 | MET | 0.7430 | 0.4545 | 0.5640 | 0.6701 | 0.7808 | 0.3841 |
| | NSCLC | 0.6907 | 0.8857 | 0.7761 | | | |
| Fold 6 | MET | 0.7149 | 0.4330 | 0.5393 | 0.6537 | 0.7631 | 0.3483 |
| | NSCLC | 0.6796 | 0.8745 | 0.7648 | | | |
| Fold 7 | MET | 0.6831 | 0.3926 | 0.4986 | 0.6301 | 0.7570 | 0.3000 |
| | NSCLC | 0.6627 | 0.8676 | 0.7514 | | | |
| Fold 8 | MET | 0.6662 | 0.4201 | 0.5153 | 0.6335 | 0.7350 | 0.2986 |
| | NSCLC | 0.6677 | 0.8470 | 0.7467 | | | |
| Fold 9 | MET | 0.6709 | 0.4588 | 0.5449 | 0.6476 | 0.7515 | 0.3218 |
| | NSCLC | 0.6800 | 0.8364 | 0.7501 | | | |
| Fold 10 | MET | 0.7081 | 0.4751 | 0.5686 | 0.6663 | 0.7745 | 0.3648 |
| | NSCLC | 0.6920 | 0.8576 | 0.7660 | | | |
| **Mean ± std** | **MET** | 0.6957 ±0.0261 | 0.4431 ±0.0389 | 0.5408 ±0.0345 | 0.6512 ±0.0194 | 0.7591 ±0.0206 | 0.3370 ±0.0384 |
| | **NSCLC** | 0.6800 ±0.0148 | 0.8593 ±0.0142 | 0.7591 ±0.0110 | | | |

*Table S14: Logistic regression on hibou-L embeddings: Slide-level performance metrics NSCLC vs. MET.*

| Fold | Class | PRE | REC | F1 | BA | AUROC | MCC |
|---|---|---|---|---|---|---|---|
| Fold 1 | MET | 0.5556 | 0.4545 | 0.5000 | 0.6667 | 0.7466 | 0.3578 |
| | NSCLC | 0.8286 | 0.8788 | 0.8529 | | | |
| Fold 2 | MET | 0.6667 | 0.5455 | 0.6000 | 0.7273 | 0.8733 | 0.4880 |
| | NSCLC | 0.8571 | 0.9091 | 0.8824 | | | |
| Fold 3 | MET | 0.7143 | 0.4545 | 0.5556 | 0.6970 | 0.8347 | 0.4664 |
| | NSCLC | 0.8378 | 0.9394 | 0.8857 | | | |
| Fold 4 | MET | 0.7143 | 0.4545 | 0.5556 | 0.6970 | 0.8567 | 0.4664 |
| | NSCLC | 0.8378 | 0.9394 | 0.8857 | | | |
| Fold 5 | MET | 0.7143 | 0.4545 | 0.5556 | 0.6970 | 0.8678 | 0.4664 |
| | NSCLC | 0.8378 | 0.9394 | 0.8857 | | | |
| Fold 6 | MET | 0.7143 | 0.4545 | 0.5556 | 0.6970 | 0.8926 | 0.4664 |
| | NSCLC | 0.8378 | 0.9394 | 0.8857 | | | |
| Fold 7 | MET | 0.6667 | 0.3636 | 0.4706 | 0.6515 | 0.8347 | 0.3824 |
| | NSCLC | 0.8158 | 0.9394 | 0.8732 | | | |
| Fold 8 | MET | 0.6667 | 0.5455 | 0.6000 | 0.7273 | 0.8209 | 0.4880 |
| | NSCLC | 0.8571 | 0.9091 | 0.8824 | | | |
| Fold 9 | MET | 0.6250 | 0.4545 | 0.5263 | 0.6818 | 0.8264 | 0.4082 |
| | NSCLC | 0.8333 | 0.9091 | 0.8696 | | | |
| Fold 10 | MET | 0.6250 | 0.4545 | 0.5263 | 0.6818 | 0.8650 | 0.4082 |
| | NSCLC | 0.8333 | 0.9091 | 0.8696 | | | |
| **Mean ± std** | **MET** | 0.6663 ±0.0526 | 0.4636 ±0.0516 | 0.5445 ±0.0405 | 0.6924 ±0.0237 | 0.8419 ±0.0406 | 0.4398 ±0.0465 |
| | **NSCLC** | 0.8377 ±0.0123 | 0.9212 ±0.0212 | 0.8773 ±0.0109 | | | |

*Table S15: Logistic regression on hibou-L embeddings: Patient-level performance metrics NSCLC vs. MET.*

| Fold | Class | PRE | REC | F1 | BA | AUROC | MCC |
|---|---|---|---|---|---|---|---|
| Fold 1 | MET | 0.7500 | 0.3750 | 0.5000 | 0.6637 | 0.7321 | 0.4243 |
| | NSCLC | 0.8000 | 0.9524 | 0.8696 | | | |
| Fold 2 | MET | 1.0000 | 0.6250 | 0.7692 | 0.8125 | 0.9167 | 0.7395 |
| | NSCLC | 0.8750 | 1.0000 | 0.9333 | | | |
| Fold 3 | MET | 1.0000 | 0.3750 | 0.5455 | 0.6875 | 0.8690 | 0.5503 |
| | NSCLC | 0.8077 | 1.0000 | 0.8936 | | | |
| Fold 4 | MET | 1.0000 | 0.3750 | 0.5455 | 0.6875 | 0.8988 | 0.5503 |
| | NSCLC | 0.8077 | 1.0000 | 0.8936 | | | |
| Fold 5 | MET | 1.0000 | 0.3750 | 0.5455 | 0.6875 | 0.9167 | 0.5503 |
| | NSCLC | 0.8077 | 1.0000 | 0.8936 | | | |
| Fold 6 | MET | 1.0000 | 0.3750 | 0.5455 | 0.6875 | 0.9167 | 0.5503 |
| | NSCLC | 0.8077 | 1.0000 | 0.8936 | | | |
| Fold 7 | MET | 1.0000 | 0.2500 | 0.4000 | 0.6250 | 0.8571 | 0.4410 |
| | NSCLC | 0.7778 | 1.0000 | 0.8750 | | | |
| Fold 8 | MET | 1.0000 | 0.5000 | 0.6667 | 0.7500 | 0.8274 | 0.6481 |
| | NSCLC | 0.8400 | 1.0000 | 0.9130 | | | |
| Fold 9 | MET | 1.0000 | 0.3750 | 0.5455 | 0.6875 | 0.8512 | 0.5503 |
| | NSCLC | 0.8077 | 1.0000 | 0.8936 | | | |
| Fold 10 | MET | 1.0000 | 0.3750 | 0.5455 | 0.6875 | 0.8988 | 0.5503 |
| | NSCLC | 0.8077 | 1.0000 | 0.8936 | | | |
| **Mean ± std** | **MET** | 0.9750 ±0.0791 | 0.4000 ±0.0986 | 0.5609 ±0.0975 | 0.6976 ±0.0505 | 0.8685 ±0.0573 | 0.5555 ±0.0900 |
| | **NSCLC** | 0.8139 ±0.0261 | 0.9952 ±0.0151 | 0.8953 ±0.0178 | | | |

*Table S16: Logistic regression on Kaiko embeddings: Patch-level performance metrics NSCLC vs. MET.*

| Fold | Class | PRE | REC | F1 | BA | AUROC | MCC |
|---|---|---|---|---|---|---|---|
| Fold 1 | MET | 0.5957 | 0.3582 | 0.4474 | 0.5907 | 0.6756 | 0.2061 |
| | NSCLC | 0.6383 | 0.8232 | 0.7190 | | | |
| Fold 2 | MET | 0.6137 | 0.4381 | 0.5113 | 0.6188 | 0.7131 | 0.2559 |
| | NSCLC | 0.6618 | 0.7995 | 0.7242 | | | |
| Fold 3 | MET | 0.6284 | 0.4184 | 0.5023 | 0.6192 | 0.6944 | 0.2622 |
| | NSCLC | 0.6598 | 0.8201 | 0.7313 | | | |
| Fold 4 | MET | 0.6097 | 0.4321 | 0.5058 | 0.6155 | 0.7074 | 0.2493 |
| | NSCLC | 0.6593 | 0.7989 | 0.7224 | | | |
| Fold 5 | MET | 0.6561 | 0.4278 | 0.5179 | 0.6324 | 0.7176 | 0.2930 |
| | NSCLC | 0.6680 | 0.8370 | 0.7430 | | | |
| Fold 6 | MET | 0.6142 | 0.3488 | 0.4449 | 0.5948 | 0.6603 | 0.2194 |
| | NSCLC | 0.6397 | 0.8407 | 0.7266 | | | |
| Fold 7 | MET | 0.6126 | 0.4021 | 0.4855 | 0.6086 | 0.7004 | 0.2398 |
| | NSCLC | 0.6522 | 0.8151 | 0.7246 | | | |
| Fold 8 | MET | 0.6461 | 0.3875 | 0.4844 | 0.6166 | 0.7018 | 0.2650 |
| | NSCLC | 0.6551 | 0.8457 | 0.7383 | | | |
| Fold 9 | MET | 0.5918 | 0.4210 | 0.4920 | 0.6049 | 0.6811 | 0.2262 |
| | NSCLC | 0.6520 | 0.7889 | 0.7140 | | | |
| Fold 10 | MET | 0.6027 | 0.4158 | 0.4921 | 0.6083 | 0.6992 | 0.2355 |
| | NSCLC | 0.6534 | 0.8007 | 0.7196 | | | |
| **Mean ± std** | **MET** | 0.6171 ±0.0208 | 0.4050 ±0.0309 | 0.4884 ±0.0247 | 0.6110 ±0.0123 | 0.6951 ±0.0178 | 0.2452 ±0.0253 |
| | **NSCLC** | 0.6540 ±0.0093 | 0.8170 ±0.0198 | 0.7263 ±0.0089 | | | |

*Table S17: Logistic regression on Kaiko embeddings: Slide-level performance metrics NSCLC vs. MET.*

| Fold | Class | PRE | REC | F1 | BA | AUROC | MCC |
|---|---|---|---|---|---|---|---|
| Fold 1 | MET | 0.6250 | 0.4545 | 0.5263 | 0.6818 | 0.7052 | 0.4082 |
| | NSCLC | 0.8333 | 0.9091 | 0.8696 | | | |
| Fold 2 | MET | 0.6000 | 0.2727 | 0.3750 | 0.6061 | 0.7658 | 0.2894 |
| | NSCLC | 0.7949 | 0.9394 | 0.8611 | | | |
| Fold 3 | MET | 0.8571 | 0.5455 | 0.6667 | 0.7576 | 0.7961 | 0.6099 |
| | NSCLC | 0.8649 | 0.9697 | 0.9143 | | | |
| Fold 4 | MET | 0.6667 | 0.5455 | 0.6000 | 0.7273 | 0.8099 | 0.4880 |
| | NSCLC | 0.8571 | 0.9091 | 0.8824 | | | |
| Fold 5 | MET | 0.6667 | 0.5455 | 0.6000 | 0.7273 | 0.7989 | 0.4880 |
| | NSCLC | 0.8571 | 0.9091 | 0.8824 | | | |
| Fold 6 | MET | 0.7143 | 0.4545 | 0.5556 | 0.6970 | 0.8099 | 0.4664 |
| | NSCLC | 0.8378 | 0.9394 | 0.8857 | | | |
| Fold 7 | MET | 0.7143 | 0.4545 | 0.5556 | 0.6970 | 0.8044 | 0.4664 |
| | NSCLC | 0.8378 | 0.9394 | 0.8857 | | | |
| Fold 8 | MET | 0.6667 | 0.5455 | 0.6000 | 0.7273 | 0.7989 | 0.4880 |
| | NSCLC | 0.8571 | 0.9091 | 0.8824 | | | |
| Fold 9 | MET | 0.6000 | 0.5455 | 0.5714 | 0.7121 | 0.7521 | 0.4384 |
| | NSCLC | 0.8529 | 0.8788 | 0.8657 | | | |
| Fold 10 | MET | 0.7500 | 0.5455 | 0.6316 | 0.7424 | 0.7851 | 0.5443 |
| | NSCLC | 0.8611 | 0.9394 | 0.8986 | | | |
| **Mean ± std** | **MET** | 0.6861 ±0.0779 | 0.4909 ±0.0878 | 0.5682 ±0.0790 | 0.7076 ±0.0423 | 0.7826 ±0.0331 | 0.4687 ±0.0840 |
| | **NSCLC** | 0.8454 ±0.0208 | 0.9242 ±0.0258 | 0.8828 ±0.0156 | | | |

*Table S18: Logistic regression on Kaiko embeddings: Patient-level performance metrics NSCLC vs. MET.*

| Fold | Class | PRE | REC | F1 | BA | AUROC | MCC |
|---|---|---|---|---|---|---|---|
| Fold 1 | MET | 0.5000 | 0.3750 | 0.4286 | 0.6161 | 0.6964 | 0.2561 |
| | NSCLC | 0.7826 | 0.8571 | 0.8182 | | | |
| Fold 2 | MET | 0.6667 | 0.2500 | 0.3636 | 0.6012 | 0.7619 | 0.2970 |
| | NSCLC | 0.7692 | 0.9524 | 0.8511 | | | |
| Fold 3 | MET | 1.0000 | 0.5000 | 0.6667 | 0.7500 | 0.8155 | 0.6481 |
| | NSCLC | 0.8400 | 1.0000 | 0.9130 | | | |
| Fold 4 | MET | 1.0000 | 0.5000 | 0.6667 | 0.7500 | 0.8214 | 0.6481 |
| | NSCLC | 0.8400 | 1.0000 | 0.9130 | | | |
| Fold 5 | MET | 0.8000 | 0.5000 | 0.6154 | 0.7262 | 0.8036 | 0.5353 |
| | NSCLC | 0.8333 | 0.9524 | 0.8889 | | | |
| Fold 6 | MET | 1.0000 | 0.3750 | 0.5455 | 0.6875 | 0.8214 | 0.5503 |
| | NSCLC | 0.8077 | 1.0000 | 0.8936 | | | |
| Fold 7 | MET | 0.7500 | 0.3750 | 0.5000 | 0.6637 | 0.8214 | 0.4243 |
| | NSCLC | 0.8000 | 0.9524 | 0.8696 | | | |
| Fold 8 | MET | 1.0000 | 0.5000 | 0.6667 | 0.7500 | 0.7917 | 0.6481 |
| | NSCLC | 0.8400 | 1.0000 | 0.9130 | | | |
| Fold 9 | MET | 0.6667 | 0.5000 | 0.5714 | 0.7024 | 0.7619 | 0.4466 |
| | NSCLC | 0.8261 | 0.9048 | 0.8636 | | | |
| Fold 10 | MET | 0.8000 | 0.5000 | 0.6154 | 0.7262 | 0.7738 | 0.5353 |
| | NSCLC | 0.8333 | 0.9524 | 0.8889 | | | |
| **Mean ± std** | **MET** | 0.8183 ±0.1779 | 0.4375 ±0.0884 | 0.5640 ±0.1053 | 0.6973 ±0.0549 | 0.7869 ±0.0399 | 0.4989 ±0.1414 |
| | **NSCLC** | 0.8172 ±0.0259 | 0.9571 ±0.0474 | 0.8813 ±0.0310 | | | |

*Table S19: Logistic regression on Phikon embeddings: Patch-level performance metrics NSCLC vs. MET.*

| Fold | Class | PRE | REC | F1 | BA | AUROC | MCC |
|---|---|---|---|---|---|---|---|
| Fold 1 | MET | 0.6013 | 0.3162 | 0.4144 | 0.5819 | 0.6821 | 0.1947 |
| | NSCLC | 0.6303 | 0.8476 | 0.7230 | | | |
| Fold 2 | MET | 0.6264 | 0.3875 | 0.4788 | 0.6097 | 0.7085 | 0.2469 |
| | NSCLC | 0.6513 | 0.8320 | 0.7307 | | | |
| Fold 3 | MET | 0.6147 | 0.3591 | 0.4534 | 0.5977 | 0.6849 | 0.2241 |
| | NSCLC | 0.6422 | 0.8364 | 0.7265 | | | |
| Fold 4 | MET | 0.6277 | 0.3737 | 0.4685 | 0.6063 | 0.7185 | 0.2422 |
| | NSCLC | 0.6482 | 0.8389 | 0.7313 | | | |
| Fold 5 | MET | 0.6235 | 0.3557 | 0.4530 | 0.5998 | 0.6945 | 0.2306 |
| | NSCLC | 0.6430 | 0.8438 | 0.7299 | | | |
| Fold 6 | MET | 0.6270 | 0.3076 | 0.4127 | 0.5873 | 0.6754 | 0.2129 |
| | NSCLC | 0.6326 | 0.8670 | 0.7315 | | | |
| Fold 7 | MET | 0.6124 | 0.3393 | 0.4367 | 0.5916 | 0.6837 | 0.2139 |
| | NSCLC | 0.6373 | 0.8438 | 0.7261 | | | |
| Fold 8 | MET | 0.6300 | 0.3686 | 0.4650 | 0.6056 | 0.7022 | 0.2420 |
| | NSCLC | 0.6473 | 0.8426 | 0.7322 | | | |
| Fold 9 | MET | 0.5901 | 0.3771 | 0.4602 | 0.5933 | 0.6877 | 0.2078 |
| | NSCLC | 0.6413 | 0.8095 | 0.7156 | | | |
| Fold 10 | MET | 0.6238 | 0.3875 | 0.4780 | 0.6088 | 0.7113 | 0.2444 |
| | NSCLC | 0.6508 | 0.8301 | 0.7296 | | | |
| **Mean ± std** | **MET** | 0.6177 ±0.0131 | 0.3572 ±0.0281 | 0.4521 ±0.0238 | 0.5982 ±0.0095 | 0.6949 ±0.0145 | 0.2259 ±0.0181 |
| | **NSCLC** | 0.6424 ±0.0073 | 0.8392 ±0.0146 | 0.7276 ±0.0051 | | | |

*Table S20: Logistic regression on Phikon embeddings: Slide-level performance metrics NSCLC vs. MET.*

| Fold | Class | PRE | REC | F1 | BA | AUROC | MCC |
|---|---|---|---|---|---|---|---|
| Fold 1 | MET | 0.5000 | 0.3636 | 0.4211 | 0.6212 | 0.7521 | 0.2722 |
| | NSCLC | 0.8056 | 0.8788 | 0.8406 | | | |
| Fold 2 | MET | 0.5000 | 0.1818 | 0.2667 | 0.5606 | 0.7989 | 0.1826 |
| | NSCLC | 0.7750 | 0.9394 | 0.8493 | | | |
| Fold 3 | MET | 0.6667 | 0.3636 | 0.4706 | 0.6515 | 0.7851 | 0.3824 |
| | NSCLC | 0.8158 | 0.9394 | 0.8732 | | | |
| Fold 4 | MET | 0.6667 | 0.3636 | 0.4706 | 0.6515 | 0.8320 | 0.3824 |
| | NSCLC | 0.8158 | 0.9394 | 0.8732 | | | |
| Fold 5 | MET | 0.6667 | 0.3636 | 0.4706 | 0.6515 | 0.8237 | 0.3824 |
| | NSCLC | 0.8158 | 0.9394 | 0.8732 | | | |
| Fold 6 | MET | 0.8000 | 0.3636 | 0.5000 | 0.6667 | 0.8678 | 0.4548 |
| | NSCLC | 0.8205 | 0.9697 | 0.8889 | | | |
| Fold 7 | MET | 0.6667 | 0.3636 | 0.4706 | 0.6515 | 0.8127 | 0.3824 |
| | NSCLC | 0.8158 | 0.9394 | 0.8732 | | | |
| Fold 8 | MET | 0.7500 | 0.5455 | 0.6316 | 0.7424 | 0.7934 | 0.5443 |
| | NSCLC | 0.8611 | 0.9394 | 0.8986 | | | |
| Fold 9 | MET | 0.5714 | 0.3636 | 0.4444 | 0.6364 | 0.8072 | 0.3229 |
| | NSCLC | 0.8108 | 0.9091 | 0.8571 | | | |
| Fold 10 | MET | 0.6667 | 0.3636 | 0.4706 | 0.6515 | 0.8292 | 0.3824 |
| | NSCLC | 0.8158 | 0.9394 | 0.8732 | | | |
| **Mean ± std** | **MET** | 0.6455 ±0.0971 | 0.3636 ±0.0857 | 0.4617 ±0.0886 | 0.6485 ±0.0445 | 0.8102 ±0.0312 | 0.3689 ±0.0973 |
| | **NSCLC** | 0.8152 ±0.0207 | 0.9333 ±0.0239 | 0.8701 ±0.0173 | | | |

*Table S21: Logistic regression on Phikon embeddings: Patient-level performance metrics NSCLC vs. MET.*

| Fold | Class | PRE | REC | F1 | BA | AUROC | MCC |
|---|---|---|---|---|---|---|---|
| Fold 1 | MET | 1.0000 | 0.2500 | 0.4000 | 0.6250 | 0.7500 | 0.4410 |
| | NSCLC | 0.7778 | 1.0000 | 0.8750 | | | |
| Fold 2 | MET | 0.5000 | 0.1250 | 0.2000 | 0.5387 | 0.8274 | 0.1365 |
| | NSCLC | 0.7407 | 0.9524 | 0.8333 | | | |
| Fold 3 | MET | 1.0000 | 0.2500 | 0.4000 | 0.6250 | 0.8095 | 0.4410 |
| | NSCLC | 0.7778 | 1.0000 | 0.8750 | | | |
| Fold 4 | MET | 1.0000 | 0.2500 | 0.4000 | 0.6250 | 0.8274 | 0.4410 |
| | NSCLC | 0.7778 | 1.0000 | 0.8750 | | | |
| Fold 5 | MET | 1.0000 | 0.2500 | 0.4000 | 0.6250 | 0.8095 | 0.4410 |
| | NSCLC | 0.7778 | 1.0000 | 0.8750 | | | |
| Fold 6 | MET | 0.6667 | 0.2500 | 0.3636 | 0.6012 | 0.8869 | 0.2970 |
| | NSCLC | 0.7692 | 0.9524 | 0.8511 | | | |
| Fold 7 | MET | 0.6667 | 0.2500 | 0.3636 | 0.6012 | 0.7976 | 0.2970 |
| | NSCLC | 0.7692 | 0.9524 | 0.8511 | | | |
| Fold 8 | MET | 1.0000 | 0.5000 | 0.6667 | 0.7500 | 0.7798 | 0.6481 |
| | NSCLC | 0.8400 | 1.0000 | 0.9130 | | | |
| Fold 9 | MET | 0.6667 | 0.2500 | 0.3636 | 0.6012 | 0.8095 | 0.2970 |
| | NSCLC | 0.7692 | 0.9524 | 0.8511 | | | |
| Fold 10 | MET | 1.0000 | 0.2500 | 0.4000 | 0.6250 | 0.8333 | 0.4410 |
| | NSCLC | 0.7778 | 1.0000 | 0.8750 | | | |
| **Mean ± std** | **MET** | 0.8500 ±0.1995 | 0.2625 ±0.0922 | 0.3958 ±0.1130 | 0.6217 ±0.0524 | 0.8131 ±0.0360 | 0.3880 ±0.1373 |
| | **NSCLC** | 0.7777 ±0.0246 | 0.9810 ±0.0246 | 0.8675 ±0.0219 | | | |

*Table S22: Logistic regression on ProvGigaPath embeddings: Patch-level performance metrics NSCLC vs. MET.*

| Fold | Class | PRE | REC | F1 | BA | AUROC | MCC |
|---|---|---|---|---|---|---|---|
| Fold 1 | MET | 0.6394 | 0.2955 | 0.4042 | 0.5872 | 0.6835 | 0.2175 |
| | NSCLC | 0.6318 | 0.8788 | 0.7351 | | | |
| Fold 2 | MET | 0.6736 | 0.3600 | 0.4692 | 0.6166 | 0.7275 | 0.2757 |
| | NSCLC | 0.6524 | 0.8732 | 0.7468 | | | |
| Fold 3 | MET | 0.6588 | 0.3600 | 0.4656 | 0.6122 | 0.6975 | 0.2633 |
| | NSCLC | 0.6501 | 0.8645 | 0.7421 | | | |
| Fold 4 | MET | 0.6642 | 0.3840 | 0.4867 | 0.6214 | 0.7179 | 0.2794 |
| | NSCLC | 0.6573 | 0.8588 | 0.7447 | | | |
| Fold 5 | MET | 0.6992 | 0.3694 | 0.4834 | 0.6269 | 0.7392 | 0.3014 |
| | NSCLC | 0.6586 | 0.8844 | 0.7550 | | | |
| Fold 6 | MET | 0.6333 | 0.2878 | 0.3957 | 0.5833 | 0.6619 | 0.2091 |
| | NSCLC | 0.6292 | 0.8788 | 0.7334 | | | |
| Fold 7 | MET | 0.6820 | 0.3686 | 0.4785 | 0.6218 | 0.7257 | 0.2869 |
| | NSCLC | 0.6559 | 0.8751 | 0.7498 | | | |
| Fold 8 | MET | 0.6643 | 0.3162 | 0.4284 | 0.6000 | 0.6939 | 0.2467 |
| | NSCLC | 0.6400 | 0.8838 | 0.7424 | | | |
| Fold 9 | MET | 0.6473 | 0.3737 | 0.4739 | 0.6128 | 0.7041 | 0.2598 |
| | NSCLC | 0.6517 | 0.8520 | 0.7385 | | | |
| Fold 10 | MET | 0.6682 | 0.3720 | 0.4779 | 0.6189 | 0.7299 | 0.2770 |
| | NSCLC | 0.6547 | 0.8657 | 0.7456 | | | |
| **Mean ± std** | **MET** | 0.6630 ±0.0198 | 0.3487 ±0.0351 | 0.4564 ±0.0339 | 0.6101 ±0.0150 | 0.7081 ±0.0243 | 0.2617 ±0.0296 |
| | **NSCLC** | 0.6482 ±0.0107 | 0.8715 ±0.0109 | 0.7433 ±0.0066 | | | |

*Table S23: Logistic regression on ProvGigaPath embeddings: Slide-level performance metrics NSCLC vs. MET.*

| Fold | Class | PRE | REC | F1 | BA | AUROC | MCC |
|---|---|---|---|---|---|---|---|
| Fold 1 | MET | 0.4000 | 0.3636 | 0.3810 | 0.5909 | 0.6777 | 0.1879 |
| | NSCLC | 0.7941 | 0.8182 | 0.8060 | | | |
| Fold 2 | MET | 0.4000 | 0.1818 | 0.2500 | 0.5455 | 0.7273 | 0.1240 |
| | NSCLC | 0.7692 | 0.9091 | 0.8333 | | | |
| Fold 3 | MET | 0.5714 | 0.3636 | 0.4444 | 0.6364 | 0.7658 | 0.3229 |
| | NSCLC | 0.8108 | 0.9091 | 0.8571 | | | |
| Fold 4 | MET | 0.5714 | 0.3636 | 0.4444 | 0.6364 | 0.7631 | 0.3229 |
| | NSCLC | 0.8108 | 0.9091 | 0.8571 | | | |
| Fold 5 | MET | 0.7143 | 0.4545 | 0.5556 | 0.6970 | 0.7713 | 0.4664 |
| | NSCLC | 0.8378 | 0.9394 | 0.8857 | | | |
| Fold 6 | MET | 0.5714 | 0.3636 | 0.4444 | 0.6364 | 0.7824 | 0.3229 |
| | NSCLC | 0.8108 | 0.9091 | 0.8571 | | | |
| Fold 7 | MET | 0.5714 | 0.3636 | 0.4444 | 0.6364 | 0.7603 | 0.3229 |
| | NSCLC | 0.8108 | 0.9091 | 0.8571 | | | |
| Fold 8 | MET | 0.5714 | 0.3636 | 0.4444 | 0.6364 | 0.7576 | 0.3229 |
| | NSCLC | 0.8108 | 0.9091 | 0.8571 | | | |
| Fold 9 | MET | 0.5714 | 0.3636 | 0.4444 | 0.6364 | 0.7355 | 0.3229 |
| | NSCLC | 0.8108 | 0.9091 | 0.8571 | | | |
| Fold 10 | MET | 0.6667 | 0.3636 | 0.4706 | 0.6515 | 0.7741 | 0.3824 |
| | NSCLC | 0.8158 | 0.9394 | 0.8732 | | | |
| **Mean ± std** | **MET** | 0.5610 ±0.0984 | 0.3545 ±0.0671 | 0.4324 ±0.0770 | 0.6303 ±0.0393 | 0.7515 ±0.0309 | 0.3098 ±0.0944 |
| | **NSCLC** | 0.8082 ±0.0173 | 0.9061 ±0.0333 | 0.8541 ±0.0215 | | | |

*Table S24: Logistic regression on ProvGigaPath embeddings: Patient-level performance metrics NSCLC vs. MET.*

| Fold | Class | PRE | REC | F1 | BA | AUROC | MCC |
|---|---|---|---|---|---|---|---|
| Fold 1 | MET | 0.5000 | 0.2500 | 0.3333 | 0.5774 | 0.6548 | 0.2006 |
| | NSCLC | 0.7600 | 0.9048 | 0.8261 | | | |
| Fold 2 | MET | 0.5000 | 0.1250 | 0.2000 | 0.5387 | 0.7917 | 0.1365 |
| | NSCLC | 0.7407 | 0.9524 | 0.8333 | | | |
| Fold 3 | MET | 0.6667 | 0.2500 | 0.3636 | 0.6012 | 0.8036 | 0.2970 |
| | NSCLC | 0.7692 | 0.9524 | 0.8511 | | | |
| Fold 4 | MET | 0.6667 | 0.2500 | 0.3636 | 0.6012 | 0.7917 | 0.2970 |
| | NSCLC | 0.7692 | 0.9524 | 0.8511 | | | |
| Fold 5 | MET | 0.7500 | 0.3750 | 0.5000 | 0.6637 | 0.7917 | 0.4243 |
| | NSCLC | 0.8000 | 0.9524 | 0.8696 | | | |
| Fold 6 | MET | 0.5000 | 0.2500 | 0.3333 | 0.5774 | 0.7857 | 0.2006 |
| | NSCLC | 0.7600 | 0.9048 | 0.8261 | | | |
| Fold 7 | MET | 0.6667 | 0.2500 | 0.3636 | 0.6012 | 0.8036 | 0.2970 |
| | NSCLC | 0.7692 | 0.9524 | 0.8511 | | | |
| Fold 8 | MET | 0.6667 | 0.2500 | 0.3636 | 0.6012 | 0.7619 | 0.2970 |
| | NSCLC | 0.7692 | 0.9524 | 0.8511 | | | |
| Fold 9 | MET | 0.5000 | 0.2500 | 0.3333 | 0.5774 | 0.7738 | 0.2006 |
| | NSCLC | 0.7600 | 0.9048 | 0.8261 | | | |
| Fold 10 | MET | 0.6667 | 0.2500 | 0.3636 | 0.6012 | 0.7976 | 0.2970 |
| | NSCLC | 0.7692 | 0.9524 | 0.8511 | | | |
| **Mean ± std** | **MET** | 0.6083 ±0.0966 | 0.2500 ±0.0589 | 0.3518 ±0.0721 | 0.5940 ±0.0316 | 0.7756 ±0.0444 | 0.2648 ±0.0813 |
| | **NSCLC** | 0.7667 ±0.0147 | 0.9381 ±0.0230 | 0.8436 ±0.0148 | | | |

*Table S25: Logistic regression on ResNet50 embeddings: Patch-level performance metrics NSCLC vs. MET.*

| Fold | Class | PRE | REC | F1 | BA | AUROC | MCC |
|---|---|---|---|---|---|---|---|
| Fold 1 | MET | 0.6089 | 0.3771 | 0.4658 | 0.6005 | 0.6681 | 0.2260 |
| | NSCLC | 0.6453 | 0.8239 | 0.7237 | | | |
| Fold 2 | MET | 0.5989 | 0.3771 | 0.4628 | 0.5968 | 0.6787 | 0.2165 |
| | NSCLC | 0.6432 | 0.8164 | 0.7195 | | | |
| Fold 3 | MET | 0.5828 | 0.3900 | 0.4673 | 0.5935 | 0.6564 | 0.2053 |
| | NSCLC | 0.6425 | 0.7970 | 0.7115 | | | |
| Fold 4 | MET | 0.5972 | 0.4381 | 0.5055 | 0.6116 | 0.6699 | 0.2386 |
| | NSCLC | 0.6578 | 0.7851 | 0.7158 | | | |
| Fold 5 | MET | 0.5900 | 0.3857 | 0.4665 | 0.5954 | 0.6631 | 0.2110 |
| | NSCLC | 0.6432 | 0.8051 | 0.7151 | | | |
| Fold 6 | MET | 0.6392 | 0.4399 | 0.5211 | 0.6297 | 0.6851 | 0.2823 |
| | NSCLC | 0.6680 | 0.8195 | 0.7360 | | | |
| Fold 7 | MET | 0.6101 | 0.3832 | 0.4707 | 0.6026 | 0.6643 | 0.2297 |
| | NSCLC | 0.6470 | 0.8220 | 0.7241 | | | |
| Fold 8 | MET | 0.5838 | 0.3651 | 0.4493 | 0.5879 | 0.6475 | 0.1971 |
| | NSCLC | 0.6372 | 0.8107 | 0.7136 | | | |
| Fold 9 | MET | 0.6088 | 0.4399 | 0.5107 | 0.6172 | 0.6779 | 0.2515 |
| | NSCLC | 0.6611 | 0.7945 | 0.7217 | | | |
| Fold 10 | MET | 0.6117 | 0.4235 | 0.5005 | 0.6140 | 0.6753 | 0.2477 |
| | NSCLC | 0.6575 | 0.8045 | 0.7236 | | | |
| **Mean ± std** | **MET** | 0.6031 ±0.0167 | 0.4020 ±0.0298 | 0.4820 ±0.0248 | 0.6049 ±0.0129 | 0.6686 ±0.0113 | 0.2306 ±0.0255 |
| | **NSCLC** | 0.6503 ±0.0100 | 0.8079 ±0.0129 | 0.7205 ±0.0071 | | | |

*Table S26: Logistic regression on ResNet50 embeddings: Slide-level performance metrics NSCLC vs. MET.*

| Fold | Class | PRE | REC | F1 | BA | AUROC | MCC |
|---|---|---|---|---|---|---|---|
| Fold 1 | MET | 0.6250 | 0.4545 | 0.5263 | 0.6818 | 0.7713 | 0.4082 |
| | NSCLC | 0.8333 | 0.9091 | 0.8696 | | | |
| Fold 2 | MET | 0.5000 | 0.1818 | 0.2667 | 0.5606 | 0.7383 | 0.1826 |
| | NSCLC | 0.7750 | 0.9394 | 0.8493 | | | |
| Fold 3 | MET | 0.5714 | 0.3636 | 0.4444 | 0.6364 | 0.7548 | 0.3229 |
| | NSCLC | 0.8108 | 0.9091 | 0.8571 | | | |
| Fold 4 | MET | 0.6250 | 0.4545 | 0.5263 | 0.6818 | 0.7934 | 0.4082 |
| | NSCLC | 0.8333 | 0.9091 | 0.8696 | | | |
| Fold 5 | MET | 0.6250 | 0.4545 | 0.5263 | 0.6818 | 0.7493 | 0.4082 |
| | NSCLC | 0.8333 | 0.9091 | 0.8696 | | | |
| Fold 6 | MET | 0.5000 | 0.5455 | 0.5217 | 0.6818 | 0.8320 | 0.3536 |
| | NSCLC | 0.8438 | 0.8182 | 0.8308 | | | |
| Fold 7 | MET | 0.5714 | 0.3636 | 0.4444 | 0.6364 | 0.7851 | 0.3229 |
| | NSCLC | 0.8108 | 0.9091 | 0.8571 | | | |
| Fold 8 | MET | 0.8333 | 0.4545 | 0.5882 | 0.7121 | 0.8127 | 0.5353 |
| | NSCLC | 0.8421 | 0.9697 | 0.9014 | | | |
| Fold 9 | MET | 0.6250 | 0.4545 | 0.5263 | 0.6818 | 0.8127 | 0.4082 |
| | NSCLC | 0.8333 | 0.9091 | 0.8696 | | | |
| Fold 10 | MET | 0.5556 | 0.4545 | 0.5000 | 0.6667 | 0.7934 | 0.3578 |
| | NSCLC | 0.8286 | 0.8788 | 0.8529 | | | |
| **Mean ± std** | **MET** | 0.6032 ±0.0945 | 0.4182 ±0.0977 | 0.4871 ±0.0882 | 0.6621 ±0.0423 | 0.7843 ±0.0306 | 0.3708 ±0.0902 |
| | **NSCLC** | 0.8244 ±0.0206 | 0.9061 ±0.0390 | 0.8627 ±0.0184 | | | |

*Table S27: Logistic regression on ResNet50 embeddings: Patient-level performance metrics NSCLC vs. MET.*

| Fold | Class | PRE | REC | F1 | BA | AUROC | MCC |
|---|---|---|---|---|---|---|---|
| Fold 1 | MET | 0.7500 | 0.3750 | 0.5000 | 0.6637 | 0.8095 | 0.4243 |
| | NSCLC | 0.8000 | 0.9524 | 0.8696 | | | |
| Fold 2 | MET | 0.5000 | 0.1250 | 0.2000 | 0.5387 | 0.7857 | 0.1365 |
| | NSCLC | 0.7407 | 0.9524 | 0.8333 | | | |
| Fold 3 | MET | 0.6000 | 0.3750 | 0.4615 | 0.6399 | 0.7857 | 0.3310 |
| | NSCLC | 0.7917 | 0.9048 | 0.8444 | | | |
| Fold 4 | MET | 0.5000 | 0.3750 | 0.4286 | 0.6161 | 0.8155 | 0.2561 |
| | NSCLC | 0.7826 | 0.8571 | 0.8182 | | | |
| Fold 5 | MET | 0.7500 | 0.3750 | 0.5000 | 0.6637 | 0.7679 | 0.4243 |
| | NSCLC | 0.8000 | 0.9524 | 0.8696 | | | |
| Fold 6 | MET | 0.5000 | 0.3750 | 0.4286 | 0.6161 | 0.8690 | 0.2561 |
| | NSCLC | 0.7826 | 0.8571 | 0.8182 | | | |
| Fold 7 | MET | 0.7500 | 0.3750 | 0.5000 | 0.6637 | 0.8214 | 0.4243 |
| | NSCLC | 0.8000 | 0.9524 | 0.8696 | | | |
| Fold 8 | MET | 1.0000 | 0.3750 | 0.5455 | 0.6875 | 0.8333 | 0.5503 |
| | NSCLC | 0.8077 | 1.0000 | 0.8936 | | | |
| Fold 9 | MET | 0.7500 | 0.3750 | 0.5000 | 0.6637 | 0.8393 | 0.4243 |
| | NSCLC | 0.8000 | 0.9524 | 0.8696 | | | |
| Fold 10 | MET | 0.5000 | 0.3750 | 0.4286 | 0.6161 | 0.8333 | 0.2561 |
| | NSCLC | 0.7826 | 0.8571 | 0.8182 | | | |
| **Mean ± std** | **MET** | 0.6600 ±0.1680 | 0.3500 ±0.0791 | 0.4493 ±0.0961 | 0.6369 ±0.0427 | 0.8161 ±0.0301 | 0.3484 ±0.1223 |
| | **NSCLC** | 0.7888 ±0.0191 | 0.9238 ±0.0512 | 0.8504 ±0.0274 | | | |

*Table S28: Logistic regression on TissueConcepts embeddings: Patch-level performance metrics NSCLC vs. MET.*

| Fold | Class | PRE | REC | F1 | BA | AUROC | MCC |
|---|---|---|---|---|---|---|---|
| Fold 1 | MET | 0.6211 | 0.4648 | 0.5317 | 0.6293 | 0.6997 | 0.2749 |
| | NSCLC | 0.6711 | 0.7939 | 0.7273 | | | |
| Fold 2 | MET | 0.6207 | 0.4905 | 0.5480 | 0.6363 | 0.7066 | 0.2856 |
| | NSCLC | 0.6786 | 0.7820 | 0.7266 | | | |
| Fold 3 | MET | 0.6093 | 0.5172 | 0.5595 | 0.6380 | 0.6911 | 0.2844 |
| | NSCLC | 0.6837 | 0.7589 | 0.7194 | | | |
| Fold 4 | MET | 0.6155 | 0.5447 | 0.5779 | 0.6487 | 0.7117 | 0.3036 |
| | NSCLC | 0.6945 | 0.7527 | 0.7224 | | | |
| Fold 5 | MET | 0.6132 | 0.4725 | 0.5337 | 0.6279 | 0.6931 | 0.2697 |
| | NSCLC | 0.6713 | 0.7833 | 0.7230 | | | |
| Fold 6 | MET | 0.6655 | 0.5026 | 0.5727 | 0.6595 | 0.7077 | 0.3382 |
| | NSCLC | 0.6930 | 0.8164 | 0.7496 | | | |
| Fold 7 | MET | 0.6256 | 0.4708 | 0.5373 | 0.6330 | 0.7036 | 0.2822 |
| | NSCLC | 0.6739 | 0.7951 | 0.7295 | | | |
| Fold 8 | MET | 0.6168 | 0.4605 | 0.5273 | 0.6262 | 0.6904 | 0.2685 |
| | NSCLC | 0.6688 | 0.7920 | 0.7252 | | | |
| Fold 9 | MET | 0.6176 | 0.5258 | 0.5680 | 0.6445 | 0.7029 | 0.2976 |
| | NSCLC | 0.6888 | 0.7633 | 0.7241 | | | |
| Fold 10 | MET | 0.6231 | 0.5198 | 0.5667 | 0.6456 | 0.7138 | 0.3011 |
| | NSCLC | 0.6884 | 0.7714 | 0.7275 | | | |
| **Mean ± std** | **MET** | 0.6228 ±0.0157 | 0.4969 ±0.0294 | 0.5523 ±0.0189 | 0.6389 ±0.0106 | 0.7021 ±0.0084 | 0.2906 ±0.0208 |
| | **NSCLC** | 0.6812 ±0.0097 | 0.7809 ±0.0196 | 0.7275 ±0.0083 | | | |

*Table S29: Logistic regression on TissueConcepts embeddings: Slide-level performance metrics NSCLC vs. MET.*

| Fold | Class | PRE | REC | F1 | BA | AUROC | MCC |
|---|---|---|---|---|---|---|---|
| Fold 1 | MET | 0.6250 | 0.4545 | 0.5263 | 0.6818 | 0.8072 | 0.4082 |
| | NSCLC | 0.8333 | 0.9091 | 0.8696 | | | |
| Fold 2 | MET | 0.4000 | 0.1818 | 0.2500 | 0.5455 | 0.7025 | 0.1240 |
| | NSCLC | 0.7692 | 0.9091 | 0.8333 | | | |
| Fold 3 | MET | 0.7500 | 0.5455 | 0.6316 | 0.7424 | 0.8237 | 0.5443 |
| | NSCLC | 0.8611 | 0.9394 | 0.8986 | | | |
| Fold 4 | MET | 0.6364 | 0.6364 | 0.6364 | 0.7576 | 0.8540 | 0.5152 |
| | NSCLC | 0.8788 | 0.8788 | 0.8788 | | | |
| Fold 5 | MET | 0.5556 | 0.4545 | 0.5000 | 0.6667 | 0.8099 | 0.3578 |
| | NSCLC | 0.8286 | 0.8788 | 0.8529 | | | |
| Fold 6 | MET | 0.6364 | 0.6364 | 0.6364 | 0.7576 | 0.8898 | 0.5152 |
| | NSCLC | 0.8788 | 0.8788 | 0.8788 | | | |
| Fold 7 | MET | 0.7143 | 0.4545 | 0.5556 | 0.6970 | 0.8733 | 0.4664 |
| | NSCLC | 0.8378 | 0.9394 | 0.8857 | | | |
| Fold 8 | MET | 0.7143 | 0.4545 | 0.5556 | 0.6970 | 0.8650 | 0.4664 |
| | NSCLC | 0.8378 | 0.9394 | 0.8857 | | | |
| Fold 9 | MET | 0.6000 | 0.5455 | 0.5714 | 0.7121 | 0.8678 | 0.4384 |
| | NSCLC | 0.8529 | 0.8788 | 0.8657 | | | |
| Fold 10 | MET | 0.7778 | 0.6364 | 0.7000 | 0.7879 | 0.8705 | 0.6181 |
| | NSCLC | 0.8857 | 0.9394 | 0.9118 | | | |
| **Mean ± std** | **MET** | 0.6410 ±0.1099 | 0.5000 ±0.1372 | 0.5563 ±0.1235 | 0.7045 ±0.0679 | 0.8364 ±0.0549 | 0.4454 ±0.1344 |
| | **NSCLC** | 0.8464 ±0.0341 | 0.9091 ±0.0286 | 0.8761 ±0.0223 | | | |

*Table S30: Logistic regression on TissueConcepts embeddings: Patient-level performance metrics NSCLC vs. MET.*

| Fold | Class | PRE | REC | F1 | BA | AUROC | MCC |
|---|---|---|---|---|---|---|---|
| Fold 1 | MET | 0.6000 | 0.3750 | 0.4615 | 0.6399 | 0.8095 | 0.3310 |
| | NSCLC | 0.7917 | 0.9048 | 0.8444 | | | |
| Fold 2 | MET | 0.6667 | 0.2500 | 0.3636 | 0.6012 | 0.7560 | 0.2970 |
| | NSCLC | 0.7692 | 0.9524 | 0.8511 | | | |
| Fold 3 | MET | 0.8000 | 0.5000 | 0.6154 | 0.7262 | 0.8631 | 0.5353 |
| | NSCLC | 0.8333 | 0.9524 | 0.8889 | | | |
| Fold 4 | MET | 0.7143 | 0.6250 | 0.6667 | 0.7649 | 0.8750 | 0.5533 |
| | NSCLC | 0.8636 | 0.9048 | 0.8837 | | | |
| Fold 5 | MET | 0.7500 | 0.3750 | 0.5000 | 0.6637 | 0.8333 | 0.4243 |
| | NSCLC | 0.8000 | 0.9524 | 0.8696 | | | |
| Fold 6 | MET | 0.8000 | 0.5000 | 0.6154 | 0.7262 | 0.9048 | 0.5353 |
| | NSCLC | 0.8333 | 0.9524 | 0.8889 | | | |
| Fold 7 | MET | 0.7500 | 0.3750 | 0.5000 | 0.6637 | 0.8929 | 0.4243 |
| | NSCLC | 0.8000 | 0.9524 | 0.8696 | | | |
| Fold 8 | MET | 0.7500 | 0.3750 | 0.5000 | 0.6637 | 0.9048 | 0.4243 |
| | NSCLC | 0.8000 | 0.9524 | 0.8696 | | | |
| Fold 9 | MET | 0.6667 | 0.5000 | 0.5714 | 0.7024 | 0.8929 | 0.4466 |
| | NSCLC | 0.8261 | 0.9048 | 0.8636 | | | |
| Fold 10 | MET | 0.8333 | 0.6250 | 0.7143 | 0.7887 | 0.9048 | 0.6371 |
| | NSCLC | 0.8696 | 0.9524 | 0.9091 | | | |
| **Mean ± std** | **MET** | 0.7331 ±0.0721 | 0.4500 ±0.1208 | 0.5508 ±0.1052 | 0.6940 ±0.0581 | 0.8637 ±0.0498 | 0.4609 ±0.1047 |
| | **NSCLC** | 0.8187 ±0.0322 | 0.9381 ±0.0230 | 0.8738 ±0.0192 | | | |

*Table S31: Logistic regression on UNI2-h embeddings: Patch-level performance metrics NSCLC vs. MET.*

| Fold | Class | PRE | REC | F1 | BA | AUROC | MCC |
|---|---|---|---|---|---|---|---|
| Fold 1 | MET | 0.6083 | 0.2895 | 0.3923 | 0.5770 | 0.6807 | 0.1899 |
| | NSCLC | 0.6260 | 0.8645 | 0.7261 | | | |
| Fold 2 | MET | 0.6477 | 0.3428 | 0.4483 | 0.6036 | 0.7379 | 0.2459 |
| | NSCLC | 0.6440 | 0.8645 | 0.7381 | | | |
| Fold 3 | MET | 0.6403 | 0.3686 | 0.4678 | 0.6090 | 0.7183 | 0.2512 |
| | NSCLC | 0.6492 | 0.8495 | 0.7359 | | | |
| Fold 4 | MET | 0.6266 | 0.3677 | 0.4635 | 0.6042 | 0.7117 | 0.2386 |
| | NSCLC | 0.6465 | 0.8407 | 0.7309 | | | |
| Fold 5 | MET | 0.6164 | 0.3230 | 0.4239 | 0.5884 | 0.6946 | 0.2106 |
| | NSCLC | 0.6343 | 0.8538 | 0.7279 | | | |
| Fold 6 | MET | 0.6316 | 0.3093 | 0.4152 | 0.5891 | 0.6742 | 0.2174 |
| | NSCLC | 0.6337 | 0.8688 | 0.7329 | | | |
| Fold 7 | MET | 0.6276 | 0.3170 | 0.4212 | 0.5901 | 0.7038 | 0.2174 |
| | NSCLC | 0.6348 | 0.8632 | 0.7316 | | | |
| Fold 8 | MET | 0.6119 | 0.3170 | 0.4177 | 0.5854 | 0.6799 | 0.2043 |
| | NSCLC | 0.6323 | 0.8538 | 0.7265 | | | |
| Fold 9 | MET | 0.5962 | 0.3514 | 0.4422 | 0.5892 | 0.6820 | 0.2039 |
| | NSCLC | 0.6368 | 0.8270 | 0.7196 | | | |
| Fold 10 | MET | 0.6277 | 0.3505 | 0.4498 | 0.5997 | 0.7075 | 0.2321 |
| | NSCLC | 0.6426 | 0.8488 | 0.7314 | | | |
| **Mean ± std** | **MET** | 0.6234 ±0.0154 | 0.3337 ±0.0264 | 0.4342 ±0.0239 | 0.5936 ±0.0101 | 0.6991 ±0.0204 | 0.2211 ±0.0201 |
| | **NSCLC** | 0.6380 ±0.0073 | 0.8535 ±0.0128 | 0.7301 ±0.0053 | | | |

*Table S32: Logistic regression on UNI2-h embeddings: Slide-level performance metrics NSCLC vs. MET.*

| Fold | Class | PRE | REC | F1 | BA | AUROC | MCC |
|---|---|---|---|---|---|---|---|
| Fold 1 | MET | 0.8000 | 0.3636 | 0.5000 | 0.6667 | 0.7410 | 0.4548 |
| | NSCLC | 0.8205 | 0.9697 | 0.8889 | | | |
| Fold 2 | MET | 0.4000 | 0.1818 | 0.2500 | 0.5455 | 0.8430 | 0.1240 |
| | NSCLC | 0.7692 | 0.9091 | 0.8333 | | | |
| Fold 3 | MET | 0.6667 | 0.3636 | 0.4706 | 0.6515 | 0.8072 | 0.3824 |
| | NSCLC | 0.8158 | 0.9394 | 0.8732 | | | |
| Fold 4 | MET | 0.6667 | 0.3636 | 0.4706 | 0.6515 | 0.7879 | 0.3824 |
| | NSCLC | 0.8158 | 0.9394 | 0.8732 | | | |
| Fold 5 | MET | 0.8000 | 0.3636 | 0.5000 | 0.6667 | 0.7796 | 0.4548 |
| | NSCLC | 0.8205 | 0.9697 | 0.8889 | | | |
| Fold 6 | MET | 0.6667 | 0.3636 | 0.4706 | 0.6515 | 0.7686 | 0.3824 |
| | NSCLC | 0.8158 | 0.9394 | 0.8732 | | | |
| Fold 7 | MET | 0.6667 | 0.3636 | 0.4706 | 0.6515 | 0.8127 | 0.3824 |
| | NSCLC | 0.8158 | 0.9394 | 0.8732 | | | |
| Fold 8 | MET | 0.8000 | 0.3636 | 0.5000 | 0.6667 | 0.7631 | 0.4548 |
| | NSCLC | 0.8205 | 0.9697 | 0.8889 | | | |
| Fold 9 | MET | 0.5000 | 0.3636 | 0.4211 | 0.6212 | 0.7631 | 0.2722 |
| | NSCLC | 0.8056 | 0.8788 | 0.8406 | | | |
| Fold 10 | MET | 0.8000 | 0.3636 | 0.5000 | 0.6667 | 0.8099 | 0.4548 |
| | NSCLC | 0.8205 | 0.9697 | 0.8889 | | | |
| **Mean ± std** | **MET** | 0.6767 ±0.1370 | 0.3455 ±0.0575 | 0.4553 ±0.0762 | 0.6439 ±0.0373 | 0.7876 ±0.0305 | 0.3745 ±0.1050 |
| | **NSCLC** | 0.8120 ±0.0157 | 0.9424 ±0.0301 | 0.8722 ±0.0201 | | | |

*Table S33: Logistic regression on UNI2-h embeddings: Patient-level performance metrics NSCLC vs. MET.*

| Fold | Class | PRE | REC | F1 | BA | AUROC | MCC |
|---|---|---|---|---|---|---|---|
| Fold 1 | MET | 1.0000 | 0.2500 | 0.4000 | 0.6250 | 0.7143 | 0.4410 |
| | NSCLC | 0.7778 | 1.0000 | 0.8750 | | | |
| Fold 2 | MET | 0.0000 | 0.0000 | 0.0000 | 0.4762 | 0.8512 | -0.1166 |
| | NSCLC | 0.7143 | 0.9524 | 0.8163 | | | |
| Fold 3 | MET | 0.6667 | 0.2500 | 0.3636 | 0.6012 | 0.8214 | 0.2970 |
| | NSCLC | 0.7692 | 0.9524 | 0.8511 | | | |
| Fold 4 | MET | 1.0000 | 0.2500 | 0.4000 | 0.6250 | 0.8036 | 0.4410 |
| | NSCLC | 0.7778 | 1.0000 | 0.8750 | | | |
| Fold 5 | MET | 1.0000 | 0.2500 | 0.4000 | 0.6250 | 0.7738 | 0.4410 |
| | NSCLC | 0.7778 | 1.0000 | 0.8750 | | | |
| Fold 6 | MET | 1.0000 | 0.2500 | 0.4000 | 0.6250 | 0.7619 | 0.4410 |
| | NSCLC | 0.7778 | 1.0000 | 0.8750 | | | |
| Fold 7 | MET | 1.0000 | 0.2500 | 0.4000 | 0.6250 | 0.7976 | 0.4410 |
| | NSCLC | 0.7778 | 1.0000 | 0.8750 | | | |
| Fold 8 | MET | 1.0000 | 0.2500 | 0.4000 | 0.6250 | 0.7500 | 0.4410 |
| | NSCLC | 0.7778 | 1.0000 | 0.8750 | | | |
| Fold 9 | MET | 0.5000 | 0.2500 | 0.3333 | 0.5774 | 0.7560 | 0.2006 |
| | NSCLC | 0.7600 | 0.9048 | 0.8261 | | | |
| Fold 10 | MET | 1.0000 | 0.2500 | 0.4000 | 0.6250 | 0.8095 | 0.4410 |
| | NSCLC | 0.7778 | 1.0000 | 0.8750 | | | |
| **Mean ± std** | **MET** | 0.8167 ±0.3375 | 0.2250 ±0.0791 | 0.3497 ±0.1249 | 0.6030 ±0.0473 | 0.7839 ±0.0402 | 0.3468 ±0.1828 |
| | **NSCLC** | 0.7688 ±0.0200 | 0.9810 ±0.0333 | 0.8618 ±0.0228 | | | |

*Table S34: Logistic regression on Virchow2 embeddings: Patch-level performance metrics NSCLC vs. MET.*

| Fold | Class | PRE | REC | F1 | BA | AUROC | MCC |
|---|---|---|---|---|---|---|---|
| Fold 1 | MET | 0.6253 | 0.3943 | 0.4837 | 0.6113 | 0.7140 | 0.2488 |
| | NSCLC | 0.6529 | 0.8282 | 0.7302 | | | |
| Fold 2 | MET | 0.6742 | 0.4373 | 0.5305 | 0.6418 | 0.7492 | 0.3143 |
| | NSCLC | 0.6741 | 0.8463 | 0.7505 | | | |
| Fold 3 | MET | 0.6604 | 0.4192 | 0.5129 | 0.6312 | 0.7182 | 0.2928 |
| | NSCLC | 0.6663 | 0.8432 | 0.7444 | | | |
| Fold 4 | MET | 0.6873 | 0.4493 | 0.5434 | 0.6503 | 0.7488 | 0.3324 |
| | NSCLC | 0.6801 | 0.8513 | 0.7562 | | | |
| Fold 5 | MET | 0.6616 | 0.4098 | 0.5061 | 0.6287 | 0.7305 | 0.2894 |
| | NSCLC | 0.6639 | 0.8476 | 0.7446 | | | |
| Fold 6 | MET | 0.7188 | 0.3754 | 0.4932 | 0.6343 | 0.7379 | 0.3202 |
| | NSCLC | 0.6630 | 0.8932 | 0.7610 | | | |
| Fold 7 | MET | 0.6631 | 0.4227 | 0.5163 | 0.6333 | 0.7277 | 0.2970 |
| | NSCLC | 0.6678 | 0.8438 | 0.7456 | | | |
| Fold 8 | MET | 0.6532 | 0.4158 | 0.5081 | 0.6276 | 0.7244 | 0.2846 |
| | NSCLC | 0.6640 | 0.8395 | 0.7415 | | | |
| Fold 9 | MET | 0.6316 | 0.4433 | 0.5209 | 0.6276 | 0.7226 | 0.2763 |
| | NSCLC | 0.6674 | 0.8120 | 0.7326 | | | |
| Fold 10 | MET | 0.6601 | 0.4304 | 0.5211 | 0.6346 | 0.7382 | 0.2979 |
| | NSCLC | 0.6695 | 0.8389 | 0.7447 | | | |
| **Mean ± std** | **MET** | 0.6635 ±0.0265 | 0.4198 ±0.0226 | 0.5136 ±0.0173 | 0.6321 ±0.0101 | 0.7311 ±0.0121 | 0.2954 ±0.0237 |
| | **NSCLC** | 0.6669 ±0.0072 | 0.8444 ±0.0206 | 0.7451 ±0.0094 | | | |

*Table S35: Logistic regression on Virchow2 embeddings: Slide-level performance metrics NSCLC vs. MET.*

| Fold | Class | PRE | REC | F1 | BA | AUROC | MCC |
|---|---|---|---|---|---|---|---|
| Fold 1 | MET | 0.7500 | 0.5455 | 0.6316 | 0.7424 | 0.7658 | 0.5443 |
| | NSCLC | 0.8611 | 0.9394 | 0.8986 | | | |
| Fold 2 | MET | 0.8000 | 0.3636 | 0.5000 | 0.6667 | 0.8760 | 0.4548 |
| | NSCLC | 0.8205 | 0.9697 | 0.8889 | | | |
| Fold 3 | MET | 1.0000 | 0.4545 | 0.6250 | 0.7273 | 0.8127 | 0.6202 |
| | NSCLC | 0.8462 | 1.0000 | 0.9167 | | | |
| Fold 4 | MET | 1.0000 | 0.6364 | 0.7778 | 0.8182 | 0.7961 | 0.7534 |
| | NSCLC | 0.8919 | 1.0000 | 0.9429 | | | |
| Fold 5 | MET | 0.8333 | 0.4545 | 0.5882 | 0.7121 | 0.8264 | 0.5353 |
| | NSCLC | 0.8421 | 0.9697 | 0.9014 | | | |
| Fold 6 | MET | 1.0000 | 0.4545 | 0.6250 | 0.7273 | 0.8512 | 0.6202 |
| | NSCLC | 0.8462 | 1.0000 | 0.9167 | | | |
| Fold 7 | MET | 1.0000 | 0.5455 | 0.7059 | 0.7727 | 0.8320 | 0.6882 |
| | NSCLC | 0.8684 | 1.0000 | 0.9296 | | | |
| Fold 8 | MET | 1.0000 | 0.5455 | 0.7059 | 0.7727 | 0.8402 | 0.6882 |
| | NSCLC | 0.8684 | 1.0000 | 0.9296 | | | |
| Fold 9 | MET | 1.0000 | 0.5455 | 0.7059 | 0.7727 | 0.8209 | 0.6882 |
| | NSCLC | 0.8684 | 1.0000 | 0.9296 | | | |
| Fold 10 | MET | 1.0000 | 0.5455 | 0.7059 | 0.7727 | 0.8237 | 0.6882 |
| | NSCLC | 0.8684 | 1.0000 | 0.9296 | | | |
| **Mean ± std** | **MET** | 0.9383 ±0.1012 | 0.5091 ±0.0767 | 0.6571 ±0.0790 | 0.7485 ±0.0424 | 0.8245 ±0.0300 | 0.6281 ±0.0919 |
| | **NSCLC** | 0.8582 ±0.0198 | 0.9879 ±0.0212 | 0.9183 ±0.0172 | | | |

*Table S36: Logistic regression on Virchow2 embeddings: Patient-level performance metrics NSCLC vs. MET.*

| Fold | Class | PRE | REC | F1 | BA | AUROC | MCC |
|---|---|---|---|---|---|---|---|
| Fold 1 | MET | 0.8000 | 0.5000 | 0.6154 | 0.7262 | 0.7857 | 0.5353 |
| | NSCLC | 0.8333 | 0.9524 | 0.8889 | | | |
| Fold 2 | MET | 1.0000 | 0.3750 | 0.5455 | 0.6875 | 0.9167 | 0.5503 |
| | NSCLC | 0.8077 | 1.0000 | 0.8936 | | | |
| Fold 3 | MET | 1.0000 | 0.3750 | 0.5455 | 0.6875 | 0.8393 | 0.5503 |
| | NSCLC | 0.8077 | 1.0000 | 0.8936 | | | |
| Fold 4 | MET | 1.0000 | 0.6250 | 0.7692 | 0.8125 | 0.8333 | 0.7395 |
| | NSCLC | 0.8750 | 1.0000 | 0.9333 | | | |
| Fold 5 | MET | 1.0000 | 0.3750 | 0.5455 | 0.6875 | 0.8571 | 0.5503 |
| | NSCLC | 0.8077 | 1.0000 | 0.8936 | | | |
| Fold 6 | MET | 1.0000 | 0.3750 | 0.5455 | 0.6875 | 0.8869 | 0.5503 |
| | NSCLC | 0.8077 | 1.0000 | 0.8936 | | | |
| Fold 7 | MET | 1.0000 | 0.5000 | 0.6667 | 0.7500 | 0.8452 | 0.6481 |
| | NSCLC | 0.8400 | 1.0000 | 0.9130 | | | |
| Fold 8 | MET | 1.0000 | 0.5000 | 0.6667 | 0.7500 | 0.8512 | 0.6481 |
| | NSCLC | 0.8400 | 1.0000 | 0.9130 | | | |
| Fold 9 | MET | 1.0000 | 0.5000 | 0.6667 | 0.7500 | 0.8274 | 0.6481 |
| | NSCLC | 0.8400 | 1.0000 | 0.9130 | | | |
| Fold 10 | MET | 1.0000 | 0.5000 | 0.6667 | 0.7500 | 0.8512 | 0.6481 |
| | NSCLC | 0.8400 | 1.0000 | 0.9130 | | | |
| **Mean ± std** | **MET** | 0.9800 ±0.0632 | 0.4625 ±0.0844 | 0.6233 ±0.0768 | 0.7289 ±0.0417 | 0.8494 ±0.0348 | 0.6068 ±0.0685 |
| | **NSCLC** | 0.8299 ±0.0222 | 0.9952 ±0.0151 | 0.9049 ±0.0143 | | | |

*Table S37: MLP: Performance metrics NSCLC vs. MET.*

| Architecture | Level | Class | PRE | REC | F1 | BA (95% CI) | AUROC (95% CI) | MCC (95% CI) |
|---|---|---|---|---|---|---|---|---|
| **ResNet50** | Patch | MET | 0.6369 | 0.5395 | 0.5842 | 0.6580 | 0.7206 | 0.3256 |
| | | NSCLC | 0.6987 | 0.7764 | 0.7355 | (0.6403-0.6761) | (0.7028-0.7394) | (0.2888-0.3630) |
| | Slide | MET | 0.5714 | 0.7273 | 0.6400 | 0.7727 | 0.8127 | 0.5071 |
| | | NSCLC | 0.9000 | 0.8182 | 0.8571 | (0.6041-0.9103) | (0.6323-0.9474) | (0.1992-0.7531) |
| | Patient | MET | 0.5556 | 0.6250 | 0.5882 | 0.7173 | 0.8155 | 0.4198 |
| | | NSCLC | 0.8500 | 0.8095 | 0.8293 | (0.4975-0.9091) | (0.6250-0.9638) | (-0.0052-0.7533) |
| **CONCH** | Patch | MET | 0.6201 | 0.4081 | 0.4922 | 0.6132 | 0.6824 | 0.2497 |
| | | NSCLC | 0.6553 | 0.8182 | 0.7278 | (0.5967-0.6299) | (0.6632-0.7016) | (0.2131-0.2847) |
| | Slide | MET | 0.5556 | 0.4545 | 0.5000 | 0.6667 | 0.7493 | 0.3578 |
| | | NSCLC | 0.8286 | 0.8788 | 0.8529 | (0.5024-0.8334) | (0.5651-0.9092) | (0.0053-0.6616) |
| | Patient | MET | 0.5000 | 0.3750 | 0.4286 | 0.6161 | 0.7440 | 0.2561 |
| | | NSCLC | 0.7826 | 0.8571 | 0.8182 | (0.4259-0.8150) | (0.5315-0.9400) | (-0.1600-0.6282) |
| **CTransPath** | Patch | MET | 0.7409 | 0.3660 | 0.4899 | 0.6365 | 0.7554 | 0.3320 |
| | | NSCLC | 0.6630 | 0.9069 | 0.7660 | (0.6205-0.6536) | (0.7364-0.7738) | (0.2966-0.3689) |
| | Slide | MET | 0.7143 | 0.4545 | 0.5556 | 0.6970 | 0.8072 | 0.4664 |
| | | NSCLC | 0.8378 | 0.9394 | 0.8857 | (0.5508-0.8572) | (0.6525-0.9410) | (0.1269-0.7534) |
| | Patient | MET | 0.7500 | 0.3750 | 0.5000 | 0.6637 | 0.8274 | 0.4243 |
| | | NSCLC | 0.8000 | 0.9524 | 0.8696 | (0.4808-0.8732) | (0.6521-0.9744) | (-0.0534-0.7583) |
| **H-optimus-0** | Patch | MET | 0.6989 | 0.2672 | 0.3866 | 0.5917 | 0.6859 | 0.2465 |
| | | NSCLC | 0.6323 | 0.9163 | 0.7483 | (0.5781-0.6066) | (0.6657-0.7058) | (0.2111-0.2838) |
| | Slide | MET | 0.5714 | 0.3636 | 0.4444 | 0.6364 | 0.6942 | 0.3229 |
| | | NSCLC | 0.8108 | 0.9091 | 0.8571 | (0.4780-0.7912) | (0.4768-0.9035) | (-0.0443-0.6367) |
| | Patient | MET | 0.6667 | 0.2500 | 0.3636 | 0.6012 | 0.6845 | 0.2970 |
| | | NSCLC | 0.7692 | 0.9524 | 0.8511 | (0.4583-0.7793) | (0.4156-0.9334) | (-0.1359-0.6683) |
| **H-optimus-1** | Patch | MET | 0.7089 | 0.4708 | 0.5658 | 0.6651 | 0.7566 | 0.3633 |
| | | NSCLC | 0.6908 | 0.8595 | 0.7659 | (0.6495-0.6817) | (0.7391-0.7749) | (0.3304-0.3983) |
| | Slide | MET | 0.5556 | 0.4545 | 0.5000 | 0.6667 | 0.8209 | 0.3578 |
| | | NSCLC | 0.8286 | 0.8788 | 0.8529 | (0.5111-0.8206) | (0.6690-0.9559) | (0.0222-0.6616) |
| | Patient | MET | 0.7500 | 0.3750 | 0.5000 | 0.6637 | 0.8452 | 0.4243 |
| | | NSCLC | 0.8000 | 0.9524 | 0.8696 | (0.4792-0.8737) | (0.6520-1.0000) | (-0.0777-0.7832) |
| **hibou-L** | Patch | MET | 0.7022 | 0.3849 | 0.4972 | 0.6331 | 0.7423 | 0.3120 |
| | | NSCLC | 0.6634 | 0.8813 | 0.7570 | (0.6170-0.6488) | (0.7231-0.7606) | (0.2762-0.3485) |
| | Slide | MET | 0.8333 | 0.4545 | 0.5882 | 0.7121 | 0.8264 | 0.5353 |
| | | NSCLC | 0.8421 | 0.9697 | 0.9014 | (0.5677-0.8746) | (0.6588-0.9531) | (0.2057-0.8142) |
| | Patient | MET | 0.7500 | 0.3750 | 0.5000 | 0.6637 | 0.8274 | 0.4243 |
| | | NSCLC | 0.8000 | 0.9524 | 0.8696 | (0.4805-0.8641) | (0.6377-0.9805) | (-0.0643-0.7832) |
| **Kaiko** | Patch | MET | 0.6957 | 0.4596 | 0.5535 | 0.6567 | 0.7582 | 0.3454 |
| | | NSCLC | 0.6849 | 0.8538 | 0.7601 | (0.6397-0.6726) | (0.7393-0.7744) | (0.3078-0.3807) |
| | Slide | MET | 0.5000 | 0.5455 | 0.5217 | 0.6818 | 0.7466 | 0.3536 |
| | | NSCLC | 0.8438 | 0.8182 | 0.8308 | (0.5126-0.8475) | (0.5521-0.9118) | (0.0256-0.6423) |
| | Patient | MET | 0.5714 | 0.5000 | 0.5333 | 0.6786 | 0.7679 | 0.3730 |
| | | NSCLC | 0.8182 | 0.8571 | 0.8372 | (0.4750-0.8814) | (0.5416-0.9501) | (-0.0517-0.7336) |
| **Phikon** | Patch | MET | 0.7120 | 0.3780 | 0.4938 | 0.6334 | 0.7432 | 0.3162 |
| | | NSCLC | 0.6628 | 0.8888 | 0.7593 | (0.6173-0.6485) | (0.7247-0.7601) | (0.2786-0.3505) |
| | Slide | MET | 0.6667 | 0.3636 | 0.4706 | 0.6515 | 0.8154 | 0.3824 |
| | | NSCLC | 0.8158 | 0.9394 | 0.8732 | (0.4999-0.8031) | (0.6437-0.9460) | (-0.0002-0.6806) |
| | Patient | MET | 0.6667 | 0.2500 | 0.3636 | 0.6012 | 0.8571 | 0.2970 |
| | | NSCLC | 0.7692 | 0.9524 | 0.8511 | (0.4583-0.7793) | (0.6616-0.9935) | (-0.1359-0.6683) |
| **ProvGigaPath** | Patch | MET | 0.6922 | 0.3265 | 0.4437 | 0.6105 | 0.7213 | 0.2734 |
| | | NSCLC | 0.6462 | 0.8944 | 0.7503 | (0.5951-0.6257) | (0.7024-0.7401) | (0.2377-0.3087) |
| | Slide | MET | 0.4444 | 0.3636 | 0.4000 | 0.6061 | 0.7576 | 0.2277 |
| | | NSCLC | 0.8000 | 0.8485 | 0.8235 | (0.4510-0.7618) | (0.5857-0.9082) | (-0.1047-0.5636) |
| | Patient | MET | 0.6667 | 0.2500 | 0.3636 | 0.6012 | 0.7917 | 0.2970 |
| | | NSCLC | 0.7692 | 0.9524 | 0.8511 | (0.4583-0.7793) | (0.5844-0.9465) | (-0.1359-0.6683) |
| **TissueConcepts** | Patch | MET | 0.6671 | 0.4734 | 0.5538 | 0.6508 | 0.7102 | 0.3253 |
| | | NSCLC | 0.6839 | 0.8282 | 0.7492 | (0.6345-0.6682) | (0.6895-0.7301) | (0.2900-0.3610) |
| | Slide | MET | 0.8333 | 0.4545 | 0.5882 | 0.7121 | 0.8815 | 0.5353 |
| | | NSCLC | 0.8421 | 0.9697 | 0.9014 | (0.5706-0.8636) | (0.7429-0.9846) | (0.2293-0.8020) |
| | Patient | MET | 0.7500 | 0.3750 | 0.5000 | 0.6637 | 0.9286 | 0.4243 |
| | | NSCLC | 0.8000 | 0.9524 | 0.8696 | (0.4792-0.8732) | (0.7986-1.0000) | (-0.0757-0.7901) |
| **UNI2-h** | Patch | MET | 0.7524 | 0.3419 | 0.4702 | 0.6301 | 0.7522 | 0.3265 |
| | | NSCLC | 0.6574 | 0.9182 | 0.7662 | (0.6149-0.6445) | (0.7343-0.7695) | (0.2911-0.3595) |
| | Slide | MET | 0.6667 | 0.3636 | 0.4706 | 0.6515 | 0.7989 | 0.3824 |
| | | NSCLC | 0.8158 | 0.9394 | 0.8732 | (0.5059-0.8030) | (0.6382-0.9421) | (0.0170-0.6719) |
| | Patient | MET | 0.6667 | 0.2500 | 0.3636 | 0.6012 | 0.8571 | 0.2970 |
| | | NSCLC | 0.7692 | 0.9524 | 0.8511 | (0.4565-0.7857) | (0.6736-0.9872) | (-0.1390-0.6651) |
| **Virchow2** | Patch | MET | 0.6736 | 0.4450 | 0.5360 | 0.6441 | 0.7432 | 0.3176 |
| | | NSCLC | 0.6764 | 0.8432 | 0.7506 | (0.6258-0.6620) | (0.7247-0.7613) | (0.2767-0.3538) |
| | Slide | MET | 0.8571 | 0.5455 | 0.6667 | 0.7576 | 0.7824 | 0.6099 |
| | | NSCLC | 0.8649 | 0.9697 | 0.9143 | (0.6095-0.9167) | (0.5999-0.9429) | (0.3185-0.8605) |
| | Patient | MET | 1.0000 | 0.5000 | 0.6667 | 0.7500 | 0.7976 | 0.6481 |
| | | NSCLC | 0.8400 | 1.0000 | 0.9130 | (0.5712-0.9444) | (0.5667-0.9855) | (0.3343-0.9201) |

## 4. Performance metrics tables AC vs. SCC

*Table S38: Logistic regression on CONCH embeddings: Patch-level performance metrics AC vs. SCC.*

| Fold | Class | PRE | REC | F1 | BA | AUROC | MCC |
|---|---|---|---|---|---|---|---|
| Fold 1 | AC | 0.4516 | 0.3526 | 0.3960 | 0.5330 | 0.5673 | 0.0697 |
| | SCC | 0.6221 | 0.7133 | 0.6646 | | | |
| Fold 2 | AC | 0.4403 | 0.3526 | 0.3916 | 0.5262 | 0.5813 | 0.0551 |
| | SCC | 0.6176 | 0.6998 | 0.6561 | | | |
| Fold 3 | AC | 0.4162 | 0.3501 | 0.3803 | 0.5106 | 0.5382 | 0.0221 |
| | SCC | 0.6067 | 0.6712 | 0.6373 | | | |
| Fold 4 | AC | 0.4406 | 0.3552 | 0.3933 | 0.5267 | 0.5460 | 0.0559 |
| | SCC | 0.6179 | 0.6981 | 0.6556 | | | |
| Fold 5 | AC | 0.4361 | 0.3955 | 0.4148 | 0.5266 | 0.5503 | 0.0541 |
| | SCC | 0.6190 | 0.6577 | 0.6378 | | | |
| Fold 6 | AC | 0.4695 | 0.3879 | 0.4248 | 0.5472 | 0.5749 | 0.0984 |
| | SCC | 0.6329 | 0.7066 | 0.6677 | | | |
| Fold 7 | AC | 0.4702 | 0.3980 | 0.4311 | 0.5489 | 0.5869 | 0.1012 |
| | SCC | 0.6346 | 0.6998 | 0.6656 | | | |
| Fold 8 | AC | 0.4433 | 0.4232 | 0.4330 | 0.5337 | 0.5626 | 0.0679 |
| | SCC | 0.6252 | 0.6442 | 0.6346 | | | |
| Fold 9 | AC | 0.4380 | 0.4181 | 0.4278 | 0.5295 | 0.5644 | 0.0594 |
| | SCC | 0.6219 | 0.6408 | 0.6312 | | | |
| Fold 10 | AC | 0.3853 | 0.3174 | 0.3481 | 0.4892 | 0.5138 | -0.0225 |
| | SCC | 0.5913 | 0.6610 | 0.6242 | | | |
| **Mean ± std** | **AC** | 0.4391 ±0.0247 | 0.3751 ±0.0344 | 0.4041 ±0.0273 | 0.5272 ±0.0172 | 0.5586 ±0.0220 | 0.0561 ±0.0357 |
| | **SCC** | 0.6189 ±0.0125 | 0.6793 ±0.0272 | 0.6475 ±0.0161 | | | |

*Table S39: Logistic regression on CONCH embeddings: Slide-level performance metrics AC vs. SCC.*

| Fold | Class | PRE | REC | F1 | BA | AUROC | MCC |
|---|---|---|---|---|---|---|---|
| Fold 1 | AC | 0.5000 | 0.5000 | 0.5000 | 0.5833 | 0.6481 | 0.1667 |
| | SCC | 0.6667 | 0.6667 | 0.6667 | | | |
| Fold 2 | AC | 0.4545 | 0.4167 | 0.4348 | 0.5417 | 0.6528 | 0.0847 |
| | SCC | 0.6316 | 0.6667 | 0.6486 | | | |
| Fold 3 | AC | 0.4545 | 0.4167 | 0.4348 | 0.5417 | 0.6111 | 0.0847 |
| | SCC | 0.6316 | 0.6667 | 0.6486 | | | |
| Fold 4 | AC | 0.4545 | 0.4167 | 0.4348 | 0.5417 | 0.5787 | 0.0847 |
| | SCC | 0.6316 | 0.6667 | 0.6486 | | | |
| Fold 5 | AC | 0.5385 | 0.5833 | 0.5600 | 0.6250 | 0.6343 | 0.2472 |
| | SCC | 0.7059 | 0.6667 | 0.6857 | | | |
| Fold 6 | AC | 0.5385 | 0.5833 | 0.5600 | 0.6250 | 0.5880 | 0.2472 |
| | SCC | 0.7059 | 0.6667 | 0.6857 | | | |
| Fold 7 | AC | 0.5000 | 0.5000 | 0.5000 | 0.5833 | 0.6435 | 0.1667 |
| | SCC | 0.6667 | 0.6667 | 0.6667 | | | |
| Fold 8 | AC | 0.5385 | 0.5833 | 0.5600 | 0.6250 | 0.6528 | 0.2472 |
| | SCC | 0.7059 | 0.6667 | 0.6857 | | | |
| Fold 9 | AC | 0.5000 | 0.5000 | 0.5000 | 0.5833 | 0.6574 | 0.1667 |
| | SCC | 0.6667 | 0.6667 | 0.6667 | | | |
| Fold 10 | AC | 0.4545 | 0.4167 | 0.4348 | 0.5417 | 0.6019 | 0.0847 |
| | SCC | 0.6316 | 0.6667 | 0.6486 | | | |
| **Mean ± std** | **AC** | 0.4934 ±0.0369 | 0.4917 ±0.0730 | 0.4919 ±0.0549 | 0.5792 ±0.0365 | 0.6269 ±0.0294 | 0.1580 ±0.0711 |
| | **SCC** | 0.6644 ±0.0325 | 0.6667 ±0.0000 | 0.6652 ±0.0162 | | | |

*Table S40: Logistic regression on CONCH embeddings: Patient-level performance metrics AC vs. SCC.*

| Fold | Class | PRE | REC | F1 | BA | AUROC | MCC |
|---|---|---|---|---|---|---|---|
| Fold 1 | AC | 0.5000 | 0.5000 | 0.5000 | 0.5682 | 0.6705 | 0.1364 |
| | SCC | 0.6364 | 0.6364 | 0.6364 | | | |
| Fold 2 | AC | 0.4286 | 0.3750 | 0.4000 | 0.5057 | 0.6932 | 0.0116 |
| | SCC | 0.5833 | 0.6364 | 0.6087 | | | |
| Fold 3 | AC | 0.4286 | 0.3750 | 0.4000 | 0.5057 | 0.6364 | 0.0116 |
| | SCC | 0.5833 | 0.6364 | 0.6087 | | | |
| Fold 4 | AC | 0.4286 | 0.3750 | 0.4000 | 0.5057 | 0.6250 | 0.0116 |
| | SCC | 0.5833 | 0.6364 | 0.6087 | | | |
| Fold 5 | AC | 0.5556 | 0.6250 | 0.5882 | 0.6307 | 0.6818 | 0.2584 |
| | SCC | 0.7000 | 0.6364 | 0.6667 | | | |
| Fold 6 | AC | 0.5556 | 0.6250 | 0.5882 | 0.6307 | 0.6250 | 0.2584 |
| | SCC | 0.7000 | 0.6364 | 0.6667 | | | |
| Fold 7 | AC | 0.5000 | 0.5000 | 0.5000 | 0.5682 | 0.6932 | 0.1364 |
| | SCC | 0.6364 | 0.6364 | 0.6364 | | | |
| Fold 8 | AC | 0.5556 | 0.6250 | 0.5882 | 0.6307 | 0.6705 | 0.2584 |
| | SCC | 0.7000 | 0.6364 | 0.6667 | | | |
| Fold 9 | AC | 0.5000 | 0.5000 | 0.5000 | 0.5682 | 0.6477 | 0.1364 |
| | SCC | 0.6364 | 0.6364 | 0.6364 | | | |
| Fold 10 | AC | 0.4286 | 0.3750 | 0.4000 | 0.5057 | 0.6023 | 0.0116 |
| | SCC | 0.5833 | 0.6364 | 0.6087 | | | |
| **Mean ± std** | **AC** | 0.4881 ±0.0560 | 0.4875 ±0.1094 | 0.4865 ±0.0827 | 0.5619 ±0.0547 | 0.6545 ±0.0318 | 0.1231 ±0.1081 |
| | **SCC** | 0.6342 ±0.0509 | 0.6364 ±0.0000 | 0.6344 ±0.0253 | | | |

*Table S41: Logistic regression on CTransPath embeddings: Patch-level performance metrics AC vs. SCC.*

| Fold | Class | PRE | REC | F1 | BA | AUROC | MCC |
|---|---|---|---|---|---|---|---|
| Fold 1 | AC | 0.5078 | 0.4081 | 0.4525 | 0.5717 | 0.5977 | 0.1503 |
| | SCC | 0.6498 | 0.7352 | 0.6899 | | | |
| Fold 2 | AC | 0.4897 | 0.4207 | 0.4526 | 0.5636 | 0.6118 | 0.1312 |
| | SCC | 0.6456 | 0.7066 | 0.6747 | | | |
| Fold 3 | AC | 0.4573 | 0.3778 | 0.4138 | 0.5388 | 0.5751 | 0.0809 |
| | SCC | 0.6269 | 0.6998 | 0.6614 | | | |
| Fold 4 | AC | 0.4717 | 0.3778 | 0.4196 | 0.5473 | 0.5718 | 0.0992 |
| | SCC | 0.6324 | 0.7167 | 0.6719 | | | |
| Fold 5 | AC | 0.4727 | 0.4584 | 0.4655 | 0.5581 | 0.5879 | 0.1167 |
| | SCC | 0.6446 | 0.6577 | 0.6511 | | | |
| Fold 6 | AC | 0.4896 | 0.4156 | 0.4496 | 0.5628 | 0.5716 | 0.1299 |
| | SCC | 0.6447 | 0.7099 | 0.6758 | | | |
| Fold 7 | AC | 0.4972 | 0.4484 | 0.4715 | 0.5724 | 0.6029 | 0.1477 |
| | SCC | 0.6535 | 0.6965 | 0.6743 | | | |
| Fold 8 | AC | 0.4948 | 0.4811 | 0.4879 | 0.5761 | 0.5975 | 0.1530 |
| | SCC | 0.6589 | 0.6712 | 0.6650 | | | |
| Fold 9 | AC | 0.4882 | 0.4685 | 0.4781 | 0.5698 | 0.6278 | 0.1407 |
| | SCC | 0.6535 | 0.6712 | 0.6622 | | | |
| Fold 10 | AC | 0.4638 | 0.4358 | 0.4494 | 0.5493 | 0.5759 | 0.0996 |
| | SCC | 0.6370 | 0.6627 | 0.6496 | | | |
| **Mean ± std** | **AC** | 0.4833 ±0.0161 | 0.4292 ±0.0357 | 0.4540 ±0.0236 | 0.5610 ±0.0124 | 0.5920 ±0.0190 | 0.1249 ±0.0249 |
| | **SCC** | 0.6447 ±0.0101 | 0.6927 ±0.0258 | 0.6676 ±0.0122 | | | |

*Table S42: Logistic regression on CTransPath embeddings: Slide-level performance metrics AC vs. SCC.*

| Fold | Class | PRE | REC | F1 | BA | AUROC | MCC |
|---|---|---|---|---|---|---|---|
| Fold 1 | AC | 0.5000 | 0.5833 | 0.5385 | 0.5972 | 0.5880 | 0.1909 |
| | SCC | 0.6875 | 0.6111 | 0.6471 | | | |
| Fold 2 | AC | 0.5000 | 0.6667 | 0.5714 | 0.6111 | 0.5926 | 0.2182 |
| | SCC | 0.7143 | 0.5556 | 0.6250 | | | |
| Fold 3 | AC | 0.4000 | 0.5000 | 0.4444 | 0.5000 | 0.5694 | 0.0000 |
| | SCC | 0.6000 | 0.5000 | 0.5455 | | | |
| Fold 4 | AC | 0.4286 | 0.5000 | 0.4615 | 0.5278 | 0.5139 | 0.0546 |
| | SCC | 0.6250 | 0.5556 | 0.5882 | | | |
| Fold 5 | AC | 0.4118 | 0.5833 | 0.4828 | 0.5139 | 0.5787 | 0.0275 |
| | SCC | 0.6154 | 0.4444 | 0.5161 | | | |
| Fold 6 | AC | 0.4667 | 0.5833 | 0.5185 | 0.5694 | 0.5370 | 0.1361 |
| | SCC | 0.6667 | 0.5556 | 0.6061 | | | |
| Fold 7 | AC | 0.4118 | 0.5833 | 0.4828 | 0.5139 | 0.5972 | 0.0275 |
| | SCC | 0.6154 | 0.4444 | 0.5161 | | | |
| Fold 8 | AC | 0.4706 | 0.6667 | 0.5517 | 0.5833 | 0.5833 | 0.1648 |
| | SCC | 0.6923 | 0.5000 | 0.5806 | | | |
| Fold 9 | AC | 0.4375 | 0.5833 | 0.5000 | 0.5417 | 0.5972 | 0.0818 |
| | SCC | 0.6429 | 0.5000 | 0.5625 | | | |
| Fold 10 | AC | 0.3750 | 0.5000 | 0.4286 | 0.4722 | 0.5370 | -0.0546 |
| | SCC | 0.5714 | 0.4444 | 0.5000 | | | |
| **Mean ± std** | **AC** | 0.4402 ±0.0427 | 0.5750 ±0.0615 | 0.4980 ±0.0469 | 0.5431 ±0.0456 | 0.5694 ±0.0296 | 0.0847 ±0.0896 |
| | **SCC** | 0.6431 ±0.0458 | 0.5111 ±0.0574 | 0.5687 ±0.0495 | | | |

*Table S43: Logistic regression on CTransPath embeddings: Patient-level performance metrics AC vs. SCC.*

| Fold | Class | PRE | REC | F1 | BA | AUROC | MCC |
|---|---|---|---|---|---|---|---|
| Fold 1 | AC | 0.5556 | 0.6250 | 0.5882 | 0.6307 | 0.6705 | 0.2584 |
| | SCC | 0.7000 | 0.6364 | 0.6667 | | | |
| Fold 2 | AC | 0.6000 | 0.7500 | 0.6667 | 0.6932 | 0.6818 | 0.3820 |
| | SCC | 0.7778 | 0.6364 | 0.7000 | | | |
| Fold 3 | AC | 0.5000 | 0.5000 | 0.5000 | 0.5682 | 0.6250 | 0.1364 |
| | SCC | 0.6364 | 0.6364 | 0.6364 | | | |
| Fold 4 | AC | 0.5000 | 0.5000 | 0.5000 | 0.5682 | 0.5682 | 0.1364 |
| | SCC | 0.6364 | 0.6364 | 0.6364 | | | |
| Fold 5 | AC | 0.5000 | 0.6250 | 0.5556 | 0.5852 | 0.6023 | 0.1685 |
| | SCC | 0.6667 | 0.5455 | 0.6000 | | | |
| Fold 6 | AC | 0.5556 | 0.6250 | 0.5882 | 0.6307 | 0.5909 | 0.2584 |
| | SCC | 0.7000 | 0.6364 | 0.6667 | | | |
| Fold 7 | AC | 0.5000 | 0.6250 | 0.5556 | 0.5852 | 0.6818 | 0.1685 |
| | SCC | 0.6667 | 0.5455 | 0.6000 | | | |
| Fold 8 | AC | 0.5455 | 0.7500 | 0.6316 | 0.6477 | 0.6818 | 0.2955 |
| | SCC | 0.7500 | 0.5455 | 0.6316 | | | |
| Fold 9 | AC | 0.5556 | 0.6250 | 0.5882 | 0.6307 | 0.6477 | 0.2584 |
| | SCC | 0.7000 | 0.6364 | 0.6667 | | | |
| Fold 10 | AC | 0.4000 | 0.5000 | 0.4444 | 0.4773 | 0.6023 | -0.0449 |
| | SCC | 0.5556 | 0.4545 | 0.5000 | | | |
| **Mean ± std** | **AC** | 0.5212 ±0.0546 | 0.6125 ±0.0922 | 0.5619 ±0.0662 | 0.6017 ±0.0590 | 0.6352 ±0.0430 | 0.2018 ±0.1167 |
| | **SCC** | 0.6789 ±0.0626 | 0.5909 ±0.0643 | 0.6304 ±0.0555 | | | |

*Table S44: Logistic regression on H-optimus-0 embeddings: Patch-level performance metrics AC vs. SCC.*

| Fold | Class | PRE | REC | F1 | BA | AUROC | MCC |
|---|---|---|---|---|---|---|---|
| Fold 1 | AC | 0.5503 | 0.4131 | 0.4719 | 0.5936 | 0.6802 | 0.1999 |
| | SCC | 0.6633 | 0.7740 | 0.7144 | | | |
| Fold 2 | AC | 0.5813 | 0.4232 | 0.4898 | 0.6096 | 0.6918 | 0.2362 |
| | SCC | 0.6733 | 0.7960 | 0.7295 | | | |
| Fold 3 | AC | 0.5259 | 0.3577 | 0.4258 | 0.5709 | 0.6401 | 0.1561 |
| | SCC | 0.6458 | 0.7841 | 0.7083 | | | |
| Fold 4 | AC | 0.5382 | 0.3552 | 0.4279 | 0.5756 | 0.6354 | 0.1679 |
| | SCC | 0.6484 | 0.7960 | 0.7146 | | | |
| Fold 5 | AC | 0.5499 | 0.4861 | 0.5160 | 0.6099 | 0.6776 | 0.2251 |
| | SCC | 0.6808 | 0.7336 | 0.7062 | | | |
| Fold 6 | AC | 0.5479 | 0.4181 | 0.4743 | 0.5936 | 0.6461 | 0.1990 |
| | SCC | 0.6638 | 0.7690 | 0.7125 | | | |
| Fold 7 | AC | 0.5494 | 0.4484 | 0.4938 | 0.6011 | 0.6702 | 0.2112 |
| | SCC | 0.6712 | 0.7538 | 0.7101 | | | |
| Fold 8 | AC | 0.5167 | 0.3904 | 0.4448 | 0.5730 | 0.6496 | 0.1556 |
| | SCC | 0.6493 | 0.7555 | 0.6984 | | | |
| Fold 9 | AC | 0.5402 | 0.4736 | 0.5047 | 0.6019 | 0.6644 | 0.2091 |
| | SCC | 0.6745 | 0.7302 | 0.7012 | | | |
| Fold 10 | AC | 0.5130 | 0.3980 | 0.4482 | 0.5725 | 0.6317 | 0.1535 |
| | SCC | 0.6496 | 0.7470 | 0.6949 | | | |
| **Mean ± std** | **AC** | 0.5413 ±0.0198 | 0.4164 ±0.0440 | 0.4697 ±0.0319 | 0.5901 ±0.0158 | 0.6587 ±0.0209 | 0.1914 ±0.0307 |
| | **SCC** | 0.6620 ±0.0129 | 0.7639 ±0.0238 | 0.7090 ±0.0099 | | | |

*Table S45: Logistic regression on H-optimus-0 embeddings: Slide-level performance metrics AC vs. SCC.*

| Fold | Class | PRE | REC | F1 | BA | AUROC | MCC |
|---|---|---|---|---|---|---|---|
| Fold 1 | AC | 0.5833 | 0.5833 | 0.5833 | 0.6528 | 0.7083 | 0.3056 |
| | SCC | 0.7222 | 0.7222 | 0.7222 | | | |
| Fold 2 | AC | 0.6667 | 0.6667 | 0.6667 | 0.7222 | 0.7315 | 0.4444 |
| | SCC | 0.7778 | 0.7778 | 0.7778 | | | |
| Fold 3 | AC | 0.6000 | 0.5000 | 0.5455 | 0.6389 | 0.6574 | 0.2887 |
| | SCC | 0.7000 | 0.7778 | 0.7368 | | | |
| Fold 4 | AC | 0.5455 | 0.5000 | 0.5217 | 0.6111 | 0.6481 | 0.2259 |
| | SCC | 0.6842 | 0.7222 | 0.7027 | | | |
| Fold 5 | AC | 0.6154 | 0.6667 | 0.6400 | 0.6944 | 0.7269 | 0.3845 |
| | SCC | 0.7647 | 0.7222 | 0.7429 | | | |
| Fold 6 | AC | 0.5455 | 0.5000 | 0.5217 | 0.6111 | 0.6389 | 0.2259 |
| | SCC | 0.6842 | 0.7222 | 0.7027 | | | |
| Fold 7 | AC | 0.6364 | 0.5833 | 0.6087 | 0.6806 | 0.6620 | 0.3671 |
| | SCC | 0.7368 | 0.7778 | 0.7568 | | | |
| Fold 8 | AC | 0.5385 | 0.5833 | 0.5600 | 0.6250 | 0.6389 | 0.2472 |
| | SCC | 0.7059 | 0.6667 | 0.6857 | | | |
| Fold 9 | AC | 0.5385 | 0.5833 | 0.5600 | 0.6250 | 0.7037 | 0.2472 |
| | SCC | 0.7059 | 0.6667 | 0.6857 | | | |
| Fold 10 | AC | 0.5455 | 0.5000 | 0.5217 | 0.6111 | 0.6944 | 0.2259 |
| | SCC | 0.6842 | 0.7222 | 0.7027 | | | |
| **Mean ± std** | **AC** | 0.5815 ±0.0464 | 0.5667 ±0.0657 | 0.5729 ±0.0512 | 0.6472 ±0.0394 | 0.6810 ±0.0360 | 0.2962 ±0.0779 |
| | **SCC** | 0.7166 ±0.0336 | 0.7278 ±0.0410 | 0.7216 ±0.0311 | | | |

*Table S46: Logistic regression on H-optimus-0 embeddings: Patient-level performance metrics AC vs. SCC.*

| Fold | Class | PRE | REC | F1 | BA | AUROC | MCC |
|---|---|---|---|---|---|---|---|
| Fold 1 | AC | 0.6250 | 0.6250 | 0.6250 | 0.6761 | 0.7500 | 0.3523 |
| | SCC | 0.7273 | 0.7273 | 0.7273 | | | |
| Fold 2 | AC | 0.7500 | 0.7500 | 0.7500 | 0.7841 | 0.7955 | 0.5682 |
| | SCC | 0.8182 | 0.8182 | 0.8182 | | | |
| Fold 3 | AC | 0.7143 | 0.6250 | 0.6667 | 0.7216 | 0.7273 | 0.4536 |
| | SCC | 0.7500 | 0.8182 | 0.7826 | | | |
| Fold 4 | AC | 0.5714 | 0.5000 | 0.5333 | 0.6136 | 0.6818 | 0.2326 |
| | SCC | 0.6667 | 0.7273 | 0.6957 | | | |
| Fold 5 | AC | 0.6667 | 0.7500 | 0.7059 | 0.7386 | 0.7614 | 0.4719 |
| | SCC | 0.8000 | 0.7273 | 0.7619 | | | |
| Fold 6 | AC | 0.5714 | 0.5000 | 0.5333 | 0.6136 | 0.6932 | 0.2326 |
| | SCC | 0.6667 | 0.7273 | 0.6957 | | | |
| Fold 7 | AC | 0.7500 | 0.7500 | 0.7500 | 0.7841 | 0.7614 | 0.5682 |
| | SCC | 0.8182 | 0.8182 | 0.8182 | | | |
| Fold 8 | AC | 0.6250 | 0.6250 | 0.6250 | 0.6761 | 0.7045 | 0.3523 |
| | SCC | 0.7273 | 0.7273 | 0.7273 | | | |
| Fold 9 | AC | 0.5556 | 0.6250 | 0.5882 | 0.6307 | 0.7500 | 0.2584 |
| | SCC | 0.7000 | 0.6364 | 0.6667 | | | |
| Fold 10 | AC | 0.5714 | 0.5000 | 0.5333 | 0.6136 | 0.7159 | 0.2326 |
| | SCC | 0.6667 | 0.7273 | 0.6957 | | | |
| **Mean ± std** | **AC** | 0.6401 ±0.0761 | 0.6250 ±0.1021 | 0.6311 ±0.0855 | 0.6852 ±0.0685 | 0.7341 ±0.0356 | 0.3723 ±0.1356 |
| | **SCC** | 0.7341 ±0.0611 | 0.7455 ±0.0575 | 0.7389 ±0.0539 | | | |

*Table S47: Logistic regression on H-optimus-1 embeddings: Patch-level performance metrics AC vs. SCC.*

| Fold | Class | PRE | REC | F1 | BA | AUROC | MCC |
|---|---|---|---|---|---|---|---|
| Fold 1 | AC | 0.5665 | 0.4509 | 0.5021 | 0.6099 | 0.6889 | 0.2311 |
| | SCC | 0.6766 | 0.7690 | 0.7198 | | | |
| Fold 2 | AC | 0.5733 | 0.4433 | 0.5000 | 0.6112 | 0.6848 | 0.2357 |
| | SCC | 0.6764 | 0.7791 | 0.7241 | | | |
| Fold 3 | AC | 0.5220 | 0.4181 | 0.4643 | 0.5809 | 0.6334 | 0.1698 |
| | SCC | 0.6562 | 0.7437 | 0.6972 | | | |
| Fold 4 | AC | 0.5402 | 0.3552 | 0.4286 | 0.5764 | 0.6393 | 0.1700 |
| | SCC | 0.6488 | 0.7976 | 0.7156 | | | |
| Fold 5 | AC | 0.5259 | 0.5113 | 0.5185 | 0.6014 | 0.6548 | 0.2037 |
| | SCC | 0.6788 | 0.6914 | 0.6850 | | | |
| Fold 6 | AC | 0.5813 | 0.4685 | 0.5188 | 0.6213 | 0.6753 | 0.2542 |
| | SCC | 0.6851 | 0.7740 | 0.7268 | | | |
| Fold 7 | AC | 0.5417 | 0.4912 | 0.5152 | 0.6065 | 0.6763 | 0.2169 |
| | SCC | 0.6794 | 0.7218 | 0.6999 | | | |
| Fold 8 | AC | 0.5428 | 0.4635 | 0.5000 | 0.6010 | 0.6622 | 0.2087 |
| | SCC | 0.6728 | 0.7386 | 0.7042 | | | |
| Fold 9 | AC | 0.5889 | 0.5592 | 0.5736 | 0.6489 | 0.6965 | 0.3006 |
| | SCC | 0.7145 | 0.7386 | 0.7264 | | | |
| Fold 10 | AC | 0.5077 | 0.4156 | 0.4571 | 0.5729 | 0.6371 | 0.1522 |
| | SCC | 0.6511 | 0.7302 | 0.6884 | | | |
| **Mean ± std** | **AC** | 0.5490 ±0.0272 | 0.4577 ±0.0563 | 0.4978 ±0.0402 | 0.6030 ±0.0228 | 0.6649 ±0.0229 | 0.2143 ±0.0444 |
| | **SCC** | 0.6740 ±0.0192 | 0.7484 ±0.0315 | 0.7088 ±0.0158 | | | |

*Table S48: Logistic regression on H-optimus-1 embeddings: Slide-level performance metrics AC vs. SCC.*

| Fold | Class | PRE | REC | F1 | BA | AUROC | MCC |
|---|---|---|---|---|---|---|---|
| Fold 1 | AC | 0.5556 | 0.4167 | 0.4762 | 0.5972 | 0.6852 | 0.2079 |
| | SCC | 0.6667 | 0.7778 | 0.7179 | | | |
| Fold 2 | AC | 0.5000 | 0.4167 | 0.4545 | 0.5694 | 0.6991 | 0.1443 |
| | SCC | 0.6500 | 0.7222 | 0.6842 | | | |
| Fold 3 | AC | 0.4444 | 0.3333 | 0.3810 | 0.5278 | 0.6481 | 0.0594 |
| | SCC | 0.6190 | 0.7222 | 0.6667 | | | |
| Fold 4 | AC | 0.4000 | 0.3333 | 0.3636 | 0.5000 | 0.5972 | 0.0000 |
| | SCC | 0.6000 | 0.6667 | 0.6316 | | | |
| Fold 5 | AC | 0.5000 | 0.5000 | 0.5000 | 0.5833 | 0.6898 | 0.1667 |
| | SCC | 0.6667 | 0.6667 | 0.6667 | | | |
| Fold 6 | AC | 0.6364 | 0.5833 | 0.6087 | 0.6806 | 0.6713 | 0.3671 |
| | SCC | 0.7368 | 0.7778 | 0.7568 | | | |
| Fold 7 | AC | 0.5385 | 0.5833 | 0.5600 | 0.6250 | 0.7037 | 0.2472 |
| | SCC | 0.7059 | 0.6667 | 0.6857 | | | |
| Fold 8 | AC | 0.5385 | 0.5833 | 0.5600 | 0.6250 | 0.6759 | 0.2472 |
| | SCC | 0.7059 | 0.6667 | 0.6857 | | | |
| Fold 9 | AC | 0.5000 | 0.5000 | 0.5000 | 0.5833 | 0.7037 | 0.1667 |
| | SCC | 0.6667 | 0.6667 | 0.6667 | | | |
| Fold 10 | AC | 0.4444 | 0.3333 | 0.3810 | 0.5278 | 0.6713 | 0.0594 |
| | SCC | 0.6190 | 0.7222 | 0.6667 | | | |
| **Mean ± std** | **AC** | 0.5058 ±0.0671 | 0.4583 ±0.1058 | 0.4785 ±0.0843 | 0.5819 ±0.0542 | 0.6745 ±0.0322 | 0.1666 ±0.1086 |
| | **SCC** | 0.6637 ±0.0437 | 0.7056 ±0.0457 | 0.6829 ±0.0340 | | | |

*Table S49: Logistic regression on H-optimus-1 embeddings: Patient-level performance metrics AC vs. SCC.*

| Fold | Class | PRE | REC | F1 | BA | AUROC | MCC |
|---|---|---|---|---|---|---|---|
| Fold 1 | AC | 0.6667 | 0.5000 | 0.5714 | 0.6591 | 0.7500 | 0.3380 |
| | SCC | 0.6923 | 0.8182 | 0.7500 | | | |
| Fold 2 | AC | 0.6667 | 0.5000 | 0.5714 | 0.6591 | 0.8068 | 0.3380 |
| | SCC | 0.6923 | 0.8182 | 0.7500 | | | |
| Fold 3 | AC | 0.6667 | 0.5000 | 0.5714 | 0.6591 | 0.7273 | 0.3380 |
| | SCC | 0.6923 | 0.8182 | 0.7500 | | | |
| Fold 4 | AC | 0.5000 | 0.3750 | 0.4286 | 0.5511 | 0.6818 | 0.1086 |
| | SCC | 0.6154 | 0.7273 | 0.6667 | | | |
| Fold 5 | AC | 0.5714 | 0.5000 | 0.5333 | 0.6136 | 0.7500 | 0.2326 |
| | SCC | 0.6667 | 0.7273 | 0.6957 | | | |
| Fold 6 | AC | 0.7143 | 0.6250 | 0.6667 | 0.7216 | 0.7500 | 0.4536 |
| | SCC | 0.7500 | 0.8182 | 0.7826 | | | |
| Fold 7 | AC | 0.7500 | 0.7500 | 0.7500 | 0.7841 | 0.8068 | 0.5682 |
| | SCC | 0.8182 | 0.8182 | 0.8182 | | | |
| Fold 8 | AC | 0.7143 | 0.6250 | 0.6667 | 0.7216 | 0.7386 | 0.4536 |
| | SCC | 0.7500 | 0.8182 | 0.7826 | | | |
| Fold 9 | AC | 0.6667 | 0.5000 | 0.5714 | 0.6591 | 0.7273 | 0.3380 |
| | SCC | 0.6923 | 0.8182 | 0.7500 | | | |
| Fold 10 | AC | 0.6000 | 0.3750 | 0.4615 | 0.5966 | 0.7386 | 0.2166 |
| | SCC | 0.6429 | 0.8182 | 0.7200 | | | |
| **Mean ± std** | **AC** | 0.6517 ±0.0749 | 0.5250 ±0.1149 | 0.5792 ±0.0962 | 0.6625 ±0.0675 | 0.7477 ±0.0370 | 0.3385 ±0.1326 |
| | **SCC** | 0.7012 ±0.0584 | 0.8000 ±0.0383 | 0.7466 ±0.0440 | | | |

*Table S50: Logistic regression on hibou-L embeddings: Patch-level performance metrics AC vs. SCC.*

| Fold | Class | PRE | REC | F1 | BA | AUROC | MCC |
|---|---|---|---|---|---|---|---|
| Fold 1 | AC | 0.5050 | 0.3804 | 0.4339 | 0.5654 | 0.6219 | 0.1396 |
| | SCC | 0.6440 | 0.7504 | 0.6931 | | | |
| Fold 2 | AC | 0.5240 | 0.4131 | 0.4620 | 0.5809 | 0.6427 | 0.1706 |
| | SCC | 0.6558 | 0.7487 | 0.6992 | | | |
| Fold 3 | AC | 0.4373 | 0.3249 | 0.3728 | 0.5225 | 0.5638 | 0.0482 |
| | SCC | 0.6144 | 0.7201 | 0.6630 | | | |
| Fold 4 | AC | 0.4645 | 0.3300 | 0.3859 | 0.5377 | 0.5801 | 0.0818 |
| | SCC | 0.6243 | 0.7454 | 0.6795 | | | |
| Fold 5 | AC | 0.4767 | 0.4383 | 0.4567 | 0.5581 | 0.6127 | 0.1180 |
| | SCC | 0.6432 | 0.6779 | 0.6601 | | | |
| Fold 6 | AC | 0.5115 | 0.3929 | 0.4444 | 0.5708 | 0.5996 | 0.1504 |
| | SCC | 0.6482 | 0.7487 | 0.6948 | | | |
| Fold 7 | AC | 0.5015 | 0.4282 | 0.4620 | 0.5716 | 0.6054 | 0.1479 |
| | SCC | 0.6513 | 0.7150 | 0.6817 | | | |
| Fold 8 | AC | 0.4769 | 0.4156 | 0.4441 | 0.5552 | 0.6000 | 0.1135 |
| | SCC | 0.6398 | 0.6948 | 0.6661 | | | |
| Fold 9 | AC | 0.5054 | 0.4736 | 0.4889 | 0.5816 | 0.6316 | 0.1652 |
| | SCC | 0.6618 | 0.6897 | 0.6755 | | | |
| Fold 10 | AC | 0.4343 | 0.3577 | 0.3923 | 0.5229 | 0.5704 | 0.0476 |
| | SCC | 0.6154 | 0.6880 | 0.6497 | | | |
| **Mean ± std** | **AC** | 0.4837 ±0.0311 | 0.3955 ±0.0479 | 0.4343 ±0.0382 | 0.5567 ±0.0221 | 0.6028 ±0.0258 | 0.1183 ±0.0454 |
| | **SCC** | 0.6398 ±0.0165 | 0.7179 ±0.0290 | 0.6763 ±0.0165 | | | |

*Table S51: Logistic regression on hibou-L embeddings: Slide-level performance metrics AC vs. SCC.*

| Fold | Class | PRE | REC | F1 | BA | AUROC | MCC |
|---|---|---|---|---|---|---|---|
| Fold 1 | AC | 0.5385 | 0.5833 | 0.5600 | 0.6250 | 0.6667 | 0.2472 |
| | SCC | 0.7059 | 0.6667 | 0.6857 | | | |
| Fold 2 | AC | 0.6667 | 0.5000 | 0.5714 | 0.6667 | 0.7361 | 0.3563 |
| | SCC | 0.7143 | 0.8333 | 0.7692 | | | |
| Fold 3 | AC | 0.5000 | 0.4167 | 0.4545 | 0.5694 | 0.6111 | 0.1443 |
| | SCC | 0.6500 | 0.7222 | 0.6842 | | | |
| Fold 4 | AC | 0.5000 | 0.5000 | 0.5000 | 0.5833 | 0.6343 | 0.1667 |
| | SCC | 0.6667 | 0.6667 | 0.6667 | | | |
| Fold 5 | AC | 0.5000 | 0.5000 | 0.5000 | 0.5833 | 0.6574 | 0.1667 |
| | SCC | 0.6667 | 0.6667 | 0.6667 | | | |
| Fold 6 | AC | 0.5000 | 0.5833 | 0.5385 | 0.5972 | 0.5972 | 0.1909 |
| | SCC | 0.6875 | 0.6111 | 0.6471 | | | |
| Fold 7 | AC | 0.5385 | 0.5833 | 0.5600 | 0.6250 | 0.6435 | 0.2472 |
| | SCC | 0.7059 | 0.6667 | 0.6857 | | | |
| Fold 8 | AC | 0.5385 | 0.5833 | 0.5600 | 0.6250 | 0.6898 | 0.2472 |
| | SCC | 0.7059 | 0.6667 | 0.6857 | | | |
| Fold 9 | AC | 0.5455 | 0.5000 | 0.5217 | 0.6111 | 0.7037 | 0.2259 |
| | SCC | 0.6842 | 0.7222 | 0.7027 | | | |
| Fold 10 | AC | 0.4167 | 0.4167 | 0.4167 | 0.5139 | 0.6250 | 0.0278 |
| | SCC | 0.6111 | 0.6111 | 0.6111 | | | |
| **Mean ± std** | **AC** | 0.5244 ±0.0626 | 0.5167 ±0.0657 | 0.5183 ±0.0511 | 0.6000 ±0.0413 | 0.6565 ±0.0435 | 0.2020 ±0.0862 |
| | **SCC** | 0.6798 ±0.0321 | 0.6833 ±0.0644 | 0.6805 ±0.0405 | | | |

*Table S52: Logistic regression on hibou-L embeddings: Patient-level performance metrics AC vs. SCC.*

| Fold | Class | PRE | REC | F1 | BA | AUROC | MCC |
|---|---|---|---|---|---|---|---|
| Fold 1 | AC | 0.6250 | 0.6250 | 0.6250 | 0.6761 | 0.7386 | 0.3523 |
| | SCC | 0.7273 | 0.7273 | 0.7273 | | | |
| Fold 2 | AC | 0.6667 | 0.5000 | 0.5714 | 0.6591 | 0.7955 | 0.3380 |
| | SCC | 0.6923 | 0.8182 | 0.7500 | | | |
| Fold 3 | AC | 0.4286 | 0.3750 | 0.4000 | 0.5057 | 0.6591 | 0.0116 |
| | SCC | 0.5833 | 0.6364 | 0.6087 | | | |
| Fold 4 | AC | 0.5714 | 0.5000 | 0.5333 | 0.6136 | 0.6932 | 0.2326 |
| | SCC | 0.6667 | 0.7273 | 0.6957 | | | |
| Fold 5 | AC | 0.5714 | 0.5000 | 0.5333 | 0.6136 | 0.7159 | 0.2326 |
| | SCC | 0.6667 | 0.7273 | 0.6957 | | | |
| Fold 6 | AC | 0.5556 | 0.6250 | 0.5882 | 0.6307 | 0.6818 | 0.2584 |
| | SCC | 0.7000 | 0.6364 | 0.6667 | | | |
| Fold 7 | AC | 0.5556 | 0.6250 | 0.5882 | 0.6307 | 0.7159 | 0.2584 |
| | SCC | 0.7000 | 0.6364 | 0.6667 | | | |
| Fold 8 | AC | 0.5556 | 0.6250 | 0.5882 | 0.6307 | 0.6932 | 0.2584 |
| | SCC | 0.7000 | 0.6364 | 0.6667 | | | |
| Fold 9 | AC | 0.5000 | 0.5000 | 0.5000 | 0.5682 | 0.7386 | 0.1364 |
| | SCC | 0.6364 | 0.6364 | 0.6364 | | | |
| Fold 10 | AC | 0.4286 | 0.3750 | 0.4000 | 0.5057 | 0.6477 | 0.0116 |
| | SCC | 0.5833 | 0.6364 | 0.6087 | | | |
| **Mean ± std** | **AC** | 0.5458 ±0.0761 | 0.5250 ±0.0986 | 0.5328 ±0.0786 | 0.6034 ±0.0589 | 0.7080 ±0.0432 | 0.2090 ±0.1196 |
| | **SCC** | 0.6656 ±0.0500 | 0.6818 ±0.0643 | 0.6722 ±0.0467 | | | |

*Table S53: Logistic regression on Kaiko embeddings: Patch-level performance metrics AC vs. SCC.*

| Fold | Class | PRE | REC | F1 | BA | AUROC | MCC |
|---|---|---|---|---|---|---|---|
| Fold 1 | AC | 0.4824 | 0.3451 | 0.4023 | 0.5486 | 0.5736 | 0.1053 |
| | SCC | 0.6317 | 0.7521 | 0.6867 | | | |
| Fold 2 | AC | 0.4645 | 0.3627 | 0.4074 | 0.5414 | 0.5663 | 0.0875 |
| | SCC | 0.6279 | 0.7201 | 0.6709 | | | |
| Fold 3 | AC | 0.3986 | 0.2872 | 0.3338 | 0.4986 | 0.5090 | -0.0031 |
| | SCC | 0.5980 | 0.7099 | 0.6492 | | | |
| Fold 4 | AC | 0.4348 | 0.3023 | 0.3566 | 0.5196 | 0.5343 | 0.0428 |
| | SCC | 0.6120 | 0.7369 | 0.6687 | | | |
| Fold 5 | AC | 0.4348 | 0.4282 | 0.4315 | 0.5278 | 0.5404 | 0.0557 |
| | SCC | 0.6210 | 0.6273 | 0.6242 | | | |
| Fold 6 | AC | 0.4752 | 0.3627 | 0.4114 | 0.5473 | 0.5464 | 0.1006 |
| | SCC | 0.6317 | 0.7319 | 0.6781 | | | |
| Fold 7 | AC | 0.4938 | 0.3980 | 0.4407 | 0.5624 | 0.5805 | 0.1308 |
| | SCC | 0.6433 | 0.7268 | 0.6825 | | | |
| Fold 8 | AC | 0.4286 | 0.3854 | 0.4058 | 0.5207 | 0.5460 | 0.0422 |
| | SCC | 0.6145 | 0.6560 | 0.6346 | | | |
| Fold 9 | AC | 0.4798 | 0.4181 | 0.4468 | 0.5573 | 0.5943 | 0.1178 |
| | SCC | 0.6413 | 0.6965 | 0.6677 | | | |
| Fold 10 | AC | 0.4064 | 0.3501 | 0.3762 | 0.5039 | 0.5225 | 0.0080 |
| | SCC | 0.6019 | 0.6577 | 0.6285 | | | |
| **Mean ± std** | **AC** | 0.4499 ±0.0336 | 0.3640 ±0.0459 | 0.4013 ±0.0363 | 0.5327 ±0.0220 | 0.5513 ±0.0269 | 0.0688 ±0.0464 |
| | **SCC** | 0.6223 ±0.0156 | 0.7015 ±0.0413 | 0.6591 ±0.0232 | | | |

*Table S54: Logistic regression on Kaiko embeddings: Slide-level performance metrics AC vs. SCC.*

| Fold | Class | PRE | REC | F1 | BA | AUROC | MCC |
|---|---|---|---|---|---|---|---|
| Fold 1 | AC | 0.4545 | 0.4167 | 0.4348 | 0.5417 | 0.5972 | 0.0847 |
| | SCC | 0.6316 | 0.6667 | 0.6486 | | | |
| Fold 2 | AC | 0.4545 | 0.4167 | 0.4348 | 0.5417 | 0.6343 | 0.0847 |
| | SCC | 0.6316 | 0.6667 | 0.6486 | | | |
| Fold 3 | AC | 0.4000 | 0.3333 | 0.3636 | 0.5000 | 0.5417 | 0.0000 |
| | SCC | 0.6000 | 0.6667 | 0.6316 | | | |
| Fold 4 | AC | 0.4000 | 0.3333 | 0.3636 | 0.5000 | 0.5278 | 0.0000 |
| | SCC | 0.6000 | 0.6667 | 0.6316 | | | |
| Fold 5 | AC | 0.5714 | 0.6667 | 0.6154 | 0.6667 | 0.6343 | 0.3273 |
| | SCC | 0.7500 | 0.6667 | 0.7059 | | | |
| Fold 6 | AC | 0.5000 | 0.4167 | 0.4545 | 0.5694 | 0.5741 | 0.1443 |
| | SCC | 0.6500 | 0.7222 | 0.6842 | | | |
| Fold 7 | AC | 0.5385 | 0.5833 | 0.5600 | 0.6250 | 0.6019 | 0.2472 |
| | SCC | 0.7059 | 0.6667 | 0.6857 | | | |
| Fold 8 | AC | 0.4615 | 0.5000 | 0.4800 | 0.5556 | 0.6019 | 0.1098 |
| | SCC | 0.6471 | 0.6111 | 0.6286 | | | |
| Fold 9 | AC | 0.4444 | 0.3333 | 0.3810 | 0.5278 | 0.6574 | 0.0594 |
| | SCC | 0.6190 | 0.7222 | 0.6667 | | | |
| Fold 10 | AC | 0.4615 | 0.5000 | 0.4800 | 0.5556 | 0.5972 | 0.1098 |
| | SCC | 0.6471 | 0.6111 | 0.6286 | | | |
| **Mean ± std** | **AC** | 0.4687 ±0.0547 | 0.4500 ±0.1125 | 0.4568 ±0.0823 | 0.5583 ±0.0523 | 0.5968 ±0.0406 | 0.1167 ±0.1026 |
| | **SCC** | 0.6482 ±0.0468 | 0.6667 ±0.0370 | 0.6560 ±0.0281 | | | |

*Table S55: Logistic regression on Kaiko embeddings: Patient-level performance metrics AC vs. SCC.*

| Fold | Class | PRE | REC | F1 | BA | AUROC | MCC |
|---|---|---|---|---|---|---|---|
| Fold 1 | AC | 0.4286 | 0.3750 | 0.4000 | 0.5057 | 0.6364 | 0.0116 |
| | SCC | 0.5833 | 0.6364 | 0.6087 | | | |
| Fold 2 | AC | 0.5714 | 0.5000 | 0.5333 | 0.6136 | 0.6705 | 0.2326 |
| | SCC | 0.6667 | 0.7273 | 0.6957 | | | |
| Fold 3 | AC | 0.5000 | 0.3750 | 0.4286 | 0.5511 | 0.6364 | 0.1086 |
| | SCC | 0.6154 | 0.7273 | 0.6667 | | | |
| Fold 4 | AC | 0.5000 | 0.3750 | 0.4286 | 0.5511 | 0.5795 | 0.1086 |
| | SCC | 0.6154 | 0.7273 | 0.6667 | | | |
| Fold 5 | AC | 0.6000 | 0.7500 | 0.6667 | 0.6932 | 0.6818 | 0.3820 |
| | SCC | 0.7778 | 0.6364 | 0.7000 | | | |
| Fold 6 | AC | 0.5000 | 0.3750 | 0.4286 | 0.5511 | 0.6591 | 0.1086 |
| | SCC | 0.6154 | 0.7273 | 0.6667 | | | |
| Fold 7 | AC | 0.6667 | 0.7500 | 0.7059 | 0.7386 | 0.6932 | 0.4719 |
| | SCC | 0.8000 | 0.7273 | 0.7619 | | | |
| Fold 8 | AC | 0.5000 | 0.5000 | 0.5000 | 0.5682 | 0.6250 | 0.1364 |
| | SCC | 0.6364 | 0.6364 | 0.6364 | | | |
| Fold 9 | AC | 0.5000 | 0.3750 | 0.4286 | 0.5511 | 0.7045 | 0.1086 |
| | SCC | 0.6154 | 0.7273 | 0.6667 | | | |
| Fold 10 | AC | 0.5000 | 0.5000 | 0.5000 | 0.5682 | 0.6250 | 0.1364 |
| | SCC | 0.6364 | 0.6364 | 0.6364 | | | |
| **Mean ± std** | **AC** | 0.5267 ±0.0674 | 0.4875 ±0.1497 | 0.5020 ±0.1063 | 0.5892 ±0.0726 | 0.6511 ±0.0379 | 0.1805 ±0.1419 |
| | **SCC** | 0.6562 ±0.0733 | 0.6909 ±0.0469 | 0.6706 ±0.0423 | | | |

*Table S56: Logistic regression on Phikon embeddings: Patch-level performance metrics AC vs. SCC.*

| Fold | Class | PRE | REC | F1 | BA | AUROC | MCC |
|---|---|---|---|---|---|---|---|
| Fold 1 | AC | 0.5109 | 0.3552 | 0.4190 | 0.5638 | 0.6253 | 0.1394 |
| | SCC | 0.6415 | 0.7723 | 0.7008 | | | |
| Fold 2 | AC | 0.5123 | 0.3678 | 0.4282 | 0.5667 | 0.6183 | 0.1444 |
| | SCC | 0.6440 | 0.7656 | 0.6995 | | | |
| Fold 3 | AC | 0.4704 | 0.3401 | 0.3947 | 0.5419 | 0.5649 | 0.0904 |
| | SCC | 0.6273 | 0.7437 | 0.6806 | | | |
| Fold 4 | AC | 0.4905 | 0.3249 | 0.3909 | 0.5495 | 0.5786 | 0.1098 |
| | SCC | 0.6314 | 0.7740 | 0.6955 | | | |
| Fold 5 | AC | 0.4727 | 0.3929 | 0.4292 | 0.5498 | 0.5908 | 0.1035 |
| | SCC | 0.6348 | 0.7066 | 0.6688 | | | |
| Fold 6 | AC | 0.5051 | 0.3753 | 0.4306 | 0.5646 | 0.5769 | 0.1383 |
| | SCC | 0.6432 | 0.7538 | 0.6941 | | | |
| Fold 7 | AC | 0.5032 | 0.3929 | 0.4413 | 0.5666 | 0.6029 | 0.1408 |
| | SCC | 0.6456 | 0.7403 | 0.6897 | | | |
| Fold 8 | AC | 0.4841 | 0.3829 | 0.4276 | 0.5548 | 0.5890 | 0.1155 |
| | SCC | 0.6376 | 0.7268 | 0.6793 | | | |
| Fold 9 | AC | 0.5277 | 0.4559 | 0.4892 | 0.5914 | 0.6310 | 0.1882 |
| | SCC | 0.6662 | 0.7268 | 0.6952 | | | |
| Fold 10 | AC | 0.4220 | 0.2997 | 0.3505 | 0.5124 | 0.5234 | 0.0270 |
| | SCC | 0.6073 | 0.7251 | 0.6610 | | | |
| **Mean ± std** | **AC** | 0.4899 ±0.0300 | 0.3688 ±0.0431 | 0.4201 ±0.0363 | 0.5561 ±0.0205 | 0.5901 ±0.0321 | 0.1197 ±0.0426 |
| | **SCC** | 0.6379 ±0.0150 | 0.7435 ±0.0227 | 0.6864 ±0.0135 | | | |

*Table S57: Logistic regression on Phikon embeddings: Slide-level performance metrics AC vs. SCC.*

| Fold | Class | PRE | REC | F1 | BA | AUROC | MCC |
|---|---|---|---|---|---|---|---|
| Fold 1 | AC | 0.5833 | 0.5833 | 0.5833 | 0.6528 | 0.6111 | 0.3056 |
| | SCC | 0.7222 | 0.7222 | 0.7222 | | | |
| Fold 2 | AC | 0.5385 | 0.5833 | 0.5600 | 0.6250 | 0.6435 | 0.2472 |
| | SCC | 0.7059 | 0.6667 | 0.6857 | | | |
| Fold 3 | AC | 0.4167 | 0.4167 | 0.4167 | 0.5139 | 0.6065 | 0.0278 |
| | SCC | 0.6111 | 0.6111 | 0.6111 | | | |
| Fold 4 | AC | 0.4545 | 0.4167 | 0.4348 | 0.5417 | 0.5417 | 0.0847 |
| | SCC | 0.6316 | 0.6667 | 0.6486 | | | |
| Fold 5 | AC | 0.5714 | 0.6667 | 0.6154 | 0.6667 | 0.6157 | 0.3273 |
| | SCC | 0.7500 | 0.6667 | 0.7059 | | | |
| Fold 6 | AC | 0.5000 | 0.5000 | 0.5000 | 0.5833 | 0.5926 | 0.1667 |
| | SCC | 0.6667 | 0.6667 | 0.6667 | | | |
| Fold 7 | AC | 0.5000 | 0.5000 | 0.5000 | 0.5833 | 0.6065 | 0.1667 |
| | SCC | 0.6667 | 0.6667 | 0.6667 | | | |
| Fold 8 | AC | 0.5385 | 0.5833 | 0.5600 | 0.6250 | 0.5926 | 0.2472 |
| | SCC | 0.7059 | 0.6667 | 0.6857 | | | |
| Fold 9 | AC | 0.5455 | 0.5000 | 0.5217 | 0.6111 | 0.6481 | 0.2259 |
| | SCC | 0.6842 | 0.7222 | 0.7027 | | | |
| Fold 10 | AC | 0.4615 | 0.5000 | 0.4800 | 0.5556 | 0.5185 | 0.1098 |
| | SCC | 0.6471 | 0.6111 | 0.6286 | | | |
| **Mean ± std** | **AC** | 0.5110 ±0.0541 | 0.5250 ±0.0791 | 0.5172 ±0.0637 | 0.5958 ±0.0492 | 0.5977 ±0.0405 | 0.1909 ±0.0971 |
| | **SCC** | 0.6791 ±0.0429 | 0.6667 ±0.0370 | 0.6724 ±0.0353 | | | |

*Table S58: Logistic regression on Phikon embeddings: Patient-level performance metrics AC vs. SCC.*

| Fold | Class | PRE | REC | F1 | BA | AUROC | MCC |
|---|---|---|---|---|---|---|---|
| Fold 1 | AC | 0.6250 | 0.6250 | 0.6250 | 0.6761 | 0.6705 | 0.3523 |
| | SCC | 0.7273 | 0.7273 | 0.7273 | | | |
| Fold 2 | AC | 0.6250 | 0.6250 | 0.6250 | 0.6761 | 0.7045 | 0.3523 |
| | SCC | 0.7273 | 0.7273 | 0.7273 | | | |
| Fold 3 | AC | 0.5714 | 0.5000 | 0.5333 | 0.6136 | 0.6705 | 0.2326 |
| | SCC | 0.6667 | 0.7273 | 0.6957 | | | |
| Fold 4 | AC | 0.5714 | 0.5000 | 0.5333 | 0.6136 | 0.6023 | 0.2326 |
| | SCC | 0.6667 | 0.7273 | 0.6957 | | | |
| Fold 5 | AC | 0.6667 | 0.7500 | 0.7059 | 0.7386 | 0.6932 | 0.4719 |
| | SCC | 0.8000 | 0.7273 | 0.7619 | | | |
| Fold 6 | AC | 0.5000 | 0.5000 | 0.5000 | 0.5682 | 0.6591 | 0.1364 |
| | SCC | 0.6364 | 0.6364 | 0.6364 | | | |
| Fold 7 | AC | 0.5556 | 0.6250 | 0.5882 | 0.6307 | 0.7159 | 0.2584 |
| | SCC | 0.7000 | 0.6364 | 0.6667 | | | |
| Fold 8 | AC | 0.6250 | 0.6250 | 0.6250 | 0.6761 | 0.6705 | 0.3523 |
| | SCC | 0.7273 | 0.7273 | 0.7273 | | | |
| Fold 9 | AC | 0.6250 | 0.6250 | 0.6250 | 0.6761 | 0.6818 | 0.3523 |
| | SCC | 0.7273 | 0.7273 | 0.7273 | | | |
| Fold 10 | AC | 0.5000 | 0.5000 | 0.5000 | 0.5682 | 0.6136 | 0.1364 |
| | SCC | 0.6364 | 0.6364 | 0.6364 | | | |
| **Mean ± std** | **AC** | 0.5865 ±0.0565 | 0.5875 ±0.0844 | 0.5861 ±0.0673 | 0.6438 ±0.0544 | 0.6682 ±0.0363 | 0.2877 ±0.1072 |
| | **SCC** | 0.7015 ±0.0509 | 0.7000 ±0.0439 | 0.7002 ±0.0422 | | | |

*Table S59: Logistic regression on ProvGigaPath embeddings: Patch-level performance metrics AC vs. SCC.*

| Fold | Class | PRE | REC | F1 | BA | AUROC | MCC |
|---|---|---|---|---|---|---|---|
| Fold 1 | AC | 0.4922 | 0.3980 | 0.4401 | 0.5616 | 0.6466 | 0.1289 |
| | SCC | 0.6428 | 0.7251 | 0.6815 | | | |
| Fold 2 | AC | 0.5116 | 0.3879 | 0.4413 | 0.5700 | 0.6174 | 0.1492 |
| | SCC | 0.6473 | 0.7521 | 0.6958 | | | |
| Fold 3 | AC | 0.4491 | 0.3224 | 0.3754 | 0.5288 | 0.5670 | 0.0624 |
| | SCC | 0.6184 | 0.7352 | 0.6718 | | | |
| Fold 4 | AC | 0.4724 | 0.3451 | 0.3988 | 0.5435 | 0.5926 | 0.0938 |
| | SCC | 0.6286 | 0.7420 | 0.6806 | | | |
| Fold 5 | AC | 0.4558 | 0.4030 | 0.4278 | 0.5405 | 0.5796 | 0.0829 |
| | SCC | 0.6291 | 0.6779 | 0.6526 | | | |
| Fold 6 | AC | 0.5000 | 0.4106 | 0.4509 | 0.5679 | 0.5879 | 0.1415 |
| | SCC | 0.6476 | 0.7251 | 0.6842 | | | |
| Fold 7 | AC | 0.5215 | 0.4584 | 0.4879 | 0.5884 | 0.6060 | 0.1814 |
| | SCC | 0.6646 | 0.7184 | 0.6904 | | | |
| Fold 8 | AC | 0.4503 | 0.3879 | 0.4168 | 0.5354 | 0.5757 | 0.0731 |
| | SCC | 0.6250 | 0.6830 | 0.6527 | | | |
| Fold 9 | AC | 0.5180 | 0.5063 | 0.5121 | 0.5955 | 0.6332 | 0.1917 |
| | SCC | 0.6744 | 0.6847 | 0.6795 | | | |
| Fold 10 | AC | 0.4358 | 0.3249 | 0.3723 | 0.5217 | 0.5512 | 0.0464 |
| | SCC | 0.6138 | 0.7184 | 0.6620 | | | |
| **Mean ± std** | **AC** | 0.4807 ±0.0319 | 0.3945 ±0.0572 | 0.4323 ±0.0449 | 0.5553 ±0.0252 | 0.5957 ±0.0300 | 0.1151 ±0.0506 |
| | **SCC** | 0.6392 ±0.0198 | 0.7162 ±0.0259 | 0.6751 ±0.0150 | | | |

*Table S60: Logistic regression on ProvGigaPath embeddings: Slide-level performance metrics AC vs. SCC.*

| Fold | Class | PRE | REC | F1 | BA | AUROC | MCC |
|---|---|---|---|---|---|---|---|
| Fold 1 | AC | 0.6000 | 0.5000 | 0.5455 | 0.6389 | 0.7269 | 0.2887 |
| | SCC | 0.7000 | 0.7778 | 0.7368 | | | |
| Fold 2 | AC | 0.6000 | 0.5000 | 0.5455 | 0.6389 | 0.7037 | 0.2887 |
| | SCC | 0.7000 | 0.7778 | 0.7368 | | | |
| Fold 3 | AC | 0.5000 | 0.3333 | 0.4000 | 0.5556 | 0.6528 | 0.1231 |
| | SCC | 0.6364 | 0.7778 | 0.7000 | | | |
| Fold 4 | AC | 0.4545 | 0.4167 | 0.4348 | 0.5417 | 0.6574 | 0.0847 |
| | SCC | 0.6316 | 0.6667 | 0.6486 | | | |
| Fold 5 | AC | 0.5714 | 0.6667 | 0.6154 | 0.6667 | 0.7037 | 0.3273 |
| | SCC | 0.7500 | 0.6667 | 0.7059 | | | |
| Fold 6 | AC | 0.5833 | 0.5833 | 0.5833 | 0.6528 | 0.6481 | 0.3056 |
| | SCC | 0.7222 | 0.7222 | 0.7222 | | | |
| Fold 7 | AC | 0.5385 | 0.5833 | 0.5600 | 0.6250 | 0.6852 | 0.2472 |
| | SCC | 0.7059 | 0.6667 | 0.6857 | | | |
| Fold 8 | AC | 0.5385 | 0.5833 | 0.5600 | 0.6250 | 0.6713 | 0.2472 |
| | SCC | 0.7059 | 0.6667 | 0.6857 | | | |
| Fold 9 | AC | 0.5714 | 0.6667 | 0.6154 | 0.6667 | 0.7731 | 0.3273 |
| | SCC | 0.7500 | 0.6667 | 0.7059 | | | |
| Fold 10 | AC | 0.4545 | 0.4167 | 0.4348 | 0.5417 | 0.6389 | 0.0847 |
| | SCC | 0.6316 | 0.6667 | 0.6486 | | | |
| **Mean ± std** | **AC** | 0.5412 ±0.0550 | 0.5250 ±0.1115 | 0.5295 ±0.0780 | 0.6153 ±0.0499 | 0.6861 ±0.0417 | 0.2324 ±0.0976 |
| | **SCC** | 0.6934 ±0.0454 | 0.7056 ±0.0527 | 0.6976 ±0.0315 | | | |

*Table S61: Logistic regression on ProvGigaPath embeddings: Patient-level performance metrics AC vs. SCC.*

| Fold | Class | PRE | REC | F1 | BA | AUROC | MCC |
|---|---|---|---|---|---|---|---|
| Fold 1 | AC | 0.5714 | 0.5000 | 0.5333 | 0.6136 | 0.7500 | 0.2326 |
| | SCC | 0.6667 | 0.7273 | 0.6957 | | | |
| Fold 2 | AC | 0.5714 | 0.5000 | 0.5333 | 0.6136 | 0.7273 | 0.2326 |
| | SCC | 0.6667 | 0.7273 | 0.6957 | | | |
| Fold 3 | AC | 0.5000 | 0.3750 | 0.4286 | 0.5511 | 0.6932 | 0.1086 |
| | SCC | 0.6154 | 0.7273 | 0.6667 | | | |
| Fold 4 | AC | 0.5000 | 0.3750 | 0.4286 | 0.5511 | 0.6705 | 0.1086 |
| | SCC | 0.6154 | 0.7273 | 0.6667 | | | |
| Fold 5 | AC | 0.6000 | 0.7500 | 0.6667 | 0.6932 | 0.7273 | 0.3820 |
| | SCC | 0.7778 | 0.6364 | 0.7000 | | | |
| Fold 6 | AC | 0.6250 | 0.6250 | 0.6250 | 0.6761 | 0.6932 | 0.3523 |
| | SCC | 0.7273 | 0.7273 | 0.7273 | | | |
| Fold 7 | AC | 0.6250 | 0.6250 | 0.6250 | 0.6761 | 0.7273 | 0.3523 |
| | SCC | 0.7273 | 0.7273 | 0.7273 | | | |
| Fold 8 | AC | 0.5556 | 0.6250 | 0.5882 | 0.6307 | 0.6932 | 0.2584 |
| | SCC | 0.7000 | 0.6364 | 0.6667 | | | |
| Fold 9 | AC | 0.5556 | 0.6250 | 0.5882 | 0.6307 | 0.7727 | 0.2584 |
| | SCC | 0.7000 | 0.6364 | 0.6667 | | | |
| Fold 10 | AC | 0.5000 | 0.3750 | 0.4286 | 0.5511 | 0.6818 | 0.1086 |
| | SCC | 0.6154 | 0.7273 | 0.6667 | | | |
| **Mean ± std** | **AC** | 0.5604 ±0.0485 | 0.5375 ±0.1324 | 0.5446 ±0.0896 | 0.6188 ±0.0539 | 0.7136 ±0.0325 | 0.2395 ±0.1043 |
| | **SCC** | 0.6812 ±0.0555 | 0.7000 ±0.0439 | 0.6879 ±0.0250 | | | |

*Table S62: Logistic regression on ResNet50 embeddings: Patch-level performance metrics AC vs. SCC.*

| Fold | Class | PRE | REC | F1 | BA | AUROC | MCC |
|---|---|---|---|---|---|---|---|
| Fold 1 | AC | 0.4375 | 0.4055 | 0.4209 | 0.5282 | 0.5513 | 0.0573 |
| | SCC | 0.6206 | 0.6509 | 0.6354 | | | |
| Fold 2 | AC | 0.4529 | 0.4484 | 0.4506 | 0.5429 | 0.5753 | 0.0859 |
| | SCC | 0.6332 | 0.6374 | 0.6353 | | | |
| Fold 3 | AC | 0.4015 | 0.4055 | 0.4035 | 0.5004 | 0.5263 | 0.0008 |
| | SCC | 0.5993 | 0.5953 | 0.5973 | | | |
| Fold 4 | AC | 0.4311 | 0.4332 | 0.4322 | 0.5252 | 0.5524 | 0.0504 |
| | SCC | 0.6193 | 0.6172 | 0.6182 | | | |
| Fold 5 | AC | 0.4282 | 0.4660 | 0.4463 | 0.5247 | 0.5509 | 0.0489 |
| | SCC | 0.6201 | 0.5835 | 0.6012 | | | |
| Fold 6 | AC | 0.4203 | 0.4181 | 0.4192 | 0.5160 | 0.5228 | 0.0320 |
| | SCC | 0.6118 | 0.6138 | 0.6128 | | | |
| Fold 7 | AC | 0.4400 | 0.4433 | 0.4417 | 0.5328 | 0.5459 | 0.0655 |
| | SCC | 0.6254 | 0.6223 | 0.6238 | | | |
| Fold 8 | AC | 0.4389 | 0.4887 | 0.4625 | 0.5352 | 0.5563 | 0.0695 |
| | SCC | 0.6296 | 0.5818 | 0.6047 | | | |
| Fold 9 | AC | 0.4463 | 0.4710 | 0.4583 | 0.5399 | 0.5649 | 0.0792 |
| | SCC | 0.6322 | 0.6088 | 0.6203 | | | |
| Fold 10 | AC | 0.4016 | 0.3904 | 0.3959 | 0.5004 | 0.5207 | 0.0009 |
| | SCC | 0.5993 | 0.6105 | 0.6048 | | | |
| **Mean ± std** | **AC** | 0.4298 ±0.0175 | 0.4370 ±0.0322 | 0.4331 ±0.0227 | 0.5246 ±0.0149 | 0.5467 ±0.0182 | 0.0490 ±0.0297 |
| | **SCC** | 0.6191 ±0.0123 | 0.6121 ±0.0219 | 0.6154 ±0.0136 | | | |

*Table S63: Logistic regression on ResNet50 embeddings: Slide-level performance metrics AC vs. SCC.*

| Fold | Class | PRE | REC | F1 | BA | AUROC | MCC |
|---|---|---|---|---|---|---|---|
| Fold 1 | AC | 0.3750 | 0.2500 | 0.3000 | 0.4861 | 0.4954 | -0.0308 |
| | SCC | 0.5909 | 0.7222 | 0.6500 | | | |
| Fold 2 | AC | 0.3636 | 0.3333 | 0.3478 | 0.4722 | 0.5139 | -0.0565 |
| | SCC | 0.5789 | 0.6111 | 0.5946 | | | |
| Fold 3 | AC | 0.2727 | 0.2500 | 0.2609 | 0.4028 | 0.4259 | -0.1977 |
| | SCC | 0.5263 | 0.5556 | 0.5405 | | | |
| Fold 4 | AC | 0.4167 | 0.4167 | 0.4167 | 0.5139 | 0.4861 | 0.0278 |
| | SCC | 0.6111 | 0.6111 | 0.6111 | | | |
| Fold 5 | AC | 0.4167 | 0.4167 | 0.4167 | 0.5139 | 0.5509 | 0.0278 |
| | SCC | 0.6111 | 0.6111 | 0.6111 | | | |
| Fold 6 | AC | 0.5000 | 0.5000 | 0.5000 | 0.5833 | 0.5185 | 0.1667 |
| | SCC | 0.6667 | 0.6667 | 0.6667 | | | |
| Fold 7 | AC | 0.4545 | 0.4167 | 0.4348 | 0.5417 | 0.5093 | 0.0847 |
| | SCC | 0.6316 | 0.6667 | 0.6486 | | | |
| Fold 8 | AC | 0.4545 | 0.4167 | 0.4348 | 0.5417 | 0.5926 | 0.0847 |
| | SCC | 0.6316 | 0.6667 | 0.6486 | | | |
| Fold 9 | AC | 0.4167 | 0.4167 | 0.4167 | 0.5139 | 0.5787 | 0.0278 |
| | SCC | 0.6111 | 0.6111 | 0.6111 | | | |
| Fold 10 | AC | 0.4000 | 0.3333 | 0.3636 | 0.5000 | 0.4861 | 0.0000 |
| | SCC | 0.6000 | 0.6667 | 0.6316 | | | |
| **Mean ± std** | **AC** | 0.4070 ±0.0619 | 0.3750 ±0.0810 | 0.3892 ±0.0711 | 0.5069 ±0.0482 | 0.5157 ±0.0488 | 0.0135 ±0.0977 |
| | **SCC** | 0.6059 ±0.0371 | 0.6389 ±0.0472 | 0.6214 ±0.0366 | | | |

*Table S64: Logistic regression on ResNet50 embeddings: Patient-level performance metrics AC vs. SCC.*

| Fold | Class | PRE | REC | F1 | BA | AUROC | MCC |
|---|---|---|---|---|---|---|---|
| Fold 1 | AC | 0.3333 | 0.2500 | 0.2857 | 0.4432 | 0.5455 | -0.1207 |
| | SCC | 0.5385 | 0.6364 | 0.5833 | | | |
| Fold 2 | AC | 0.5000 | 0.2500 | 0.3333 | 0.5341 | 0.5455 | 0.0826 |
| | SCC | 0.6000 | 0.8182 | 0.6923 | | | |
| Fold 3 | AC | 0.1667 | 0.1250 | 0.1429 | 0.3352 | 0.4091 | -0.3500 |
| | SCC | 0.4615 | 0.5455 | 0.5000 | | | |
| Fold 4 | AC | 0.5000 | 0.5000 | 0.5000 | 0.5682 | 0.5227 | 0.1364 |
| | SCC | 0.6364 | 0.6364 | 0.6364 | | | |
| Fold 5 | AC | 0.5714 | 0.5000 | 0.5333 | 0.6136 | 0.5682 | 0.2326 |
| | SCC | 0.6667 | 0.7273 | 0.6957 | | | |
| Fold 6 | AC | 0.5000 | 0.5000 | 0.5000 | 0.5682 | 0.5795 | 0.1364 |
| | SCC | 0.6364 | 0.6364 | 0.6364 | | | |
| Fold 7 | AC | 0.4286 | 0.3750 | 0.4000 | 0.5057 | 0.5909 | 0.0116 |
| | SCC | 0.5833 | 0.6364 | 0.6087 | | | |
| Fold 8 | AC | 0.4444 | 0.5000 | 0.4706 | 0.5227 | 0.5682 | 0.0449 |
| | SCC | 0.6000 | 0.5455 | 0.5714 | | | |
| Fold 9 | AC | 0.5000 | 0.3750 | 0.4286 | 0.5511 | 0.6136 | 0.1086 |
| | SCC | 0.6154 | 0.7273 | 0.6667 | | | |
| Fold 10 | AC | 0.5000 | 0.3750 | 0.4286 | 0.5511 | 0.5114 | 0.1086 |
| | SCC | 0.6154 | 0.7273 | 0.6667 | | | |
| **Mean ± std** | **AC** | 0.4444 ±0.1161 | 0.3750 ±0.1318 | 0.4023 ±0.1192 | 0.5193 ±0.0787 | 0.5455 ±0.0569 | 0.0391 ±0.1652 |
| | **SCC** | 0.5953 ±0.0583 | 0.6636 ±0.0862 | 0.6257 ±0.0612 | | | |

*Table S65: Logistic regression on TissueConcepts embeddings: Patch-level performance metrics AC vs. SCC.*

| Fold | Class | PRE | REC | F1 | BA | AUROC | MCC |
|---|---|---|---|---|---|---|---|
| Fold 1 | AC | 0.4510 | 0.3829 | 0.4142 | 0.5354 | 0.5627 | 0.0733 |
| | SCC | 0.6248 | 0.6880 | 0.6549 | | | |
| Fold 2 | AC | 0.4511 | 0.4181 | 0.4340 | 0.5387 | 0.5815 | 0.0786 |
| | SCC | 0.6286 | 0.6594 | 0.6436 | | | |
| Fold 3 | AC | 0.4426 | 0.3980 | 0.4191 | 0.5312 | 0.5510 | 0.0637 |
| | SCC | 0.6224 | 0.6644 | 0.6427 | | | |
| Fold 4 | AC | 0.4398 | 0.3678 | 0.4005 | 0.5270 | 0.5317 | 0.0562 |
| | SCC | 0.6185 | 0.6863 | 0.6507 | | | |
| Fold 5 | AC | 0.4427 | 0.4383 | 0.4405 | 0.5345 | 0.5387 | 0.0691 |
| | SCC | 0.6265 | 0.6307 | 0.6286 | | | |
| Fold 6 | AC | 0.4231 | 0.3879 | 0.4047 | 0.5169 | 0.5148 | 0.0343 |
| | SCC | 0.6118 | 0.6459 | 0.6284 | | | |
| Fold 7 | AC | 0.4571 | 0.4433 | 0.4501 | 0.5454 | 0.5478 | 0.0914 |
| | SCC | 0.6347 | 0.6476 | 0.6411 | | | |
| Fold 8 | AC | 0.4486 | 0.4509 | 0.4497 | 0.5399 | 0.5643 | 0.0798 |
| | SCC | 0.6311 | 0.6290 | 0.6301 | | | |
| Fold 9 | AC | 0.4552 | 0.4484 | 0.4518 | 0.5446 | 0.5708 | 0.0894 |
| | SCC | 0.6344 | 0.6408 | 0.6376 | | | |
| Fold 10 | AC | 0.4251 | 0.4005 | 0.4125 | 0.5190 | 0.5304 | 0.0384 |
| | SCC | 0.6136 | 0.6374 | 0.6253 | | | |
| **Mean ± std** | **AC** | 0.4436 ±0.0117 | 0.4136 ±0.0302 | 0.4277 ±0.0198 | 0.5333 ±0.0098 | 0.5494 ±0.0208 | 0.0674 ±0.0196 |
| | **SCC** | 0.6247 ±0.0081 | 0.6530 ±0.0212 | 0.6383 ±0.0101 | | | |

*Table S66: Logistic regression on TissueConcepts embeddings: Slide-level performance metrics AC vs. SCC.*

| Fold | Class | PRE | REC | F1 | BA | AUROC | MCC |
|---|---|---|---|---|---|---|---|
| Fold 1 | AC | 0.5000 | 0.5833 | 0.5385 | 0.5972 | 0.5463 | 0.1909 |
| | SCC | 0.6875 | 0.6111 | 0.6471 | | | |
| Fold 2 | AC | 0.4667 | 0.5833 | 0.5185 | 0.5694 | 0.5370 | 0.1361 |
| | SCC | 0.6667 | 0.5556 | 0.6061 | | | |
| Fold 3 | AC | 0.4375 | 0.5833 | 0.5000 | 0.5417 | 0.5000 | 0.0818 |
| | SCC | 0.6429 | 0.5000 | 0.5625 | | | |
| Fold 4 | AC | 0.4375 | 0.5833 | 0.5000 | 0.5417 | 0.4583 | 0.0818 |
| | SCC | 0.6429 | 0.5000 | 0.5625 | | | |
| Fold 5 | AC | 0.4375 | 0.5833 | 0.5000 | 0.5417 | 0.5324 | 0.0818 |
| | SCC | 0.6429 | 0.5000 | 0.5625 | | | |
| Fold 6 | AC | 0.5000 | 0.5833 | 0.5385 | 0.5972 | 0.5000 | 0.1909 |
| | SCC | 0.6875 | 0.6111 | 0.6471 | | | |
| Fold 7 | AC | 0.5000 | 0.5833 | 0.5385 | 0.5972 | 0.5556 | 0.1909 |
| | SCC | 0.6875 | 0.6111 | 0.6471 | | | |
| Fold 8 | AC | 0.4667 | 0.5833 | 0.5185 | 0.5694 | 0.5417 | 0.1361 |
| | SCC | 0.6667 | 0.5556 | 0.6061 | | | |
| Fold 9 | AC | 0.4667 | 0.5833 | 0.5185 | 0.5694 | 0.5509 | 0.1361 |
| | SCC | 0.6667 | 0.5556 | 0.6061 | | | |
| Fold 10 | AC | 0.3571 | 0.4167 | 0.3846 | 0.4583 | 0.4954 | -0.0818 |
| | SCC | 0.5625 | 0.5000 | 0.5294 | | | |
| **Mean ± std** | **AC** | 0.4570 ±0.0434 | 0.5667 ±0.0527 | 0.5056 ±0.0453 | 0.5583 ±0.0418 | 0.5218 ±0.0316 | 0.1145 ±0.0821 |
| | **SCC** | 0.6554 ±0.0374 | 0.5500 ±0.0486 | 0.5976 ±0.0420 | | | |

*Table S67: Logistic regression on TissueConcepts embeddings: Patient-level performance metrics AC vs. SCC.*

| Fold | Class | PRE | REC | F1 | BA | AUROC | MCC |
|---|---|---|---|---|---|---|---|
| Fold 1 | AC | 0.5556 | 0.6250 | 0.5882 | 0.6307 | 0.6250 | 0.2584 |
| | SCC | 0.7000 | 0.6364 | 0.6667 | | | |
| Fold 2 | AC | 0.5000 | 0.6250 | 0.5556 | 0.5852 | 0.6023 | 0.1685 |
| | SCC | 0.6667 | 0.5455 | 0.6000 | | | |
| Fold 3 | AC | 0.5000 | 0.6250 | 0.5556 | 0.5852 | 0.5568 | 0.1685 |
| | SCC | 0.6667 | 0.5455 | 0.6000 | | | |
| Fold 4 | AC | 0.5556 | 0.6250 | 0.5882 | 0.6307 | 0.5000 | 0.2584 |
| | SCC | 0.7000 | 0.6364 | 0.6667 | | | |
| Fold 5 | AC | 0.4545 | 0.6250 | 0.5263 | 0.5398 | 0.6023 | 0.0795 |
| | SCC | 0.6250 | 0.4545 | 0.5263 | | | |
| Fold 6 | AC | 0.5000 | 0.6250 | 0.5556 | 0.5852 | 0.5682 | 0.1685 |
| | SCC | 0.6667 | 0.5455 | 0.6000 | | | |
| Fold 7 | AC | 0.5556 | 0.6250 | 0.5882 | 0.6307 | 0.5909 | 0.2584 |
| | SCC | 0.7000 | 0.6364 | 0.6667 | | | |
| Fold 8 | AC | 0.5000 | 0.6250 | 0.5556 | 0.5852 | 0.6023 | 0.1685 |
| | SCC | 0.6667 | 0.5455 | 0.6000 | | | |
| Fold 9 | AC | 0.5556 | 0.6250 | 0.5882 | 0.6307 | 0.5795 | 0.2584 |
| | SCC | 0.7000 | 0.6364 | 0.6667 | | | |
| Fold 10 | AC | 0.4000 | 0.5000 | 0.4444 | 0.4773 | 0.5682 | -0.0449 |
| | SCC | 0.5556 | 0.4545 | 0.5000 | | | |
| **Mean ± std** | **AC** | 0.5077 ±0.0514 | 0.6125 ±0.0395 | 0.5546 ±0.0440 | 0.5881 ±0.0493 | 0.5795 ±0.0347 | 0.1743 ±0.0975 |
| | **SCC** | 0.6647 ±0.0453 | 0.5636 ±0.0717 | 0.6093 ±0.0599 | | | |

*Table S68: Logistic regression on UNI2-h embeddings: Patch-level performance metrics AC vs. SCC.*

| Fold | Class | PRE | REC | F1 | BA | AUROC | MCC |
|---|---|---|---|---|---|---|---|
| Fold 1 | AC | 0.5243 | 0.4081 | 0.4589 | 0.5801 | 0.6384 | 0.1694 |
| | SCC | 0.6549 | 0.7521 | 0.7002 | | | |
| Fold 2 | AC | 0.5292 | 0.4332 | 0.4765 | 0.5876 | 0.6411 | 0.1829 |
| | SCC | 0.6617 | 0.7420 | 0.6995 | | | |
| Fold 3 | AC | 0.5000 | 0.3904 | 0.4385 | 0.5645 | 0.6098 | 0.1364 |
| | SCC | 0.6441 | 0.7386 | 0.6881 | | | |
| Fold 4 | AC | 0.4817 | 0.3652 | 0.4155 | 0.5511 | 0.5817 | 0.1089 |
| | SCC | 0.6343 | 0.7369 | 0.6817 | | | |
| Fold 5 | AC | 0.5116 | 0.5013 | 0.5064 | 0.5904 | 0.6203 | 0.1815 |
| | SCC | 0.6705 | 0.6796 | 0.6750 | | | |
| Fold 6 | AC | 0.5284 | 0.4685 | 0.4967 | 0.5943 | 0.6050 | 0.1931 |
| | SCC | 0.6693 | 0.7201 | 0.6937 | | | |
| Fold 7 | AC | 0.5370 | 0.4937 | 0.5144 | 0.6044 | 0.6526 | 0.2120 |
| | SCC | 0.6784 | 0.7150 | 0.6962 | | | |
| Fold 8 | AC | 0.4890 | 0.4484 | 0.4678 | 0.5674 | 0.6092 | 0.1369 |
| | SCC | 0.6502 | 0.6863 | 0.6678 | | | |
| Fold 9 | AC | 0.5379 | 0.5189 | 0.5282 | 0.6102 | 0.6606 | 0.2218 |
| | SCC | 0.6853 | 0.7015 | 0.6933 | | | |
| Fold 10 | AC | 0.4735 | 0.4055 | 0.4369 | 0.5518 | 0.6004 | 0.1070 |
| | SCC | 0.6369 | 0.6981 | 0.6661 | | | |
| **Mean ± std** | **AC** | 0.5113 ±0.0237 | 0.4433 ±0.0516 | 0.4740 ±0.0372 | 0.5802 ±0.0208 | 0.6219 ±0.0253 | 0.1650 ±0.0408 |
| | **SCC** | 0.6586 ±0.0174 | 0.7170 ±0.0251 | 0.6862 ±0.0128 | | | |

*Table S69: Logistic regression on UNI2-h embeddings: Slide-level performance metrics AC vs. SCC.*

| Fold | Class | PRE | REC | F1 | BA | AUROC | MCC |
|---|---|---|---|---|---|---|---|
| Fold 1 | AC | 0.4167 | 0.4167 | 0.4167 | 0.5139 | 0.6435 | 0.0278 |
| | SCC | 0.6111 | 0.6111 | 0.6111 | | | |
| Fold 2 | AC | 0.5556 | 0.4167 | 0.4762 | 0.5972 | 0.7269 | 0.2079 |
| | SCC | 0.6667 | 0.7778 | 0.7179 | | | |
| Fold 3 | AC | 0.5556 | 0.4167 | 0.4762 | 0.5972 | 0.6991 | 0.2079 |
| | SCC | 0.6667 | 0.7778 | 0.7179 | | | |
| Fold 4 | AC | 0.5000 | 0.4167 | 0.4545 | 0.5694 | 0.6157 | 0.1443 |
| | SCC | 0.6500 | 0.7222 | 0.6842 | | | |
| Fold 5 | AC | 0.6250 | 0.8333 | 0.7143 | 0.7500 | 0.6806 | 0.4910 |
| | SCC | 0.8571 | 0.6667 | 0.7500 | | | |
| Fold 6 | AC | 0.5833 | 0.5833 | 0.5833 | 0.6528 | 0.6574 | 0.3056 |
| | SCC | 0.7222 | 0.7222 | 0.7222 | | | |
| Fold 7 | AC | 0.6154 | 0.6667 | 0.6400 | 0.6944 | 0.7176 | 0.3845 |
| | SCC | 0.7647 | 0.7222 | 0.7429 | | | |
| Fold 8 | AC | 0.5000 | 0.5833 | 0.5385 | 0.5972 | 0.6343 | 0.1909 |
| | SCC | 0.6875 | 0.6111 | 0.6471 | | | |
| Fold 9 | AC | 0.6154 | 0.6667 | 0.6400 | 0.6944 | 0.7083 | 0.3845 |
| | SCC | 0.7647 | 0.7222 | 0.7429 | | | |
| Fold 10 | AC | 0.4000 | 0.3333 | 0.3636 | 0.5000 | 0.6435 | 0.0000 |
| | SCC | 0.6000 | 0.6667 | 0.6316 | | | |
| **Mean ± std** | **AC** | 0.5367 ±0.0809 | 0.5333 ±0.1581 | 0.5303 ±0.1121 | 0.6167 ±0.0808 | 0.6727 ±0.0389 | 0.2344 ±0.1581 |
| | **SCC** | 0.6991 ±0.0791 | 0.7000 ±0.0597 | 0.6968 ±0.0504 | | | |

*Table S70: Logistic regression on UNI2-h embeddings: Patient-level performance metrics AC vs. SCC.*

| Fold | Class | PRE | REC | F1 | BA | AUROC | MCC |
|---|---|---|---|---|---|---|---|
| Fold 1 | AC | 0.5000 | 0.3750 | 0.4286 | 0.5511 | 0.7386 | 0.1086 |
| | SCC | 0.6154 | 0.7273 | 0.6667 | | | |
| Fold 2 | AC | 0.6667 | 0.5000 | 0.5714 | 0.6591 | 0.7841 | 0.3380 |
| | SCC | 0.6923 | 0.8182 | 0.7500 | | | |
| Fold 3 | AC | 0.6667 | 0.5000 | 0.5714 | 0.6591 | 0.7273 | 0.3380 |
| | SCC | 0.6923 | 0.8182 | 0.7500 | | | |
| Fold 4 | AC | 0.5714 | 0.5000 | 0.5333 | 0.6136 | 0.6591 | 0.2326 |
| | SCC | 0.6667 | 0.7273 | 0.6957 | | | |
| Fold 5 | AC | 0.7000 | 0.8750 | 0.7778 | 0.8011 | 0.7727 | 0.5955 |
| | SCC | 0.8889 | 0.7273 | 0.8000 | | | |
| Fold 6 | AC | 0.6250 | 0.6250 | 0.6250 | 0.6761 | 0.7159 | 0.3523 |
| | SCC | 0.7273 | 0.7273 | 0.7273 | | | |
| Fold 7 | AC | 0.7000 | 0.8750 | 0.7778 | 0.8011 | 0.7841 | 0.5955 |
| | SCC | 0.8889 | 0.7273 | 0.8000 | | | |
| Fold 8 | AC | 0.5556 | 0.6250 | 0.5882 | 0.6307 | 0.7045 | 0.2584 |
| | SCC | 0.7000 | 0.6364 | 0.6667 | | | |
| Fold 9 | AC | 0.6667 | 0.7500 | 0.7059 | 0.7386 | 0.7841 | 0.4719 |
| | SCC | 0.8000 | 0.7273 | 0.7619 | | | |
| Fold 10 | AC | 0.5000 | 0.3750 | 0.4286 | 0.5511 | 0.7159 | 0.1086 |
| | SCC | 0.6154 | 0.7273 | 0.6667 | | | |
| **Mean ± std** | **AC** | 0.6152 ±0.0778 | 0.6000 ±0.1845 | 0.6008 ±0.1247 | 0.6682 ±0.0896 | 0.7386 ±0.0422 | 0.3400 ±0.1743 |
| | **SCC** | 0.7287 ±0.0997 | 0.7364 ±0.0516 | 0.7285 ±0.0525 | | | |

*Table S71: Logistic regression on Virchow2 embeddings: Patch-level performance metrics AC vs. SCC.*

| Fold | Class | PRE | REC | F1 | BA | AUROC | MCC |
|---|---|---|---|---|---|---|---|
| Fold 1 | AC | 0.4551 | 0.3577 | 0.4006 | 0.5355 | 0.5844 | 0.0749 |
| | SCC | 0.6239 | 0.7133 | 0.6656 | | | |
| Fold 2 | AC | 0.5015 | 0.4131 | 0.4530 | 0.5691 | 0.6091 | 0.1440 |
| | SCC | 0.6486 | 0.7251 | 0.6847 | | | |
| Fold 3 | AC | 0.4281 | 0.3375 | 0.3775 | 0.5178 | 0.5428 | 0.0376 |
| | SCC | 0.6115 | 0.6981 | 0.6520 | | | |
| Fold 4 | AC | 0.4693 | 0.3275 | 0.3858 | 0.5398 | 0.5597 | 0.0869 |
| | SCC | 0.6255 | 0.7521 | 0.6830 | | | |
| Fold 5 | AC | 0.4338 | 0.4207 | 0.4271 | 0.5265 | 0.5501 | 0.0533 |
| | SCC | 0.6198 | 0.6324 | 0.6260 | | | |
| Fold 6 | AC | 0.4799 | 0.3904 | 0.4306 | 0.5536 | 0.5589 | 0.1120 |
| | SCC | 0.6372 | 0.7167 | 0.6746 | | | |
| Fold 7 | AC | 0.4797 | 0.4458 | 0.4621 | 0.5610 | 0.5913 | 0.1237 |
| | SCC | 0.6457 | 0.6762 | 0.6606 | | | |
| Fold 8 | AC | 0.4684 | 0.4106 | 0.4376 | 0.5493 | 0.5812 | 0.1012 |
| | SCC | 0.6355 | 0.6880 | 0.6607 | | | |
| Fold 9 | AC | 0.4621 | 0.4761 | 0.4690 | 0.5525 | 0.5939 | 0.1046 |
| | SCC | 0.6420 | 0.6290 | 0.6354 | | | |
| Fold 10 | AC | 0.4397 | 0.3854 | 0.4107 | 0.5283 | 0.5704 | 0.0581 |
| | SCC | 0.6199 | 0.6712 | 0.6445 | | | |
| **Mean ± std** | **AC** | 0.4618 ±0.0230 | 0.3965 ±0.0468 | 0.4254 ±0.0314 | 0.5433 ±0.0165 | 0.5742 ±0.0213 | 0.0896 ±0.0337 |
| | **SCC** | 0.6310 ±0.0125 | 0.6902 ±0.0394 | 0.6587 ±0.0195 | | | |

*Table S72: Logistic regression on Virchow2 embeddings: Slide-level performance metrics AC vs. SCC.*

| Fold | Class | PRE | REC | F1 | BA | AUROC | MCC |
|---|---|---|---|---|---|---|---|
| Fold 1 | AC | 0.5000 | 0.4167 | 0.4545 | 0.5694 | 0.6250 | 0.1443 |
| | SCC | 0.6500 | 0.7222 | 0.6842 | | | |
| Fold 2 | AC | 0.5000 | 0.4167 | 0.4545 | 0.5694 | 0.6481 | 0.1443 |
| | SCC | 0.6500 | 0.7222 | 0.6842 | | | |
| Fold 3 | AC | 0.4444 | 0.3333 | 0.3810 | 0.5278 | 0.6157 | 0.0594 |
| | SCC | 0.6190 | 0.7222 | 0.6667 | | | |
| Fold 4 | AC | 0.4545 | 0.4167 | 0.4348 | 0.5417 | 0.5648 | 0.0847 |
| | SCC | 0.6316 | 0.6667 | 0.6486 | | | |
| Fold 5 | AC | 0.4615 | 0.5000 | 0.4800 | 0.5556 | 0.5926 | 0.1098 |
| | SCC | 0.6471 | 0.6111 | 0.6286 | | | |
| Fold 6 | AC | 0.5455 | 0.5000 | 0.5217 | 0.6111 | 0.5556 | 0.2259 |
| | SCC | 0.6842 | 0.7222 | 0.7027 | | | |
| Fold 7 | AC | 0.5455 | 0.5000 | 0.5217 | 0.6111 | 0.6204 | 0.2259 |
| | SCC | 0.6842 | 0.7222 | 0.7027 | | | |
| Fold 8 | AC | 0.5455 | 0.5000 | 0.5217 | 0.6111 | 0.6528 | 0.2259 |
| | SCC | 0.6842 | 0.7222 | 0.7027 | | | |
| Fold 9 | AC | 0.5385 | 0.5833 | 0.5600 | 0.6250 | 0.6759 | 0.2472 |
| | SCC | 0.7059 | 0.6667 | 0.6857 | | | |
| Fold 10 | AC | 0.4000 | 0.3333 | 0.3636 | 0.5000 | 0.6157 | 0.0000 |
| | SCC | 0.6000 | 0.6667 | 0.6316 | | | |
| **Mean ± std** | **AC** | 0.4935 ±0.0515 | 0.4500 ±0.0805 | 0.4694 ±0.0643 | 0.5722 ±0.0418 | 0.6167 ±0.0379 | 0.1468 ±0.0838 |
| | **SCC** | 0.6556 ±0.0335 | 0.6944 ±0.0393 | 0.6738 ±0.0286 | | | |

*Table S73: Logistic regression on Virchow2 embeddings: Patient-level performance metrics AC vs. SCC.*

| Fold | Class | PRE | REC | F1 | BA | AUROC | MCC |
|---|---|---|---|---|---|---|---|
| Fold 1 | AC | 0.5000 | 0.3750 | 0.4286 | 0.5511 | 0.6591 | 0.1086 |
| | SCC | 0.6154 | 0.7273 | 0.6667 | | | |
| Fold 2 | AC | 0.6667 | 0.5000 | 0.5714 | 0.6591 | 0.6705 | 0.3380 |
| | SCC | 0.6923 | 0.8182 | 0.7500 | | | |
| Fold 3 | AC | 0.6000 | 0.3750 | 0.4615 | 0.5966 | 0.6364 | 0.2166 |
| | SCC | 0.6429 | 0.8182 | 0.7200 | | | |
| Fold 4 | AC | 0.5714 | 0.5000 | 0.5333 | 0.6136 | 0.5909 | 0.2326 |
| | SCC | 0.6667 | 0.7273 | 0.6957 | | | |
| Fold 5 | AC | 0.6250 | 0.6250 | 0.6250 | 0.6761 | 0.6477 | 0.3523 |
| | SCC | 0.7273 | 0.7273 | 0.7273 | | | |
| Fold 6 | AC | 0.5714 | 0.5000 | 0.5333 | 0.6136 | 0.6023 | 0.2326 |
| | SCC | 0.6667 | 0.7273 | 0.6957 | | | |
| Fold 7 | AC | 0.7143 | 0.6250 | 0.6667 | 0.7216 | 0.6818 | 0.4536 |
| | SCC | 0.7500 | 0.8182 | 0.7826 | | | |
| Fold 8 | AC | 0.6250 | 0.6250 | 0.6250 | 0.6761 | 0.6818 | 0.3523 |
| | SCC | 0.7273 | 0.7273 | 0.7273 | | | |
| Fold 9 | AC | 0.5000 | 0.5000 | 0.5000 | 0.5682 | 0.6705 | 0.1364 |
| | SCC | 0.6364 | 0.6364 | 0.6364 | | | |
| Fold 10 | AC | 0.5000 | 0.3750 | 0.4286 | 0.5511 | 0.6477 | 0.1086 |
| | SCC | 0.6154 | 0.7273 | 0.6667 | | | |
| **Mean ± std** | **AC** | 0.5874 ±0.0736 | 0.5000 ±0.1021 | 0.5373 ±0.0844 | 0.6227 ±0.0586 | 0.6489 ±0.0314 | 0.2532 ±0.1177 |
| | **SCC** | 0.6740 ±0.0485 | 0.7455 ±0.0575 | 0.7068 ±0.0435 | | | |

*Table S74: MLP: Performance metrics AC vs. SCC.*

| Architecture | Level | Class | PRE | REC | F1 | BA (95% CI) | AUROC (95% CI) | MCC (95% CI) |
|---|---|---|---|---|---|---|---|---|
| **ResNet50** | Patch | AC | 0.4561 | 0.4710 | 0.4634 | 0.5475 | 0.5749 | 0.0945 |
| | | SCC | 0.6379 | 0.6239 | 0.6309 | (0.5169-0.5776) | (0.5401-0.6113) | (0.0337-0.1539) |
| | Slide | AC | 0.5455 | 0.5000 | 0.5217 | 0.6111 | 0.5880 | 0.2259 |
| | | SCC | 0.6842 | 0.7222 | 0.7027 | (0.4389-0.7920) | (0.3600-0.8000) | (-0.1321-0.5769) |
| | Patient | AC | 0.5000 | 0.5000 | 0.5000 | 0.5682 | 0.6023 | 0.1364 |
| | | SCC | 0.6364 | 0.6364 | 0.6364 | (0.3222-0.8000) | (0.3214-0.8571) | (-0.3307-0.5866) |
| **CONCH** | Patch | AC | 0.4218 | 0.4005 | 0.4109 | 0.5164 | 0.5482 | 0.0332 |
| | | SCC | 0.6117 | 0.6324 | 0.6219 | (0.4875-0.5476) | (0.5136-0.5826) | (-0.0255-0.0969) |
| | Slide | AC | 0.3846 | 0.4167 | 0.4000 | 0.4861 | 0.5694 | -0.0275 |
| | | SCC | 0.5882 | 0.5556 | 0.5714 | (0.3000-0.6866) | (0.3421-0.7822) | (-0.3889-0.3652) |
| | Patient | AC | 0.4444 | 0.5000 | 0.4706 | 0.5227 | 0.6364 | 0.0449 |
| | | SCC | 0.6000 | 0.5455 | 0.5714 | (0.2786-0.7502) | (0.3690-0.8847) | (-0.4203-0.4781) |
| **CTransPath** | Patch | AC | 0.5057 | 0.4433 | 0.4725 | 0.5766 | 0.6102 | 0.1573 |
| | | SCC | 0.6558 | 0.7099 | 0.6818 | (0.5462-0.6054) | (0.5740-0.6450) | (0.0942-0.2173) |
| | Slide | AC | 0.4615 | 0.5000 | 0.4800 | 0.5556 | 0.5833 | 0.1098 |
| | | SCC | 0.6471 | 0.6111 | 0.6286 | (0.3732-0.7500) | (0.3644-0.7991) | (-0.2467-0.4732) |
| | Patient | AC | 0.5714 | 0.5000 | 0.5333 | 0.6136 | 0.6477 | 0.2326 |
| | | SCC | 0.6667 | 0.7273 | 0.6957 | (0.3555-0.8184) | (0.3778-0.9091) | (-0.2676-0.6445) |
| **H-optimus-0** | Patch | AC | 0.5469 | 0.4408 | 0.4881 | 0.5981 | 0.6894 | 0.2057 |
| | | SCC | 0.6687 | 0.7555 | 0.7094 | (0.5692-0.6289) | (0.6571-0.7236) | (0.1441-0.2716) |
| | Slide | AC | 0.6364 | 0.5833 | 0.6087 | 0.6806 | 0.7500 | 0.3671 |
| | | SCC | 0.7368 | 0.7778 | 0.7568 | (0.4976-0.8501) | (0.5535-0.9095) | (-0.0049-0.6999) |
| | Patient | AC | 0.6250 | 0.6250 | 0.6250 | 0.6761 | 0.7500 | 0.3523 |
| | | SCC | 0.7273 | 0.7273 | 0.7273 | (0.4499-0.8869) | (0.4889-0.9545) | (-0.0959-0.7738) |
| **H-optimus-1** | Patch | AC | 0.5152 | 0.4685 | 0.4908 | 0.5867 | 0.6612 | 0.1766 |
| | | SCC | 0.6645 | 0.7049 | 0.6841 | (0.5558-0.6175) | (0.6293-0.6948) | (0.1133-0.2391) |
| | Slide | AC | 0.5385 | 0.5833 | 0.5600 | 0.6250 | 0.7176 | 0.2472 |
| | | SCC | 0.7059 | 0.6667 | 0.6857 | (0.4364-0.8230) | (0.5227-0.9034) | (-0.1321-0.6142) |
| | Patient | AC | 0.6250 | 0.6250 | 0.6250 | 0.6761 | 0.7841 | 0.3523 |
| | | SCC | 0.7273 | 0.7273 | 0.7273 | (0.4277-0.8669) | (0.5427-0.9762) | (-0.1489-0.7246) |
| **hibou-L** | Patch | AC | 0.5512 | 0.4610 | 0.5021 | 0.6048 | 0.6570 | 0.2177 |
| | | SCC | 0.6748 | 0.7487 | 0.7098 | (0.5744-0.6351) | (0.6203-0.6913) | (0.1544-0.2787) |
| | Slide | AC | 0.6154 | 0.6667 | 0.6400 | 0.6944 | 0.7083 | 0.3845 |
| | | SCC | 0.7647 | 0.7222 | 0.7429 | (0.5138-0.8661) | (0.5093-0.8978) | (0.0272-0.7321) |
| | Patient | AC | 0.6667 | 0.7500 | 0.7059 | 0.7386 | 0.7727 | 0.4719 |
| | | SCC | 0.8000 | 0.7273 | 0.7619 | (0.5104-0.9231) | (0.5235-0.9556) | (0.0163-0.8090) |
| **Kaiko** | Patch | AC | 0.4452 | 0.3476 | 0.3904 | 0.5288 | 0.5735 | 0.0608 |
| | | SCC | 0.6191 | 0.7099 | 0.6614 | (0.4962-0.5562) | (0.5373-0.6095) | (-0.0082-0.1192) |
| | Slide | AC | 0.5833 | 0.5833 | 0.5833 | 0.6528 | 0.6111 | 0.3056 |
| | | SCC | 0.7222 | 0.7222 | 0.7222 | (0.4722-0.8230) | (0.3883-0.8215) | (-0.0565-0.6361) |
| | Patient | AC | 0.5556 | 0.6250 | 0.5882 | 0.6307 | 0.6591 | 0.2584 |
| | | SCC | 0.7000 | 0.6364 | 0.6667 | (0.3929-0.8390) | (0.3809-0.9000) | (-0.2094-0.6746) |
| **Phikon** | Patch | AC | 0.5114 | 0.4534 | 0.4806 | 0.5817 | 0.6292 | 0.1672 |
| | | SCC | 0.6599 | 0.7099 | 0.6840 | (0.5481-0.6132) | (0.5923-0.6641) | (0.1002-0.2306) |
| | Slide | AC | 0.5833 | 0.5833 | 0.5833 | 0.6528 | 0.6204 | 0.3056 |
| | | SCC | 0.7222 | 0.7222 | 0.7222 | (0.4603-0.8301) | (0.3900-0.8413) | (-0.0811-0.6591) |
| | Patient | AC | 0.6250 | 0.6250 | 0.6250 | 0.6761 | 0.6591 | 0.3523 |
| | | SCC | 0.7273 | 0.7273 | 0.7273 | (0.4277-0.8669) | (0.3750-0.8974) | (-0.1489-0.7246) |
| **ProvGigaPath** | Patch | AC | 0.4899 | 0.4282 | 0.4570 | 0.5649 | 0.6214 | 0.1333 |
| | | SCC | 0.6470 | 0.7015 | 0.6731 | (0.5350-0.5958) | (0.5865-0.6574) | (0.0712-0.1979) |
| | Slide | AC | 0.5833 | 0.5833 | 0.5833 | 0.6528 | 0.6898 | 0.3056 |
| | | SCC | 0.7222 | 0.7222 | 0.7222 | (0.4667-0.8349) | (0.4829-0.8704) | (-0.0667-0.6682) |
| | Patient | AC | 0.6250 | 0.6250 | 0.6250 | 0.6761 | 0.7159 | 0.3523 |
| | | SCC | 0.7273 | 0.7273 | 0.7273 | (0.4277-0.8669) | (0.4522-0.9333) | (-0.1489-0.7246) |
| **TissueConcepts** | Patch | AC | 0.4454 | 0.3904 | 0.4161 | 0.5325 | 0.5585 | 0.0667 |
| | | SCC | 0.6231 | 0.6745 | 0.6478 | (0.5028-0.5640) | (0.5243-0.5956) | (0.0058-0.1315) |
| | Slide | AC | 0.5000 | 0.5000 | 0.5000 | 0.5833 | 0.6065 | 0.1667 |
| | | SCC | 0.6667 | 0.6667 | 0.6667 | (0.3995-0.7634) | (0.3750-0.8133) | (-0.2019-0.5233) |
| | Patient | AC | 0.6250 | 0.6250 | 0.6250 | 0.6761 | 0.6932 | 0.3523 |
| | | SCC | 0.7273 | 0.7273 | 0.7273 | (0.4464-0.9000) | (0.3974-0.9333) | (-0.1207-0.7966) |
| **UNI2-h** | Patch | AC | 0.5479 | 0.5038 | 0.5249 | 0.6128 | 0.6783 | 0.2291 |
| | | SCC | 0.6848 | 0.7218 | 0.7028 | (0.5816-0.6447) | (0.6448-0.7115) | (0.1658-0.2940) |
| | Slide | AC | 0.5625 | 0.7500 | 0.6429 | 0.6806 | 0.7037 | 0.3546 |
| | | SCC | 0.7857 | 0.6111 | 0.6875 | (0.5000-0.8421) | (0.4907-0.8889) | (0.0000-0.6804) |
| | Patient | AC | 0.6000 | 0.7500 | 0.6667 | 0.6932 | 0.7273 | 0.3820 |
| | | SCC | 0.7778 | 0.6364 | 0.7000 | (0.4667-0.8944) | (0.4545-0.9432) | (-0.0690-0.7889) |
| **Virchow2** | Patch | AC | 0.5232 | 0.4257 | 0.4694 | 0.5830 | 0.6417 | 0.1735 |
| | | SCC | 0.6582 | 0.7403 | 0.6968 | (0.5544-0.6131) | (0.6072-0.6761) | (0.1129-0.2349) |
| | Slide | AC | 0.5556 | 0.4167 | 0.4762 | 0.5972 | 0.6898 | 0.2079 |
| | | SCC | 0.6667 | 0.7778 | 0.7179 | (0.4258-0.7656) | (0.4865-0.8731) | (-0.1545-0.5477) |
| | Patient | AC | 0.6667 | 0.5000 | 0.5714 | 0.6591 | 0.7045 | 0.3380 |
| | | SCC | 0.6923 | 0.8182 | 0.7500 | (0.4261-0.8467) | (0.4318-0.9286) | (-0.1512-0.7246) |

## 5. p-values tables (patient-level)

*Table S75: Patient-level p-values for ResNet50 compared to all foundation models for NSCLC vs. MET.*

| Architecture | Logreg | | | | | | MLP | | | | | |
|---|---|---|---|---|---|---|---|---|---|---|---|---|
| | BA | | AUROC | | MCC | | BA | | AUROC | | MCC | |
| | p | adj. p | p | adj. p | p | adj. p | p | adj. p | p | adj. p | p | adj. p |
| CONCH | 0.639 | 1.000 | 0.620 | 1.000 | 0.312 | 1.000 | 0.282 | 1.000 | 0.404 | 1.000 | 0.363 | 1.000 |
| CTransPath | 0.217 | 1.000 | 0.694 | 1.000 | 0.158 | 1.000 | 0.559 | 1.000 | 0.874 | 1.000 | 0.988 | 1.000 |
| H-optimus-0 | 0.896 | 1.000 | 0.851 | 1.000 | 0.544 | 1.000 | 0.241 | 1.000 | 0.298 | 1.000 | 0.594 | 1.000 |
| H-optimus-1 | 0.639 | 1.000 | 0.865 | 1.000 | 0.312 | 1.000 | 0.557 | 1.000 | 0.726 | 1.000 | 0.985 | 1.000 |
| hibou-L | 0.217 | 1.000 | 0.514 | 1.000 | 0.158 | 1.000 | 0.558 | 1.000 | 0.891 | 1.000 | 0.978 | 1.000 |
| Kaiko | 0.217 | 1.000 | 0.686 | 1.000 | 0.158 | 1.000 | 0.776 | 1.000 | 0.580 | 1.000 | 0.854 | 1.000 |
| Phikon | 0.896 | 1.000 | 1.000 | 1.000 | 0.544 | 1.000 | 0.241 | 1.000 | 0.591 | 1.000 | 0.594 | 1.000 |
| ProvGigaPath | 0.650 | 1.000 | 0.740 | 1.000 | 0.915 | 1.000 | 0.241 | 1.000 | 0.815 | 1.000 | 0.594 | 1.000 |
| TissueConcepts | 0.698 | 1.000 | 0.315 | 1.000 | 0.666 | 1.000 | 0.610 | 1.000 | 0.140 | 1.000 | 0.986 | 1.000 |
| UNI2-h | 0.896 | 1.000 | 0.848 | 1.000 | 0.544 | 1.000 | 0.254 | 1.000 | 0.592 | 1.000 | 0.565 | 1.000 |
| Virchow2 | 0.130 | 1.000 | 0.908 | 1.000 | 0.067 | 0.736 | 0.779 | 1.000 | 0.844 | 1.000 | 0.278 | 1.000 |

*Table S76: Patient-level p-values for ResNet50 compared to all foundation models for AC vs. SCC.*

| Architecture | Logreg | | | | | | MLP | | | | | |
|---|---|---|---|---|---|---|---|---|---|---|---|---|
| | BA | | AUROC | | MCC | | BA | | AUROC | | MCC | |
| | p | adj. p | p | adj. p | p | adj. p | p | adj. p | p | adj. p | p | adj. p |
| CONCH | 0.683 | 1.000 | 0.083 | 0.414 | 0.653 | 1.000 | 0.761 | 1.000 | 0.661 | 1.000 | 0.729 | 1.000 |
| CTransPath | 0.029 | 0.319 | 0.093 | 0.414 | 0.032 | 0.352 | 0.714 | 1.000 | 0.643 | 1.000 | 0.683 | 1.000 |
| H-optimus-0 | 0.206 | 1.000 | 0.028 | 0.313 | 0.212 | 1.000 | 0.389 | 1.000 | 0.117 | 1.000 | 0.353 | 1.000 |
| H-optimus-1 | 0.125 | 1.000 | 0.037 | 0.331 | 0.107 | 0.962 | 0.391 | 1.000 | 0.060 | 0.661 | 0.354 | 1.000 |
| hibou-L | 1.000 | 1.000 | 0.046 | 0.357 | 1.000 | 1.000 | 0.079 | 0.868 | 0.066 | 0.661 | 0.061 | 0.670 |
| Kaiko | 0.710 | 1.000 | 0.067 | 0.401 | 0.691 | 1.000 | 0.728 | 1.000 | 0.575 | 1.000 | 0.700 | 1.000 |
| Phikon | 0.206 | 1.000 | 0.172 | 0.414 | 0.212 | 1.000 | 0.391 | 1.000 | 0.600 | 1.000 | 0.354 | 1.000 |
| ProvGigaPath | 0.206 | 1.000 | 0.045 | 0.357 | 0.212 | 1.000 | 0.391 | 1.000 | 0.192 | 1.000 | 0.354 | 1.000 |
| TissueConcepts | 0.223 | 1.000 | 0.810 | 0.810 | 0.197 | 1.000 | 0.318 | 1.000 | 0.422 | 1.000 | 0.298 | 1.000 |
| UNI2-h | 0.084 | 0.839 | 0.031 | 0.313 | 0.095 | 0.949 | 0.264 | 1.000 | 0.132 | 1.000 | 0.211 | 1.000 |
| Virchow2 | 0.239 | 1.000 | 0.097 | 0.414 | 0.198 | 1.000 | 0.423 | 1.000 | 0.266 | 1.000 | 0.362 | 1.000 |

## 6. Distribution of patch numbers

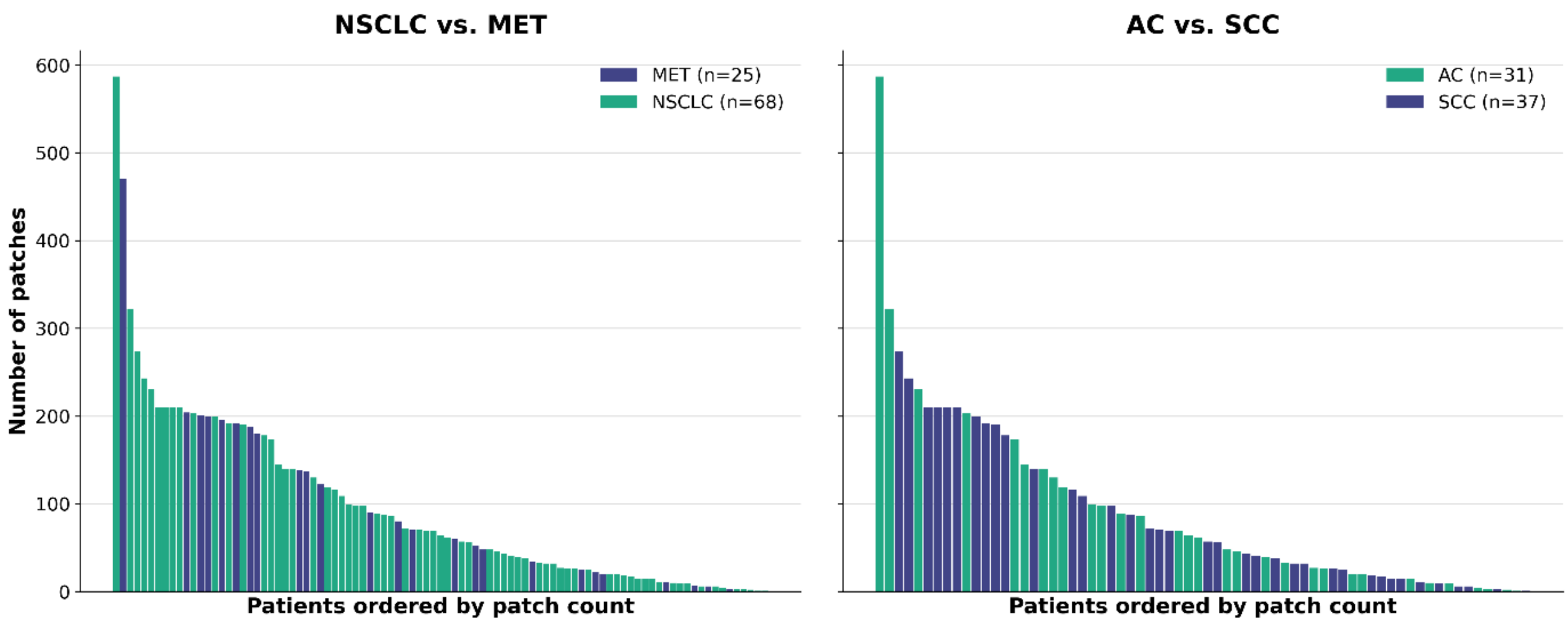


*Figure S9: Distribution of number of patches per patient for NSCLC vs. MET and AC vs. SCC.*
